# *Hepatozoon* infections in domestic and wild Carnivora: etiology, prevalence, clinical disease, diagnosis and treatment, and redescription of *Hepatozoon silvestris*, *H. martis*, and *H. ursi*

**DOI:** 10.1186/s13071-025-06977-8

**Published:** 2025-09-24

**Authors:** Jitender P. Dubey, Amer Alić, Adnan Hodžić, Jocelyn Lopez-Flores, Gad Baneth

**Affiliations:** 1 United States Department of Agriculture, Agricultural Research Service, Animal Parasitic Diseases Laboratory, Beltsville Agricultural Research Centre, Beltsville, MD 20705-2350 USA; 2 Department of Clinical Sciences of Veterinary Medicine, Faculty of Veterinary Medicine, University of Sarajevo, 71000 Sarajevo, Bosnia and Herzegovina; 3 Centre for Microbiology and Environmental Systems Science (CMESS), Department of Microbiology and Ecosystem Science, Division of Microbial Ecology (DoME), University of Vienna, 1030 Vienna, Austria; 4 Koret School of Veterinary Medicine, The Hebrew University of Jerusalem, P. O. Box 12, 7610001 Rehovot, Israel

**Keywords:** Arthropod-borne disease, Wild carnivores, Dogs, Cats, Taxonomy, Reservoir host, Life cycle, Treatment

## Abstract

*Hepatozoon* spp. are common pathogens in dogs and other Carnivora in many parts of the world, especially in the tropics. There is considerable taxonomic debate concerning the *Hepatozoon* species infecting Carnivora. Morphological descriptions of several *Hepatozoon* species are inadequate and their validity is questionable. Additionally, different terminology has been used for the description of life cycle stages. Here, we provide a comprehensive review of the *Hepatozoon* species in the Carnivora, using a uniform terminology. Worldwide prevalence of clinical and subclinical *Hepatozoon* infections for the past century is tabulated and critically evaluated. We also review the epizootiology, clinical signs, diagnosis, and treatment of hepatozoonosis in the Carnivora. The morphology and life cycles of seven valid species with known merogonic stages (*Hepatozoon americanum*, *H. canis*, *H. felis*, *H. martis*, *H. rufi*, *H. silvestris*, *H. ursi*) are summarized in a table using standard terminology. Additional information on *H. apri*, *H. martis*, and *H. silvestris* life cycle stages is provided. Information lacking for *H. procyonis*, *H. luiperdjie* and *H. ingwe* is discussed. The relevance of *H. mustelis*, *H. banethi* and *H. ewingi* is discussed and they are considered as invalid species. For the benefit of future researchers, worldwide reports of prevalence, clinical disease, diagnosis, and treatment of *Hepatozoon* infections in domestic and wild Carnivora for the past century are summarized in tables alphabetically and chronologically for each country. Co-infections of *H. canis*, *H. americanum*, *H. felis*, and *H. silvestris* are summarized and discussed. The role of *Hepatozoon* infections causing clinical illness in wild Carnivora is discussed, particularly for red foxes, coyotes, and mustelids.

**Graphical Abstract:**

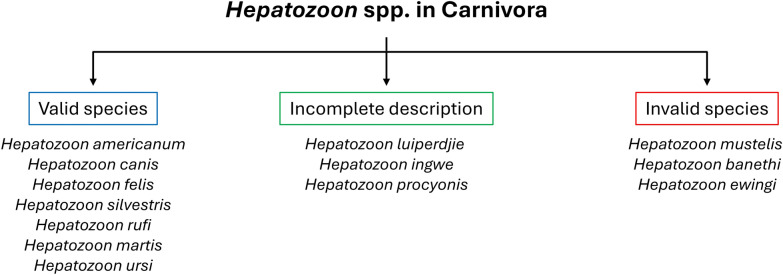

## Background

Infections with species of the protozoan genus *Hepatozoon* are prevalent worldwide, and they parasitize both ectotherms and endotherms [1]. *Hepatozoon* species have two-host life cycles, with sexual stages in arthropod vectors and asexual stages in vertebrate hosts. The distribution of *Hepatozoon* species is linked to the geographical range of the vectors, and there is a risk of spillover of *Hepatozoon* spp. from wildlife to domestic animals [2–10] and their possible exchange among reptiles and mammals [11, 12].

There are more than 300 species in the genus *Hepatozoon*, with most found in ectotherms [1]. *Hepatozoon* species infecting ectotherms are biologically distinct from those of endotherms; in ectotherms, the gamonts are located primarily in erythrocytes, whereas those of endotherms occur mostly in leukocytes [1]. The present paper is focused on *Hepatozoon* species in endotherms, mainly in Carnivora, while those infecting rodents are not further addressed. It adds perspectives to the subject previously reviewed by others [13–25].

Among *Hepatozoon* species infecting Carnivora*, H. canis* is one of the species that was recognized more than a century ago [26]. It is the most widely distributed and apparently has a great host range; it has been reported in 13 wild mammal species currently [24]. There is taxonomic debate concerning the *Hepatozoon* species infecting Carnivora. Morphological descriptions of several *Hepatozoon* species are inadequate, and the validity of some species is questionable. Here, we reexamined descriptions of named species using a recently proposed uniform terminology [27]. Additionally, the prevalence, clinical spectrum, diagnosis, and treatment for hepatozoonosis in the Carnivora are outlined. For the benefit of future researchers, worldwide reports on the prevalence, clinical disease, diagnosis, and treatment of *Hepatozoon* infections in domestic and wild Carnivora for the past century are summarized in tables alphabetically and chronologically for each country; many of these reports are not easily accessible.

## General life cycle and terminology

The life cycle of *H. canis* is used herein as an example. After ingestion of infected blood by an invertebrate host, the sexual development of *H. canis* occurs in the gut wall of the tick, producing large oocysts (that can be macroscopic) in the hemocoel which contain hundreds of sporozoites enclosed in sporocysts (Table 1). Dogs become infected with *Hepatozoon* spp. by ingesting infected ticks, most likely while grooming or preying on smaller animals parasitized by ticks [28]. Sporocysts are released mechanically from oocysts with maceration of tick tissues, and sporozoites are released when sporocysts excyst when encountering bile in the duodenum of the dog. Sporozoites invade canine tissues and reproduce asexually, producing meronts that contain merozoites, which eventually invade blood leukocytes, forming gamonts. Additionally, carnivorism through ingestion of tissue of paratenic hosts containing cysts is another possible mode of transmission [27, 28]. In the paratenic hosts, the parasite persists but does not multiply. For example, rodents and rabbits can act as paratenic hosts for *H. americanum*; only tissue cysts containing sporozoites are found [29, 30]. Vertical transmission can also occur in *H. canis* infections [31–33]. In naturally infected ticks, there is transstadial but no transovarial transmission [34].

**Table 1:** *Hepatozoon* species in the Carnivora with known merogonic tissue stages

Character	*H. americanum*	*H. canis*	*H. felis*	*H. martis*	*H. rufi*	*H. silvestris*	*H. ursi*
Authority	Vincent-Johnson, Macintire, Lindsay, Lenz, Baneth, Shkap, 1997	(James, 1905) Wenyon, 1926	(Patton, 1908) Wenyon, 1926	Hodžić, Alić, Beck, Beck, Huber, Otranto, Baneth, Duscher, 2018	Dubey, Gupta, de Araujo, Kwok, Rosenthal, 2024	Hodžić, Alić, Prašović, Otranto, Baneth, Duscher, 2017	Kubo, Uni, Agatsuma, Nagataki, Panciera, Tsubota, Nakamura, Sakai, Masegi, Yanai, 2008
Main host	Domestic dog (*Canis familiaris*)	Domestic dog (*Canis familiaris*)	Domestic cat (*Felis catus*)	Pine marten (*Martes martes*)	Bobcat (*Lynx rufus*)	European wildcat (*Felis silvestris*)	Japanese black bear (*Ursus thibetanus japonicus*)
Other hosts	Coyote (*Canis latrans*)	Domestic cat, wild canids, lions	Wild felids, European wildcat (*F. silvestris*), Asiatic lion (*Panthera leo persica*), Indian leopard (*Panthera pardus fusca*), royal Bengal tiger (*Panthera tigris tigris*), caracal (*Caracal caracal*), African lion (*Panthera leo*), serval (*Leptailurus serval*)	Beech marten (*Martes foina*), European polecat (*Mustela putorius*), European badger (*Meles meles*)	Unknown	Domestic cat	None
Geographical distribution	North America	Worldwide	Probably worldwide	Europe	USA	Europe, USA	Japan
Definitive host	Gulf Coast tick, *Amblyomma maculatum* (3-host tick but feeds on several hosts, including dogs)	Brown dog tick, *Rhipicephalus sanguineus* sensu lato (3-host tick feeds on dogs and other mammals);* Rhipicephalus turanicus*; *Amblyomma ovale*	Unknown	Unknown	Unknown	Unknown	*Haemaphysalis japonica*; *Haemaphysalis flava*
Oocysts	205–290 µm	260 × 300 µm	Unknown	Unknown	Unknown	Unknown	263 × 234 and 331 × 231 µm
Sporocysts per oocyst	260–1040	57–129	Unknown	Unknown	Unknown	Unknown	40–50
Sporocyst size	18–39 µm	14 × 32 µm	Unknown	Unknown	Unknown	Unknown	31 × 27 µm
Sporozoites per sporocyst	10–26	7 or 8	Unknown	Unknown	Unknown	Unknown	8–16
Sporozoite dimensions	15 × 5 µm	15.8 × 3.1 µm	Unknown	Unknown	Unknown	Unknown	12 × 3.5 µm
Merogonic site	Mainly muscle, host cell monocyte	Mainly bone marrow, spleen, lymph nodes, liver, lungs, kidneys, host cells—neutrophil and monocyte	Heart, skeletal muscle, lung	Mainly heart and muscles	Heart, tongue, limb muscle	Heart, skeletal muscle, lung, not in tongue	Lung
Type 1 meront	No	Yes (2–4 merozoites,15 × 6.7 µm)	No	Yes	Present (10.25 ± 0.75 × 3.75 ± 0.75 µm)	11.5 × 2.5 µm merozoites	No
Type 2 meront	Rare. 188 × 162 µm, contain blastophores. Onion skin stage 130–280 × 70–245 µm	30 × 29 µm	Present (36.85 ± 6.15 × 34.5 ± 3.8 µm)	36.7 × 30.5 µm	Up to 39 × 24 µm	Present (31.7 ± 4.2 × 22.0 ± 4.6 µm)	45 × 42 µm
No. of merozoites	117 or more	20–30	Not stated	20–50	Up to 44	20–30	80–130, residual body, blastomeres
Merozoite size	7.5 × 2.7 µm	10.7 × 1.7 µm	7.5 × 1.9 µm	6.5–8.0 x ~ 3.0 µm	8.6 ± 0.4 × 2.25 ± 0.35 µm	~ 7.5 × 2.5 µm	5.7–7.8 × 1.5–2.4 µm
Type 3 meront	Absent	Absent	Absent	Absent	Present	Absent	Unknown
Gamonts	8.8 × 3.9 µm	9.7 × 1.4 µm	10.5 ± 0.6 × 4.7 ± 0.8 µm	8.4 × 5.9 µm	Unknown	10–12 × 3 μm	11 × 3.3 μm, characteristic protrusion
Parasitemia	Low (< 0.1% of circulating white blood cells)	Prolonged (up to 70% of circulating white blood cells)	Yes, leukocytes	Yes, leukocytes	No data	Yes, neutrophils	Yes, leukocytes
Paratenic hosts	Rodents, rabbits	Dog	Unknown	Unknown	Unknown	Unknown	Unknown
Tissue cyst	In paratenic hosts, monozoic or dizoic, 12.8 × 16.6 µm	21 × 17.1 µm	Unknown	Unknown	Unknown	*F. silvestris,* monozoic or dizoic, 15–17 µm long × 12 µm wide	Unknown
Congenital transmission	Unknown	Yes	Unknown	Unknown	Unknown	Unknown	Unknown
Transmission by carnivorism	Yes	Unknown	Unknown	Unknown	Unknown	Unknown	Unknown
Clinical disease	Yes	Yes	Yes	Unknown	Unknown	Yes	Unknown
Main references	[27, 155, 156]	[28]	[110]	[45], present study	[157]	[46, 154], present study	[47, 158]

### Asexual stages of *Hepatozoon* and terminology

There is considerable debate and uncertainty concerning the terminology used to define different stages of *Hepatozoon*. This topic was recently reviewed [27] and summarized.

#### Trophozoite

Trophozoite is an old term, used to describe the first parasitic stage of a coccidian parasite [35]. After the sporozoite or merozoite enters the host cell, it undergoes rounding and loses some of its organelles. This round stage was called trophozoite [35]. Currently, it is a unizoite meront or zoite, irrespective of its outcome as a gamont or meront [36].

#### Meronts

Based on the life cycles of *Hepatozoon* spp. in ectotherms, meronts were called macromeronts (also called Y meronts), which contain larger-sized merozoites (macromerozoites), and micromeronts, which contain numerous smaller merozoites (called micromerozoites) [1]. Dubey and Baneth [27] proposed replacing macromeronts and micromeronts with type 1 and type 2 meronts, respectively. The terms macromeronts and micromeronts are confusing, because not all macromerozoites are large, especially in *Hepatozoon* spp. of mammals (Table 1). Also, *H. americanum* meronts were called onion skin cysts/schizonts (discussed further below). The term meront is used here for all asexual stages, irrespective of the divisional process. This terminology is followed in the present paper to compare different *Hepatozoon* species.

Three morphological types of meronts have been described for *Hepatozoon* species of mammals. Type 1 meronts are small but contain large merozoites. Little is known of the function and purpose of these meronts (Table 1). Type 2 meront is the common stage recognized in all *Hepatozoon* species. The nuclei are often arranged at the periphery of the meront, and merozoites develop peripherally, often around a residual body; they were previously termed wheel-spoke meronts in species where a typical wheel shape is found. In two species (*H. ursi* of the Japanese bear and *H. americanum* of dogs), there are many nuclei in meronts arranged in groups or blastomeres. Type 2 meronts are surrounded by a glistening white capsule. Type 3 meronts were recently described for *H. rufi* of bobcat, and they lack the glistening capsule (Table 1). Type 2 meronts of *H. americanum* are morphologically distinct from those of other *Hepatozoon* species. The meront stage of *H. americanum* was initially recognized by an unusual structure termed “onion skin cyst” [37–40]. There is a considerable lack of understanding concerning the merogonic development of *H. americanum* and the development of the onion skin meront, which was recently further reviewed and summarized [27].

#### Granulomas

Granulomas are formed in some *Hepatozoon* species following the rupture of meronts and host reaction against liberated merozoites [17, 18]. Many single zoites are present in granulomas. These zoites form either a new generation of merozoites or early gamonts. Gamonts from the granulomas enter leukocytes and can be found thereafter in blood. It is uncertain how many merogonic cycles occur in the life cycle of hepatozoans. It has been suggested that in *H. americanum*, merogony can persist for a long period (at least 5.5 years), as evidenced by a muscle biopsy of a dog maintained in a tick-free enclosure [41]. As the appearance of gamonts in the blood of dogs infected with *H. canis* is also long-lasting, and experimentally and naturally infected dogs are aparasitemic for months, it is likely that the same also occurs in *H. canis* infection. The lifespan of neutrophils and monocytes (the host cells) in the blood is short and can last a few hours (in the case of neutrophils) to a few days (for monocytes).

#### Tissue cysts

Monozoic tissue cysts were first found in *Hepatozoon* spp. of ectotherms, predominantly in the liver (reviewed in [27]). Monozoic or dizoic tissue cysts have also been reported in *H. canis* and *H. americanum* and other *Hepatozoon* spp. in domestic and wildlife mammalian hosts [13, 16, 30, 42–45]. However, unlike *Hepatozoon* spp. tissue cysts in ectotherms, tissue cysts in *Hepatozoon* spp. of mammals contain only one or two zoites (cystozoites or sporozoites). With light microscopy, these tissue cysts contain elongated zoites which are periodic acid–Schiff (PAS)-positive, indicating that they contain sporozoites. The monozoic and dizoic tissue cysts in *H. americanum* contain amylopectin that stain positive with PAS, typical of sporozoites [30].

The role of tissue cysts in the life cycle of *Hepatozoon* species infecting Carnivora is uncertain. An outbreak of clinical hepatozoonosis in a pack of hunting beagles infected with *H. americanum* was circumstantially associated with eating a hunted rabbit [29]. However, there is no confirmed report of the presence of tissue cysts in naturally infected transport/paratenic host of any *Hepatozoon* species of mammals. Carnivorism in the *Hepatozoon* genus may be an evolutionary link between parasites of ectotherms and endotherms. In certain *Hepatozoon* species infecting snakes, after ingesting the invertebrate host, the parasite can multiply in intermediate hosts and the vertebrate host can become infected by eating infected intermediate hosts. For example, in the case of *H. domerguei* of snakes, sporogony occurs in mosquitoes, and both merogony and gametogony can occur in the visceral tissues of lizards (optional intermediate hosts), and snakes can become infected by eating infected mosquitoes and also lizards [1].

## Evaluation of *Hepatozoon* species of Carnivora

### Valid/established species with known merogonic tissues stages

Information regarding the biology of seven *Hepatozoon* species is summarized in Table 1. Of these, the full life cycle is known only for *H. canis* and *H. americanum*, and their cycles were completed experimentally. Available information on invertebrate hosts (definitive hosts), vertebrate hosts (intermediate hosts), transport or paratenic hosts, and geographical distribution is summarized.

Here, we provide additional information on the tissue stages of *H. silvestris* [46] and *H. martis* [45] using uniform terminology and after staining tissue sections with PAS and photographing images at 1000× magnification. Additional information is also added concerning *H. ursi* [47].

#### *Hepatozoon silvestris*

**Fig. 1:**
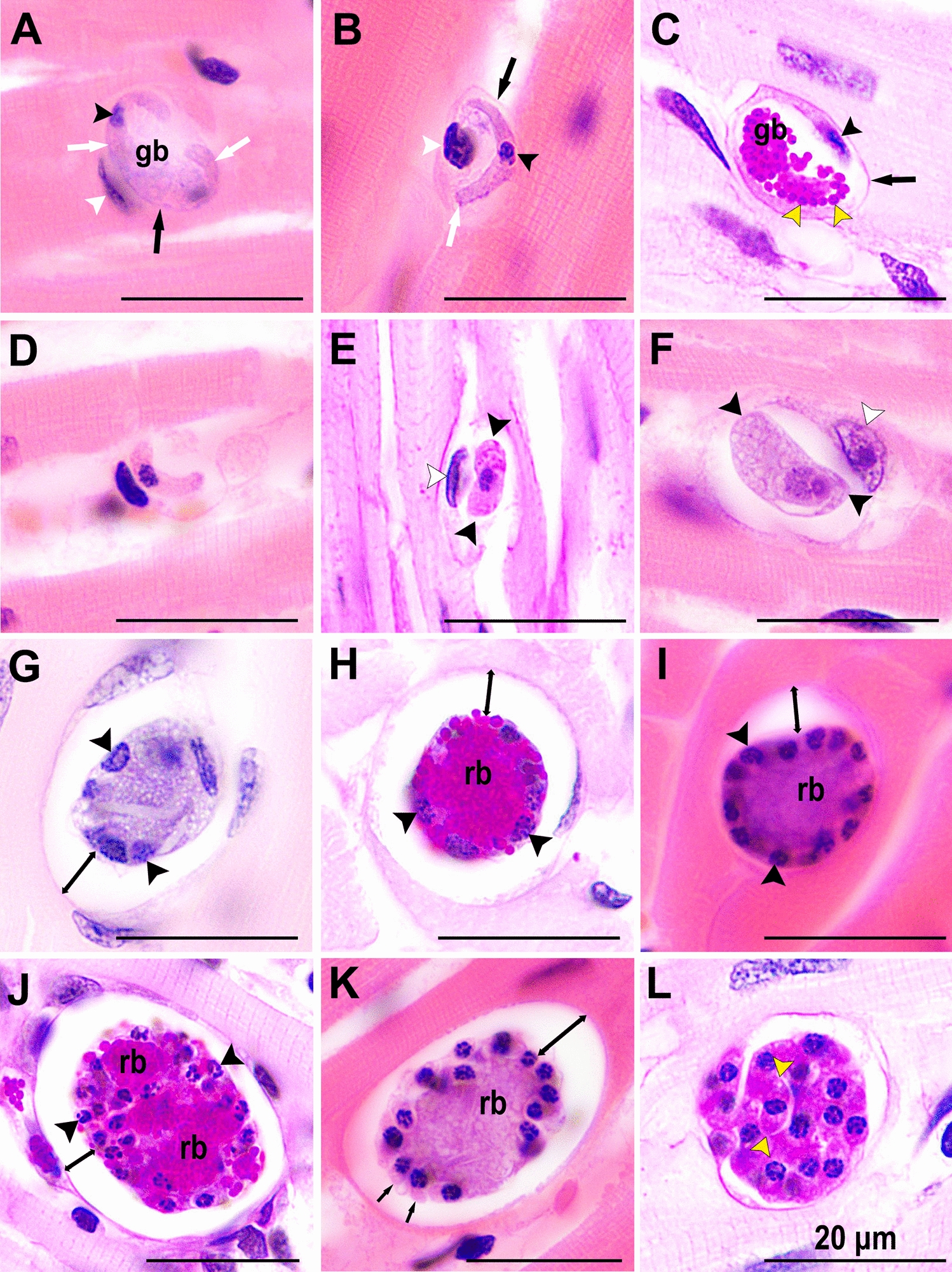
Asexual stages of *Hepatozoon silvestris* in histological sections of the myocardium of *Felis silvestris* from Bosnia and Herzegovina. Scale bar is 20 µm and applies to all parts. **A**–**C** Tissue cysts from wildcat# 412/16. **A** and **B** Hematoxylin and eosin stain, **C** periodic acid–Schiff (PAS) reaction counter stained with hematoxylin. Black arrows point to the cyst wall, white arrows point to zoites (sporozoites), black arrowheads point to sporozoite nucleus, white arrowhead points to host cell nucleus, gb = globular material. **A** Sporozoites cut partially. **B** An arc-shaped sporozoite. **C** An oval tissue cyst with thin PAS-positive cyst wall. The sporozoite is cut partially. Note large PAS-positive residuum (globules), some of them appear to have nuclei (yellow arrowheads). **D**–**L** merogonic stages; **D**, **F**, **H**–**L** cat# 412/16, **E** 152/16, **G** 115D/16. **D**, **F**, **G**, **I**, **K** hematoxylin and eosin stain; **E**, **H**, **J**, **L** PAS. White arrowheads point to host cell nucleus, double-edged bar points to capsule width, black arrowheads point to meront nuclei, opposing arrowheads point to zoite ends, rb = residual body or cytoplasmic mass. **D** Sporozoite-like curved zoite between myocardiocytes. **E** Longitudinal section of a presumed type 1 merozoite with a centrally located large vesicular nucleus. **F** Early type 2 meront with an eccentrically placed large nucleus with a prominent nucleolus. Please note the large foamy cytoplasmic mass towards the non-nuclear end. **G** Early meront with peripheral nuclei. **H** Early meront with six peripheral nuclei and a prominent PAS-positive cytoplasmic mass. **I** Meront with 12 peripheral nuclei. Note that the capsule is visible only in a small portion of the meront. **J** Meront with diffuse residual PAS-positive mass. **K** Meront with merozoites (black arrows) developing at the periphery. **L** Mature meront with merozoites without a residual body. Note one longitudinally cut merozoite (yellow arrowheads) with a central nucleus and PAS-positive granules

Tissue cysts, meronts, and gamonts were identified in tissues of naturally infected *Felis silvestris* from Bosnia and Herzegovina. Histological sections of heart, skeletal muscle, and lung were examined in this study. In the original study [46], all tissues were studied but *Hepatozoon* stages were found only in the heart and skeletal muscle. Tissue cysts were found in the myocardium, likely in cardiomyocytes (Fig. 1A–C). They were oval to pear-shaped and were limited by a thin (< 0.5 µm) PAS -positive membrane (Fig. 1C). Cysts were 15–17 µm long and up to 12 µm wide. Tissue cysts enclosed one or possibly two slender zoites (sporozoites). Zoites were 12–15 µm long and around 2 µm wide, and had a central nucleus that occupied the entire width of the zoite (Fig. 1B). In sections stained with PAS, prominent PAS-positive globules, approximately 2–3 µm wide, occupied the cyst interior (Fig. 1C). The globular material was not clear in sections stained with hematoxylin and eosin (HE) (Fig. 1B).

Merogonic development was observed in histological sections of the hearts. The earliest stage seen was an arc-shaped zoite between myocardiocytes, without a parasitophorous vacuole (Fig. 1D). The zoite was around 11.5 × 2.5 µm. It had a vesicular nucleus, as wide as the zoite and located towards the distal round (non-conoidal) end; the non-conoidal end was pointed and stained darker than the rest of the zoite (Fig. 1D). This stage was interpreted as a sporozoite. The next developmental stage observed was a stout 8.5 × 3.5 µm zoite; the nucleus was elongated and located centrally (Fig. 1E). This stage was interpreted as type 1 merozoite. Figure 1F shows the earliest confirmed uninucleate type 2 meront. The meront was 14.0 × 7.0 µm with eccentric nucleus and a prominent nucleolus; the remainder of the meront was filled with foamy cytoplasm, interpreted as the early stage of the residual body. Figure 1G shows an early meront with five or six peripherally located elongated nuclei; it was misinterpreted earlier as type 1 meront [46]. The capsule around type 2 meronts was up to 5 µm wide, and the width varied with the angle of the meront cut (Fig. 1G–I). Merozoites budded at the periphery (Fig. 1K). The residual body was not seen in mature meronts. A longitudinally cut merozoite was 7.5 × 2.5 µm, with a central round nucleus and bipolar PAS-positive granules (Fig. 1L).

In summary, the tissue cyst stage for *H. silvestris* is documented here for the first time. The cyst-like structures depicted earlier [46] are now considered unizoite meronts. Type 1 merozoites are recognized here for the first time. The size of type 2 merozoites is also updated.

**Fig. 2:**
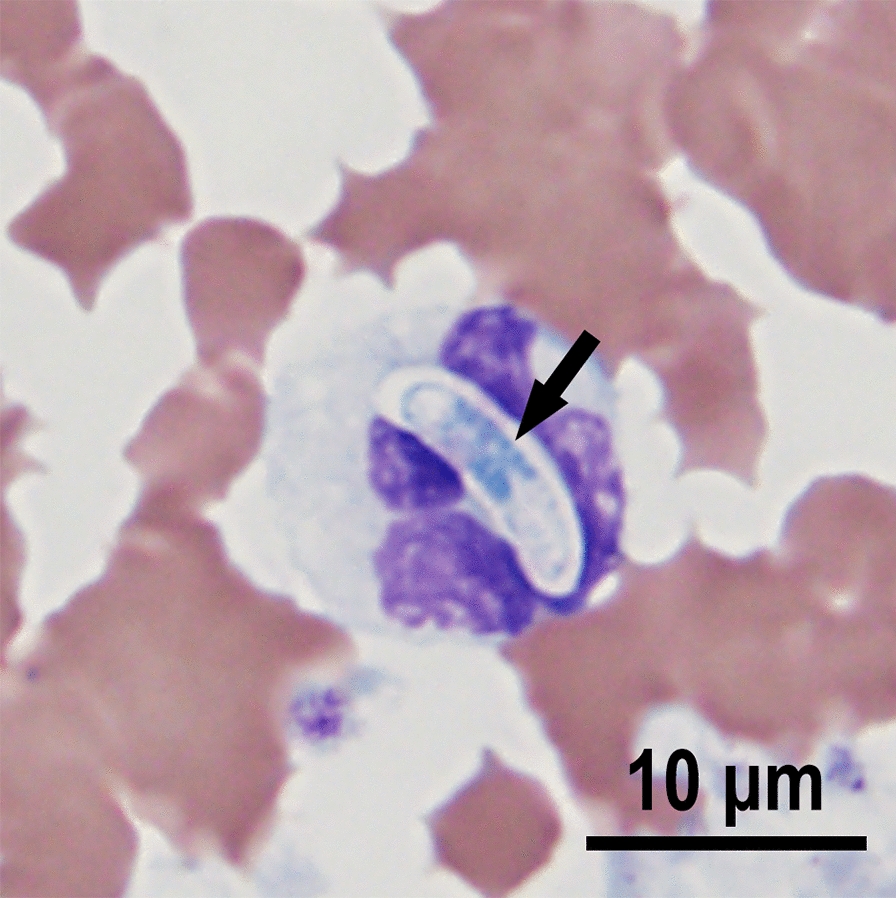
*Hepatozoon silvestris* gamont from peripheral blood of a domestic cat from the USA [155]. Wright–Giemsa stain. (Courtesy of Drs. Alys Harshbarger and Ruth Scimeca, Oklahoma State University, Oklahoma, USA)

Gamonts have not been confirmed in *F. silvestris* examined at necropsy. A gamont in peripheral blood smear of a domestic cat (*Felis catus*) from the USA with molecularly confirmed *H. silvestris* infection is shown in Fig. 2.

#### *Hepatozoon martis*

**Fig. 3:**
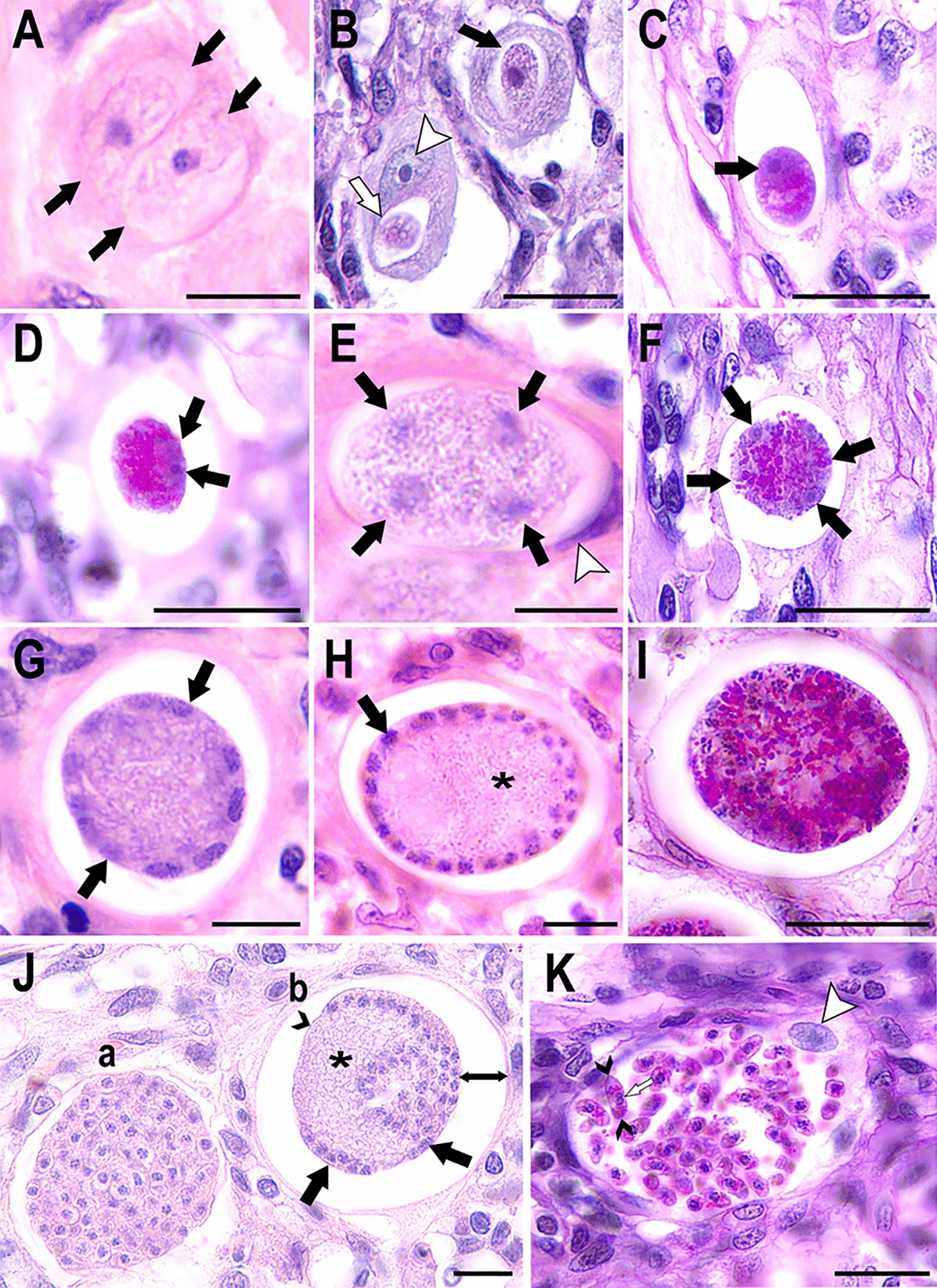
Merogonic development of *Hepatozoon martis* in the myocardium of naturally infected pine martens (*Martes martes*) from Bosnia and Herzegovina (ID for martens, **A** 27/23, **B** 32/22; **C**, **D**, **F**, **K** = 197/16, **E** 54/17, **G** 147/22, **H** 64/22, **I** 542/23, **J** 33/22). Bar = 20 µm and applies to all parts. **A**, **E**, **G**, **H**, **J** histological sections stained with hematoxylin and eosin, **B**, **C**, **D**, **F**, **I**, **K** periodic acid–Schiff (PAS) reaction counter stained with hematoxylin. White arrowheads point to indented host nuclei. **A** Type 1 meront with two stout merozoites (arrows) without a capsule. **B** Two unizoite meronts in parasitophorous vacuoles (pv). Note the longitudinally cut zoite (black arrow) and a zoite in cross section (white arrow). These zoites are PAS-negative. **C** An intracellular uninucleate meront with a large pv. Note that the zoite is oblong in shape, has an eccentric nucleus (arrow), and has PAS-positive granules. **D** An early type 2 meront with two nuclei (arrows) that are obscured by PAS-positive cytoplasm. **E** An early pear-shaped meront with four peripherally located nuclei (arrow) and amorphous eosinophilic cytoplasm. The chromatin in the nuclei is dispersed. **F** An early type 2 meront with four nuclei (arrows) that are obscured by PAS-positive mass. **G** Immature meront with peripherally located nuclei; note that nuclei are vesicular and elongated. **H** An immature meront with nuclei (arrow) arranged at the periphery of the dispersed eosinophilic cytoplasm marked by an asterisk. **I** A large immature meront with PAS-positive granules. Note the well-developed capsule. **J** Two type 2 meronts (**a**, **b**). The nuclei/merozoites in meront (**a**) fill the meront, without any residual mass. The nuclei/merozoites in meront (**b**) are cut tangentially, with eosinophilic cytoplasm on one end (asterisk). Note the absence of a residual body. Bar with arrowheads points to capsule width. **K** A large mature type 2 meront with fully developed merozoites. A longitudinally cut merozoite (opposing arrowheads) with a central nucleus (white arrow) and perinuclear PAS-positive granules

**Fig. 4:**
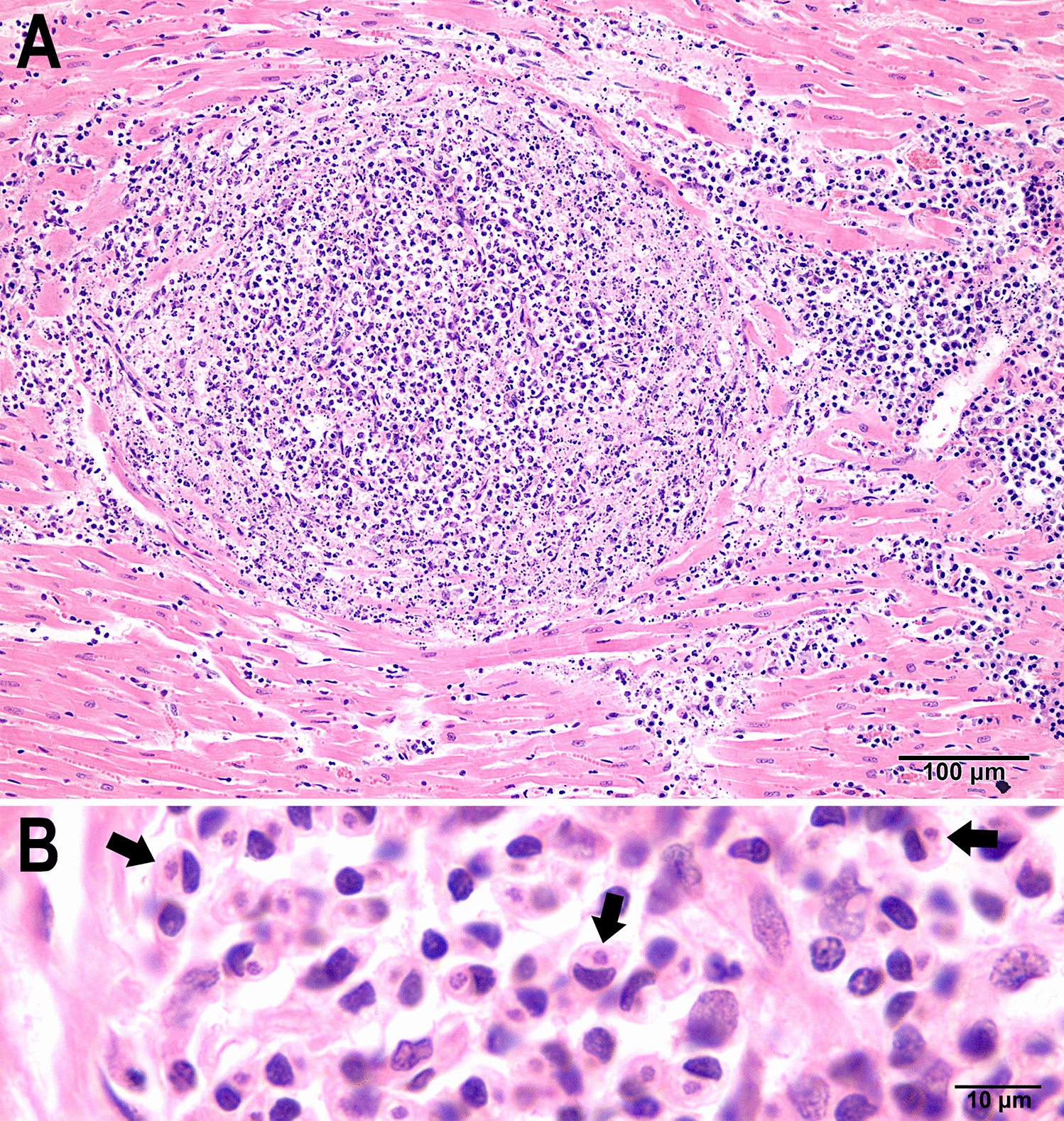
Pyogranulomatous lesions associated with *Hepatozoon martis* infection in the heart of a pine marten in Bosnia and Herzegovina. Hematoxylin and eosin stain. **A** Central core of pyogarnuloma is composed of leukocytes, many of which have dislocated nuclei by engulfed zoites of *H. martis* (not visible at this magnification)*.* At the periphery, there is massive necrosis of myocardiocytes with granular necrotic debris and degenerate leukocytes. Radially, moderate numbers of neutrophils and plasmacytes (more prominent on the right) infiltrate the surrounding myocardium. Marten 413/16. **B** Higher magnification of the central core of a lesion similar to that in the figure 4A. Note the numerous single zoites (arrows) engulfed by mononuclear host cells. Marten 64/22

Here, we provide additional details regarding the merogonic development of *H. martis* in histological sections of the hearts of naturally infected pine marten (*Martes martes*) and stone marten (*M. foina*) [45]. Histological sections of the heart, skeletal muscle, and lung were examined in this study. In the original study [45], all tissues were studied but *Hepatozoon* stages were found only in the heart, skeletal muscle, and subcutis of the scrotum of one marten. A tissue cyst stage was not recognized. Two types of meronts are described using the recently proposed terminology [27]. A type 1 meront contained two stout intracellular merozoites; a capsule was absent. The merozoites were ~ 12 × 5 µm each, with a centrally located vesicular nucleus (Fig. 3A). The earliest type 2 meronts contained single zoites with a large centrally located nucleus; this stage was surrounded by a large parasitophorous vacuole (pv); zoites were up to 15 µm long and up to 8 µm wide; these zoites were PAS-negative (Fig. 3B). This stage was previously interpreted as monozoic tissue cyst [45]. Later, early meronts were PAS-positive (Fig. 3C). Figure 3C shows a uninucleate 14.5 × 8 µm PAS-positive zoite with an eccentric nucleus. A binucleated meront is depicted in Fig. 3D; the nuclei are partly obscured by PAS-positive cytoplasm. In Fig. 3E, a well-developed pear-shaped capsule encloses four vesicular nuclei. Subsequently, the nuclei moved peripherally and the chromatin in the nuclei condensed (Fig. 3F–H). The nuclei in some more advanced meronts were located centrally or peripherally and some were arranged in groups, like blastomeres (Fig. 3J). In immature meronts, a compact residual body was absent; instead, the cytoplasm was diffusely PAS-positive (Fig. 3I). Merozoites in mature meronts were arranged haphazardly (Fig. 3K). Four longitudinally cut merozoites were 6.5–8.0 × 3.0–3.3 µm; they had a centrally located vesicular nucleus and PAS-positive granules at both its ends (Fig. 3K). Granulomas were found in muscles (Fig. 4).

In summary, we did not find *H. martis* tissue cysts. The structures labeled as cysts in the original description of *H. martis* [45] are now considered unizoite type 2 meronts. Additionally, early stages of type 2 meronts are described for the first time, probably for any *Hepatozoon* species of Carnivora. The size of longitudinally cut type 2 merozoites is markedly larger than in the original description of *H. martis* [45], perhaps due to the sectioning plane.

#### *Hepatozoon ursi*

The biology of *H. ursi* from the Japanese black bear (*Ursus thibetanus japonicus*) is interesting, because asexual stages were confined to the lungs, bears were asymptomatic, and the prevalence of *Hepatozoon* infections was very high [47]. Here, we provide additional information concerning the meronts of *H. ursi*, based on images from additional naturally infected bears from Japan.

**Fig. 5:**
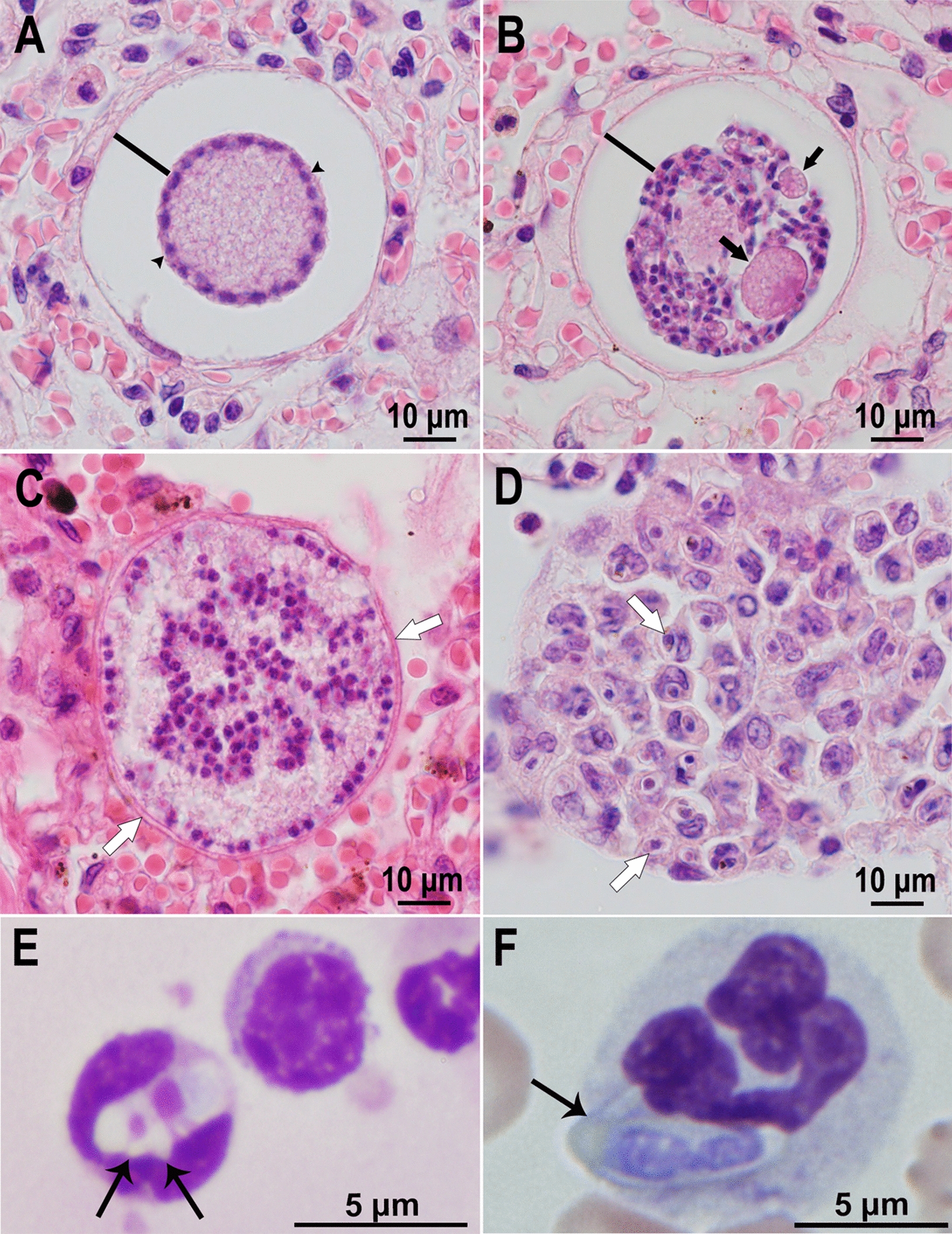
*Hepatozoon ursi* stages in the lung of a Japanese bear (*Ursus thibetanus*). Hematoxylin and eosin stain. **A** An immature meront. Note peripheral arrangement of nuclei around a large eosinophilic central body. The bar points to the thick capsule. **B** Mature meront with merozoites around eosinophilic residual bodies (arrows). The bar points to the thick capsule. **C** Mature meront with merozoites at the periphery and in the center arranged in groups. Arrows point to the thin membrane surrounding the meront; a capsule is not visible. **D** A granuloma with individual intracellular zoites, which appear to be merozoites with a central nucleus (arrows). (Courtesy of Dr. Hisashi Yoshimura, Japan). **E**, **F** Gamonts of *Hepatozoon ursi* in peripheral blood smears of Japanese bear (*Ursus thibetanus*). Giemsa stain. **E** Note a beak-like projection (arrow) in a gamont in a neutrophil. **F** A gamont in a monocyte. Note two small projections (arrows). (Courtesy of Dr. Masahito Kubo, Japan)

Meronts of *H. ursi* were found in the lung parenchyma, with minimal host response (Fig. 5). Initially, basophilic nuclei in young meronts appeared distributed peripherally around a large eosinophilic mass (Fig. 5A). Subsequently, the nuclei were arranged both at the periphery and throughout the meront, often comprising groups or blastomeres (Fig. 5C). Merozoites were formed centrally, leaving two or more eosinophilic bodies (Fig. 5B). A large, clear space (capsule) was evident around some meronts (Fig. 5A, B). However, the meront shown in Fig. 5C was surrounded by thin membranes, unlike the glistening capsule found in other *Hepatozoon* species. Granuloma formed in the lung and contained intracellular zoites that appeared to be merozoites with a large central nucleus and tapered ends (Fig. 5D). Gamonts were found in both neutrophils and monocytes (Fig. 5E, F). By transmission electron microscopy (TEM), the earliest stage of *H. ursi* was an intracellular uninucleate zoite/meront (the authors called it a trophozoite); it contained micronemes, dense granules, and rhoptries [47]. Merozoites in mature meronts were elongated with a central nucleus, one or two rhoptries, few dense granules, and numerous micronemes that were arranged both anterior and posterior to the nucleus. These findings are discussed here because it is a rare example of the ultrastructural description of *Hepatozoon* merozoites in the Carnivora. A beak-like appendage in the gamont was confirmed by TEM [47]. The description of sexual stages in ticks collected from naturally infected bears indicates that *Haemaphysalis* spp. serve as definitive hosts of *H. ursi* [47].

In summary, the development of type 2 *H. ursi* meronts was updated, and lack of inflammatory response to meronts in lungs was confirmed. The finding of more than one compact residual body is an unusual finding not reported in meronts of other *Hepatozoon* species.

### Species require additional information

#### *Hepatozoon luiperdjie* and *Hepatozoon ingwe*

These species were named from African leopards (*Panthera pardus pardus*) in South Africa, based on gamonts in blood leukocytes and molecular characteristics; merogonic tissue stages are unknown [48]. Gamonts of *H. ingwe* were 11.4 × 4.8 µm and occurred in lymphocytes with an average parasitemia of 30.8%. Recently, the sexual stages of *H. ingwe* were described in an *Ixodes* sp. tick collected from a naturally infected *P. pardus* [49]. Mature oocysts from the hemocoel of *Ixodes* sp. were 190 × 157 µm and contained 23–25 sporocysts that measured 30 × 29 µm. Each sporocyst contained around 30 sporozoites that measured 12.8 × 20.0 µm [49]. Gamonts of *H*. *luiperdjie* occurred in neutrophils and measured 11 × 4.7 µm [48]. Average parasitemia was 11%. Its sexual stages are unknown [49].

#### *Hepatozoon procyonis*

This parasite was named by Richards [50] and was from wild raccoons (*Procyon lotor*) trapped in Georgia, USA, for survey of *Trypanosoma cruzi* and *Babesia* infections. Meronts were found in histological sections of the myocardium of six of eight raccoons. Meronts were 85 × 50 µm and contained around 35 merozoites in histological sections. Merozoites were at the periphery of the meront around a residual body and were not seen in spleen sections. Gamonts were also seen in sections of the hearts and detected in peripheral blood monocytes of three raccoons. The gamonts were sausage-shaped, 9.5 × 4.3 µm, with a large central nucleus. A tail-like body was present at one pole of the gamont [50].

Richards [50] stated in his paper that type specimens of *H. procyonis* were deposited in the US National Museum from raccoon number 6030. Apparently there is a mistake, because no museum number was provided. An extensive search of the US Smithsonian records at the request of one of us (JPD) revealed no record of its deposition.

Clark et al. [51] found *H. procyonis* gamonts in 57 (88%) of 65 adult raccoons examined at necropsy in Texas, USA; there are no archived specimens for further evaluation. Gamonts (7.5 × 3.9 µm) with a tadpole morphology were present in both monocytes and neutrophils in tissue sections; gamonts were not detected in blood smears. No gross lesions were detected. Inflammatory lesions were seen in the heart, skeletal muscle, and spleen. Meronts (type 2) measuring around 31 × 22.7 µm were found in myocytes; the residual body and cytoplasm of merozoites were PAS-positive. Meronts were seen in the hearts of 44, skeletal muscle of 24, and spleens of two. The infected raccoons were apparently healthy. Little information is available concerning molecular characterization of *H. procyonis* from raccoons in the USA, except for a DNA sequence from the heart of one of four raccoons in Oklahoma [52].

A *H. procyonis*-like parasite was reported from the South American coati (*Nasua nasua*) [53]. Gamonts were found in blood smears of nine of 83 *N. nasua*. The gamonts were 10.3 × 3.2 µm and had a hook at one end. Type 2 meronts (21.6 × 18.9 µm) were found in the liver and spleen of one of two necropsied coati. A similar parasite was described from a crab-eating raccoon (*Procyon cancrivorus*) from Brazil [54] and Panama [55]. In the Panamanian raccoon, meronts were found in the myocardium, but they were half the size noted in the American raccoon [50].

A redescription/revaluation of *H. procyonosis* from the raccoon (*Procyon lotor*) from the USA is warranted, with deposition of specimens in a museum collection.

### Invalid, *nomen nudum* species

#### *Hepatozoon mustelis*

A new species, *Hepatozoon mustelis*, was proposed for a parasite found in the tissues of Siberian polecat (*Mustela eversmanii satunini*) from Russia [56]. This species should be considered *nomen nudum* for the reasons stated at the end of the paragraph. However, this unique case of hepatozoonosis deserves a comment. This reference was published as a conference proceeding and was difficult to find. Therefore, the findings are summarized here.

As part of a collaboration between the USA and the former USSR, Siberian polecats were imported in October 1975 from Russia into the wildlife center at Laurel, Maryland, USA. Three months later, a female in estrus was exposed to a male for 2 days. A month later, the dam delivered 11 offspring (kittens). Two kittens were cannibalized by the dam. Two weeks after birth, all surviving kits developed dermal lesions. Three to 5 days later, seven of the nine kittens died and were necropsied; two remained healthy and were not investigated. On necropsy, all kittens had dermal lesions. Livers were mottled and had 1–3 mm whitish discoloration. *Hepatozoon* meronts were detected in the skin and hearts of all seven, in the skeletal muscle of five, in the kidneys and lymph nodes of four, and in the liver of two kits but not in the brain, bone marrow, intestine, or spleen of any. The largest *Hepatozoon* meront in skin was 45 × 30 µm and contained 40 merozoites. The age of the affected kits indicates congenital transmission of *Hepatozoon*. *Encephalitozoon* sp. infection was confirmed cytologically and ultrastructurally, and might have compromised the host immunity.

The rationale for the new name, *H. mustelis*, included its occurrence in skin and in a new host, the polecat [56]. The parasite should be considered *nomen nudum*, because the findings were presented at a symposium, without any formal description of the parasite published in a peer-reviewed journal. No specimens were deposited in a museum or at any traceable location. Attempts to locate archived tissues in the laboratory at Laurel, Maryland, USA, where the work was performed, were unsuccessful (JPD own observations).

#### *Hepatozoon banethi* and *Hepatozoon ewingi*

*Hepatozoon banethi* was named by Greay et al. [57] based on DNA recovered from *Ixodes tasmani* from dogs in Australia, and *Hepatozoon ewingi* was named by the same authors based on DNA recovered from *Haemaphysalis bancrofti* from a horse in Australia. There was no morphological description of the parasites. The erection of these species was based solely on genetic distinctiveness. Harris [58] remarked that these species do not meet the required International Code of Zoological Nomenclature (ICZN) guideline, which states that new taxa should be accompanied by a morphological description. However, Greay et al. [59] argued that “ICZN does not explicitly rule out the use of DNA sequences alone.” We agree with Harris [58] on the invalidity of these species. The concept of species based solely on molecular characteristics could change in time as whole genome sequences become more available, but specific morphological descriptions are more sustainable and comprise an important part of naming a new protozoa species. When proposing new taxa, it is important to provide morphological descriptions incorporating light microscopy and electron microscopy if necessary, histopathology, and molecular characterization. As an argument, Dame et al. [60] synonymized *Sarcocystis neurona* with *Sarcocystis falcatula* based on similarities in the DNA sequences of the small subunit ribosomal RNA (rRNA) gene of both protozoa, despite morphological and biological differences [61]. Additionally, a finding of *Hepatozoon* in ticks removed from a host does not prove that the vertebrate host was infected with the parasite. In fact, if it is a tick that has had a blood meal on a previous host, it might have become infected from that past host. Thus, naming *H. banethi* and *H. ewingi* was not done according to the accepted standards and is unacceptable. In the opinion of one of us (JPD), Greay and associates’ [57] efforts to recognize these two scientists, Gad Baneth and Sydney Ewing, who made important contributions to hepatozoonosis, are praiseworthy, but these names are now shrouded in controversy, precluding the use of the names in the future for naming valid new *Hepatozoon* species.

## *Hepatozoon* infections in herbivores or omnivores

*Hepatozoon* species typically parasitize carnivores. However, there are rare reports in other hosts. *Hepatozoon* sp*.* gamonts were reported in blood smears of a giraffe (*Giraffa camelopardalis*) [62] and a reedbuck (*Redunca arundinum*) [63]; both animals were shot in Barotseland, South Africa. Basson et al. [64] described *Hepatozoon-*like structures in granulomas in histological sections of the livers of seven of 113 impala (*Aepyceros melampus*) from South Africa killed during the period 1965–1966. Single zoites measuring 15.0 × 2 0.5–3.0 µm with a centrally located nucleus were enclosed in vacuoles with foamy residual body or cytoplasmic mass. No meronts were found. Keep [65] confirmed these findings; he too found *Hepatozoon* sp. in granulomas in the liver of an impala from the Ndumo Game Reserve, South Africa.

*Hepatozoon* sp. meronts and gamonts were found in the myocardium of one of 500 white-tailed deer (*Odocoileus virginianus*) from Texas, USA [51]. Additionally, *H. canis* DNA was detected in the spleens of three out of 20 roe deer (*Capreolus capreolus*) from Austria, while *H. martis* was identified in two roe deer and in the spleen of one of 40 chamois (*Rupicapra rupicapra*) [66].

There is an unconfirmed report of *H. canis* DNA in the blood from one of 17 sheep from a farm in Tabasco, Mexico [67]. There is also a report of *Hepatozoon* sp. gamonts in the blood of a human. The patient was a 59-year-old carpenter and was among 300 patients screened for *Babesia* infection. The patient had fever, jaundice, and chills, probably related to duodenal diverticulum [68]. Gamonts were seen in both neutrophils and monocytes. No molecular evidence is available to support this description, and no human *Hepatozoon* infection has been described since.

Reports based on the recovery of *Hepatozoon* DNA from arthropod vectors from mammalian hosts are not discussed here. As stated earlier, the finding of *Hepatozoon* sp. in ticks removed from a host does not prove that the vertebrate host was infected with the parasite. In fact, if the tick has had a blood meal on a previous host, it might have become infected from that past host. Furthermore, just detecting the DNA of *Hepatozoon* in a tick with no morphological evidence of sporogonic development could stem from an infected blood meal, and the tick is not necessarily a vector or able to transmit this infection.

A species of *Hepatozoon, H. apri*, was reported from omnivorous wild boar *(Sus scrofa leucomystax*) in Japan [69]. The parasite was found in 96 of 181 (53.0%) wild boars hunted in Tokushima, Japan, and more recently, *H. apri* DNA was detected in the cardiac blood of 6 of 21 (28.5%) wild boars from Wakayama, Japan [70]. Previously, *Hepatozoon* sp. was found in the muscle of a wild boar from the Gifu Prefecture in Japan [71]. The gamonts were 9–16.5 × 4.2–11.5 µm and contained a small protrusion. Type 2 meronts were detected in the skeletal muscles and the myocardium and measured 35 × 26 µm. No lesions were described.

The following information supplements and updates the morphological description of *H. apri* by the authors [69] based on photos supplied by the authors and reappraisal of published images. Only a few meronts were found, and therefore descriptions are incomplete. The images are of type 2 meronts surrounded by a clear hyaline capsule. Image 3a in their paper, termed trophozoite, is probably part of a unizoite meront. Image 3b shows nuclei (not merozoites) arranged peripherally with indistinct foamy cytoplasm (a compact residual body was absent). The merozoites in Fig. 3c and d as illustrated in the original paper were arranged haphazardly [69]. The size of the merozoites is unknown.

Harris et al. [72] suggested that *H. apri* bore close resemblance phylogenetically to *H. felis* from the domestic cat. Earlier, Sumrandee et al. [73] expressed a similar opinion; *Hepatozoon* sp. DNA collected from the tick *Dermacentor astrosignatus* from a wild boar in Thailand was similar to *H. felis* DNA. However, *H. felis* is morphologically distinct from *H. apri* (Table 1).

## Hepatozoonosis in dogs

### *Hepatozoon canis* infections

#### Prevalence

*Hepatozoon canis* is one of the most common pathogens in dogs in many parts of the world, especially the tropics (Table 2). Most reports of canine hepatozoonosis are from India and Brazil (Tables 2, 3). The prevalence of *Hepatozoon* in the general canine population is largely unknown because most of the reports are on dogs that were taken to clinics for different disease conditions. A study from Argentina included a survey that was performed in domestic dogs [74]. Blood was obtained from the cephalic vein of 100,123 unselected domestic dogs by veterinary practitioners between October 2002 and May 2013 and tested at a central laboratory. Parasitemia was estimated by examining 100 microscopic fields (×1000 magnification) of thin blood smears stained with Giemsa. Parasitemia was expressed as gamonts per microliter and classified as low (< 100), mild (100–800), or high (> 800). Hematological parameters investigated were recorded [74]. *Hepatozoon canis* gamonts were found in 2328 of 100,123 (2.3%) dogs. Among the infected dogs, parasitemia was high in 29.2%, mild in 46.5%, and low in 18.6%. Anemia was the most common (56.9%) finding in infected dogs. As expected, infections were higher in summer than in winter, coinciding with arthropod activity. Co-infections with other protozoans were uncommon; *Babesia vogeli* was found in 14 and *Dirofilaria immitis* in seven of the 2328 *H. canis*-infected dogs [74]. These findings are useful for practitioners. Undoubtedly, the prevalence is an underestimation, because examination of blood smears is an insensitive diagnostic method for hepatozoonosis. Higher prevalence was reported by others who compared blood smear examination with polymerase chain reaction (PCR) detection (Table 2), [75–82]. It is worth noting that *H. canis* DNA was not detected in 578 rural dogs from Chile, but using the same methods, *Hepatozoon* DNA was detected in 48.3% of Andean foxes and 32.5% of gray foxes [83], suggesting a possible sylvatic cycle.

**Table 2:** Worldwide prevalence of *Hepatozoon canis* infections in domestic dogs (*Canis familiaris*) in chronological order of publication within each country

Country	Region/locality	Year sampled	Source^a^	No. of dogs positive/no. tested (%)	Diagnostic methods^b^	Remarks	References
Sub-Saharan Africa	Ghana, Kenya, Namibia, Nigeria, Tanzania, Uganda	NS	Urban, rural	352/601 (58.6)	PCR	*Hepatozoon canis* DNA in blood. Exact prevalence unknown, around 100 dogs from each country	[159]
Albania	Tirana	2008	Semi-domestic	5/30 (16.6)	C	Gamonts in blood	[160]
Algeria	NS	1910–1911	NS	2/356 (0.6)	C	Meronts in bone marrow of 1, spleen and bone marrow of another	[161]
Angola	Luanda	2013	VC	18/103 (17.5)	PCR	*H. canis* DNA. Concurrent infections with *Ehrlichia canis* and *Babesia*	[162]
Argentina	Buenos Aires	2005–2006	D	6/6 (100%)	C, PCR	**Longitudinal study of dogs in the same household for 2 years***. H. canis* gamonts in blood smears of 5. Parasitemia was inconsistent, and 1 dog remained aparasitemic. *H. canis* DNA in blood of all 6 dogs	[163]
Argentina	Santa Fe	2008–2010	VC	9/490 (18.3)	C	Gamonts in blood smears. Infected dogs 2–6 months old, anorexia, weakness, and mild anemia	[164]
Argentina	San Luis	2014–2015	D	6/61 (9.8)	C	Anorexia, anemia lymphadenopathy, dermatitis in infected dogs. Thesis abstract	[165]
Argentina	Buenos Aires	2002–2013	VC	2328**/100,123** (2.3)	C	**Gamonts in blood. Among infected dogs, anemia 56.9%, neutrophilia 74.1% (see text)**	[74]
Argentina	La Rioja	2016–2017	VC	11/108 (10.1)	C	Anemia, leukocytosis, and thrombocytopenia in infected dogs	[166]
Argentina	Buenos Aires	2018–2019	D	15/207 (7.2)	C	*H. canis* gamonts in **peripheral whole blood smears of 10 and in buffy coat of 15 dogs.** Prevalence varied with the parts of the city sampled	[167]
Brazil	Rio de Janeiro	1997–1998	Rural	98/250 (39.2)	C	Gamonts in blood smears. Prevalence data for 7 regions tabulated. Concurrent infections with *B. canis* and *E. canis.* One monocyte contained both *H. canis* and *E. canis*	[168]
Brazil	São Paulo	NS	NS	21/31 (67.7)	C, PCR	*H. canis* gamonts in blood of 7 and DNA in all 21	[76]
Brazil	São Paulo	2000–2001	ST	13/222 (5.9)	C	Gamonts in blood. No hematological abnormalities in 9 of 13 parasitemic dogs. Co-infections *with B. canis* and *E. canis*. Five dogs were necropsied. Tissue stages (**mostly unizoite tissue cyst-like structures**) were found in visceral tissues of 2 dogs, but not in muscles	[169, 170]
Brazil	Brasília	NS	D	22/30 (66.6)	C	Gamonts in blood. Hematological data discussed	[171]
Brazil	Rio de Janeiro	2004	Urban	1/12 (8.3)	C, PCR	Gamonts and DNA in blood	[172]
Brazil	São Paulo	NS	Rural, from 47 houses	80/150 (53.3)	C, PCR	Gamonts in blood smears of 17; **7 from ear vein, 14 from cephalic vein, and 4 from both.** *H. canis* DNA in blood of 80	[173]
Brazil	Goiás	2007	SE	18/53 (33.9)	C	Gamonts in blood smears. Co-infections with other blood pathogens	[174]
Brazil	Espírito Santo	2007–2008	Healthy dogs, rural and urban areas	54/92 (58.7)	PCR	20 *H. canis*-infected dogs were co-infected with *Babesia*	[175]
Brazil	Pernambuco (Recife)	2007–2008	VC	1/205 (0.5)	PCR	49 (23.9%) of 205 dogs were co-infected with 2 or more pathogens	[176]
Brazil	Minas Gerais	2008–2009	VC 120, D 80, SE 100 (total 300)	23/300 (7.6)	C	Gamonts in 23 dogs (6-VC, 1 D, 16 SE), 8 with concurrent infections with *B. canis* and/or *E. canis.* Pain on renal palpation, anemia, lymphadenopathy	[177]
Brazil	Minas Gerais (Uberlândia)	NS	Farm dogs	4/7 (57.1)	C, PCR	Gamonts in blood smear. ***H. canis* sporulated oocysts recovered from *Rhipicephalus microplus* from a dog**	[178]
Brazil	Minas Gerais (Uberlândia)	NS	Rural and urban, door to door survey	274/346 (79.2)	PCR	161 of 212 (75.9%) urban and 113 of 134 (84.3%) rural dogs infected	[179]
Brazil	Mato Grosso do Sul	2007–2009	VC	6/165 (3.6)	PCR	*H. canis* sequences	[180]
Brazil	Pará	2011	VC	7/138 (5.0)	PCR	*H. canis* DNA in blood; sequences deposited	[181]
Brazil	Mato Grosso do Sul	2009–2010	VC	9/2,505 (0.3)	C	Gamonts in blood smears. Anemia in 6 of 9 infected dogs. Hematology data for all 9 dogs tabulated. Co-infection with *B. canis* in 2 dogs	[182]
Brazil	Goiás	NS	VC	2/40 (5.0)	C, PCR	*H. canis* DNA and gamonts in blood of both. DNA sequences deposited	[183]
Brazil	Paraíba	NS	VC, urban	14/151 (9.3)	C	*H. canis* gamonts in blood from ear tips. Clinical signs and hematological data listed for infected dogs	[184]
Brazil	Mato Grosso do Sul	2013–2015	ST	19/42 (45.2)	C, PCR	No gamonts in blood smears. *H. canis* DNA in blood. Co-infection with other pathogens	[185]
Brazil	Rio Grande do Norte	2012–2013	SE	2/20 (10.0)	PCR	*H. canis* DNA from spleens	[186]
Brazil	Paraíba	2017	Rural	8/98 (8.1)	C	Gamonts in blood	[187]
Brazil	Brasília	2020–2022	VC	21/72 (29.1)	C, PCR	*H. canis* gamonts in 7, DNA in 21	[94]
Brazil	Mato Grosso do Sul	2018–2023	Semi-domesticated	3/28 (10.7)	PCR		[188]
Cambodia	Preah Vihear province	NS	Semi-domesticated	11/101 (10.9)	PCR	*H. canis* DNA in blood. Concurrent infections	[189]
Cape Verde Archipelago, West Africa	Praia	2008	VC	83/130 (63.8)	PCR	*H. canis* DNA in blood; sequences deposited. Concurrent infections with other blood pathogens	[190]
Cape Verde Archipelago, West Africa	Maio island	2012	D	55/153 (35.9)	PCR	*H. canis* DNA in blood, sequences. Co-infections with other blood pathogens	[191]
Chad (Africa)	NS	2019	D	346/428 (80.8)	PCR	*H. canis* DNA in blood	[192]
Chile	Several areas	2015–2019	Rural	0/578 (0)	PCR	Whole blood or spleen	[83]
China	Hong Kong	2009–2010	D, ST	3/200 (1.5)	PCR	*H. canis* DNA in blood of 2 of 100 stray dogs and 1 of 100 pets	[193]
China	Jiangsu	2012–2014	VC	20/1114 (1.8)	PCR	Co-infections with other blood pathogens	[194]
China	Beijing, Nanjing, Shanghai, Guangxi Province	2017–2018	NS	84/481(1.6)	PCR		[195]
China	Xi’an, Hanzhong	2017–2018	VC	4/196 (2.0)	PCR	Zero of 104 dogs from Xi’an, 4 of 92 from Hanzhong	[196]
China	Chongqing, Hubei, Fujian, Shandong	NS	VC	10/306 (3.2)	PCR		[197]
China	Hainan	2017–2022	NS	135/1039 (13.0)	PCR		[198]
Colombia	Bogotá, Bucaramanga, Villavicencio	NS	SE	29/91 (31.8)	PCR	*H. canis* gamonts in blood smears of 4 (1 dog was PCR-negative) and DNA in 25	[199]
Colombia	Cúcuta	2016	D	30/350 (8.6)	PCR		[200]
Colombia	Magdalena	2017	VC	12/169 (7.1)	PCR	Seven *H. canis*-infected dogs were co-infected with *B. canis.* DNA sequences deposited	[201]
Colombia	Caldas, La Estrella, Sabaneta	2017–2018	SE	31/357 (8.7)	PCR	*H. canis* DNA from blood	[202]
Colombia	Pereira, Risaralda	2020	VC	28/100 (28.0)	C, PCR	Survey for blood pathogens; 85 dogs ill, 15 healthy	[203]
Colombia	Tolima	NS	Urban, rural	14/308 (4.5)	PCR	All positive samples sequenced and revealed *H. canis*	[204]
Costa Rica	Four regions	2012	D	11/146 (7.5)	PCR	Co-infections with other pathogens	[205]
Croatia	Several areas	2007–2008	AS	108/924 (11.8)	C, PCR	*H. canis*: 5 genotypes. Of 14 asymptomatic dogs followed hematologically, 9 dogs had altered hematological values and serum enzymes—they were tabulated for each dog [207]	[206, 207]
Cuba	Several areas	2016–2017	ST	38/80 (47.5)	C, PCR	*H. canis* gamonts in blood smears of 8 and DNA in blood of 38	[77]
Czech Republic	Three regions	2014–2015	Hunting dogs	4/8 (50.0)	PCR	Sequences deposited	[208]
Czech Republic	South Moravia, South Bohemia, Prague	NS	D, Hunting dogs, SE	16/418 (4.0)	PCR	Sequences deposited	[209]
Egypt	NS	2022	VC	30/100 (30.0)	C	Gamonts in blood. Anemia and elevated serum enzymes	[210]
Egypt		2000–2001	Police dogs	15 /300 (5.0)	C, HE, PCR	Dogs were sick. *H. canis* gamonts and DNA in blood of 15. Tissues of 15 dead dogs studied histologically. Lesions and type 2 meronts in spleen and liver of at least 1 dog; the number not stated	[211]
France	Several regions	2006–2007	VC	1/108 (0.9)	PCR	*H. canis* DNA	[212]
Gabon (Central Africa)	Northeast	NS	NS	30/255 (11.7)	NS		[213]
Galapagos	Four islands	2021–2022	D	2/1221 (0.1)	PCR	*H. canis* sequences	[214]
Germany	Imported or traveling dogs	2004–2009	D	203/**5012** (4.0)	C, PCR	*H. canis* gamonts in blood smears of 133 of 4681 dogs imported from various countries*.* Additionally*, H. canis* DNA was found in 70 of 331 dogs imported from Portugal	[215]
Ghana	Kumasi	2010–2011	VC	4/10 (16.0)	PCR	*H. canis* DNA in blood. 1 dog co-infected with *Ehrlichia*, 1 with *Anaplasma* sp.	[216]
Greece	Thessaloniki	1998–2001	VC	2/69 (2.9)	C	**Gamonts in blood smears of both. Fever, anemia, lymphadenopathy, and splenomegaly in both dogs**	[217]
Grenada	Several areas	2006	VC	5/73 (6.8)	PCR	*H. canis* in 4 of 60 dogs from St. George, 1 of 9 St. David. Survey for vector-borne pathogens	[218]
Haiti	Throughout	2013	VC	40/207 (19.3)	PCR	**No *H. americanum;*** co-infections with other vector-borne pathogens	[219]
Hungary	South, 24 locations	2012	100 shepherd, 12 hunting, 14 stray	33/126 (26.1)	PCR	*H. canis* DNA in blood. 31% of shepherd dogs, 8% of hunting dogs, and 7% of stray dogs infected	[220]
India	Assam, Bengal	1904–1905	D	6/45 (13.3)	C	Gamonts in blood smears	[26]
India	Haryana (Hisar)	NS	D, ST	2/88 (2.7) D, 13/476 (2.7) ST, 0/15 (0) police dogs	C	Gamonts in blood smears. 26 dogs (5.4%) had *Babesia*	[221]
India	Andhra Pradesh	1978–1983	D	4/50 (2.0)	C	Survey for blood protozoa	[222]
India	Tamil Nadu (Chennai)	NS	VC	27/809 (3.3)	C	Dogs tested for *E. canis;* 4 dogs co-infected with *H. canis* and 23 dogs had *H. canis* alone	[223]
India	Tamil Nadu (Chennai)	2007	VC	23/**4190** (0.5)	C	Gamonts in blood smears of ear tips	[224]
India	Punjab (Ludhiana)	2007–2009	VC	4/488 (0.8)	C	Gamonts in blood. Co-infections with other blood pathogens	[225]
India	Four regions	2008	ST, SE	12/525 (2.3) gamonts; DNA 0–43.9%	C, PCR	*Hepatozoon* DNA in 38.3% of 161 from Delhi, 43.9% of 162 from Mumbai, 0% of 101 from Sikkim, and 24% of 100 from Ladakh**. First comparative prevalence from dogs in 4 different climates**	[226]
India	Punjab (Ludhiana)	2010	VC	5/460 (1.0)	C	Gamonts in blood	[227]
India	Telangana (Hyderabad)	NS	VC	2/3 (66.6)	PCR	*H. canis* DNA	[228]
India	Assam	2009–2010	ST, VC, working dogs	7/468 (1.5)	C	*H. canis* gamonts in blood. Co-infections with other pathogens	[229]
India	West Bengal (Kolkata)	2011–2012	VC	3/47 (6.3)	C	*H. canis* gamonts in blood. All 47 dogs were infected with *E. canis*	[230]
India	Tamil Nadu (Chennai)	2006–2011	VC	**223/14,992 (1.5)**	C	Gamonts in blood smears. *Babesia* co-infections	[84]
India	Odisha (Bhubaneshwar)	2011–2012	VC	18/541 (3.3)	C	Gamonts in blood smears. Co-infections with other blood pathogens	[231]
India	Madhya Pradesh (Jabalpur)	2012–2013	VC	2/1680 (0.2)	C	Gamonts in blood	[232]
India	Punjab (Ludhiana)	2014	VC	2/778 (0.2)	C	Gamonts in blood	[233]
India	Punjab (Ludhiana)	2015–2016	VC	31/225 (13.7)	C, PCR	*Hepatozoon* gamonts in blood of 13 and DNA in 31	[234]
India	Tamil Nadu (Namakkal)	2015–2016	VC	8/150 (5.3)	C, PCR	Gamonts in 1 blood and DNA in 8	[79]
India	Andhra Pradesh	2014–2018	VC	9/1350 (0.6)	C	Gamonts in blood. Co-infections with other blood pathogens	[235]
India	Kerala (Mannuthy)	2017–2018	VC	6/150 (4.0)	C, PCR	*H. canis* gamonts and DNA in blood of 6	[236]
India	Northeast (Aizawl)	2017–2018	D, ST	8/525 (1.5)	C	Survey for tick-borne pathogens. Gamonts in blood	[237]
India	Tamil Nadu (Chennai)	2018	VC	87/230 (37.8)	PCR	Dogs tested for canine vector-borne diseases	[238]
India	Maharashtra (Mumbai)	2015–2017	VC	3/127 (2.3)	C	*H. canis* gamonts in blood of 3. The 127 dogs were tested because of anemia	[239]
India	Tamil Nadu (Chennai)	2019–2021	VC	32/482 (1.0)	C, PCR	Gamonts in blood smear of 5	[240]
India	Tamil Nadu (Tirunelveli)	2020	VC	7/275 (2.5)	C	Gamonts in blood. **Chippiparai dogs (sighthound)**	[241]
India	Tamil Nadu (Chennai)	2010–2019	VC	399/**11,000** (3.6)	C	Retrospective study. Gamonts in blood smears	[242]
India	Tamil Nadu (Chennai)	2024	VC	7/**6415** (0.1)	C	Gamonts in blood smears. Co-infections with other blood parasites	[243]
India	Haryana (Hisar)	2021–2022	VC	42/120 (35.0)	C, PCR	Canine tick-borne diseases survey. *H. canis* gamonts in blood smear of 7, DNA in 42. Co-infections with other pathogens	[244]
India	Andhra Pradesh (Tirupati)	2021	VC	11/97 (11.3)	C, PCR	Gamonts in blood smears of 2 *H. canis* DNA in 9	[75]
Iran	Mashhad	2010–2011	ST, VC	4/254 (1.5)	C	Gamonts in blood	[245]
Iran	Ardabil	2013–2015	D	24/104 (23.0)	PCR	*H. canis* DNA in blood	[246]
Iran	Torbat Heydarieh	2014–2015	ST	12/150 (8.0)	C, PCR	*H. canis* gamonts in blood of 5 (3.3%), and *H. canis* DNA in 12 (8.0%)	[78]
Iran	Tehran	2015–2016	D, SE, ST	32/145 (22.0)	C, PCR	No gamonts in blood smears	[247]
Iran	Urmia	2018–2019	D, SE, ST	23/246 (9.3)	C, PCR	*H. canis* gamonts in blood smears of 5 and DNA in 23. Prevalence: 16 of 103 shelter (15.5%), 5/99 (5.0) in stray, and 2 of 44 (4.5%) in pets	[248]
Iran	Shiraz	2020–2021	VC	8/108 (7.4)	C, PCR	Gamonts in blood smear of 1. *H. canis* DNA in 8	[249]
Iraq	Bagdad	1910	ST	110/110 (100.0)	C	**Spleen or bone marrow. Probably first description of** *H. canis* **meronts in tissues of dogs**	[250]
Israel	Bet Dagan	1968–1973	Dogs necropsied	9/688 (13.3)	C, HE	***Hepatozoon* was considered the cause of death in 4 dogs that died and meronts were found in tissues**. Gamonts in blood smear of 4, impression smears of organs of 2, and meronts in histological sections of tissues of 4	[251]
Israel	Several areas	NS	161 SE, 125 D	3/286 (1.2)	C, S	Gamonts in 3 of 286; IFA antibodies to gamonts in 53 (33.1%) of 160 dogs from shelter	[98]
Israel	NS	NS	D	7 littermates	C	Anemia. Parvoviral co-infection. Gamonts	[252]
Italy	Central and Northeastern	2005–2006	AS	14/385 (3.6)	C, PCR	*H. canis* gamonts in 2 of 416, and DNA in 14 of 385	[80]
Italy	South	2009	83 (73 mongrel, 10 beagles)	49/83 (59.0)	C, PCR	**Gamonts in 10.8% of blood, 41.5% of buffy coat, and 16.3% in bone marrow. By PCR, DNA in 50.6% of blood, 51.2% of buffy coat, 48.1% of bone marrow, and 27.7% of skin**	[81]
Italy	Campania	2015	Hunting dogs	200//1433 (13.9)	PCR	*H. canis* DNA in blood of healthy dogs*.* 36 co-infected with *Ehrlichia* [254], 94 had *Mycoplasma* [253]	[253, 254]
Italy	Sardinia	2020–2021	VC	9/51 (17.6)	PCR	*H. canis* DNA. Muscular pain in 4, fever in 1, lymphadenopathy in 1, 3 asymptomatic	[255]
Ivory Coast (West Africa)	Abidjan	NS	NS	12/133 (9.0)	NS		[213]
Japan	Yamaguchi	1994–1998	VC	18 /430 (4.2)	S	*Hepatozoon* IFA antibodies. Data on dogs tabulated. Co-infections with *E. canis*	[99]
Japan	Five prefectures	2011	D	84/196 (42.9)	C, PCR	*H. canis* gamonts in 45/191 (23.6%)-gamonts in blood, and DNA in blood of 84 of 196 (42.9%)	[82]
Jordan	Several areas	2006	Dead, ST	11/38 (28.9)	PCR	DNA from heart blood. Co-infection with *Anaplasma phagocytophilum* and *Babesia caballi*	[256]
Kosovo	7 districts	2021–2022	D, SE, ST	52/276 (18.8)	PCR	Co-infections with other pathogens. Survey for *Leishmania*	[257]
Kyrgyzstan	Bishkek	2016–2017	AS	49/170 (28.8)	PCR	DNA extracted from whole blood	[258]
Malaysia	Kuala Lumpur	1973–1981	D	48/**4084** (1.2)	C	Gamonts in blood. Survey for other blood parasites	[259]
Malaysia	Kuala Lumpur	NS	VC, SE	8/240 (3.3)	PCR	*H. canis* DNA in 8 SE dog blood; none from 189 VC dogs	[260]
Malaysia	Klang Valley, Kota Bharu	2017–2018	D	2/45 (4.4)	PCR	*H. canis* DNA	[195]
Malta	Malta, Gozo	2013	D	16/99 (16.0)	PCR	*H. canis* DNA in blood of 16, concurrent *Anaplasma platys* infection in 3	[261]
Mauritius	Port Louis	2014	SE	9/78 (11.5)	C	*H. canis* gamonts in blood. Concurrent infections with other pathogens in 7 of 9 *Hepatozoon* infected dogs	[262]
Mauritius	Four locations	2015	D	3/15 (20.0)	PCR	*H. canis* DNA in blood*.* sequences deposited	[263]
Mexico	Tabasco	2013–2014	NS	19/30 (63.3)	PCR	*H. canis* DNA in blood	[5]
Nicaragua	Rivas	2012	AS	20/39 (51.2)	PCR	Concurrent infections	[264]
Nigeria	Zaria	1970–1972	VC	71/335 (22.1)	C	Gamonts in blood. *H. canis* alone in 55, co-infections in 16 with *E. canis* and *B. canis*	[265]
Nigeria	Zaria	1975–1977	VC	74/**4452** (0.2)	C	Survey for blood protozoa. *H. canis* gamonts in blood smears of 74. ID medication reduced parasitemia. This is an abstract without any details	[266]
Nigeria	Zaria	1977–1979	VC	**1069/6054 (**17.6)	C	Concurrent infections with *B. canis, E. canis*. **Medication with ID (5 mg/kg, once subcutaneously) reported 98% recovery**	[267]
Nigeria	Zaria	1981	VC	78/354 (22.0)	C	Gamonts in blood. Four cases were clinical (see Table 3)	[268]
Nigeria	Several areas	2004–2005	NS	81/400 (20.3)	PCR	*B. canis* survey. *H. canis* DNA in blood	[269]
Nigeria	Plateau, Kaduna, Kwara, Rivers	2011	VC	75/181 (41.4)	PCR	*H. canis* DNA in blood. Co-infections with other pathogens	[270]
Pakistan	Punjab	2014–2015	D	18/151 (11.9)	PCR	Four *H. canis* sequences deposited. Lower hematocrit values in infected dogs versus non-infected dogs	[271]
Pakistan	Punjab (Kasur, Rawalpindi, Muzaffargarh)	2016	AS, farm dogs	155/341 (45.5)	PCR	*H. canis* DNA in blood; 30 PCR products from each of the 3 districts were sequenced. *H. canis* sequences formed 3 clades	[272]
Palestine	West Bank (10 districts)	2010–2015	AS, rural	20/362 (5.5)	PCR	*H. canis* DNA in blood. Concurrent infections with *Babesia* spp.	[273]
Philippines	Manilla	NS	Several cities	1/168 (0.05)	C, PCR	*H. canis* gamonts and DNA in blood. Survey for hemoparasites	[274]
Philippines	Cabanatuan, San Jose, Muñoz	2017–2018	D	22/120 (18.3)	PCR	*H. canis* DNA on 1 blood sample	[195]
Poland	Mazovia	2020–2022	D, VC	11/47 (23.4)	C, PCR	Five cases were clinical and had concurrent infections (see Table 3). Gamonts in blood of 7, 11 PCR-positive	[275]
Poland	Lublin Voivodeship	2023	VC	3/107 (2.8)	PCR	*H. canis* DNA in blood; sequences deposited. The 3 *H. canis* infected dogs had apathy, anorexia, and muscle weakness	[276]
Portugal	Alijó Municipality	NS	VC	45/124 (36.2)	C	Gamonts in blood	[277]
Portugal	Northern part	2007–2009	D	1/45 (2.2)	PCR	*H. canis* DNA blood sample; all dogs were infected with *Babesia* sp.	[278]
Portugal	Exported to Germany	2004–2009	D	70/331 (21.2)	C, PCR	*H. canis* gamonts in buffy coat smears of 62 dogs; concurrent heartworm microfilariae	[215]
Qatar	Doha city	2016	VC	1/64 (1.6)	PCR	*H. canis* DNA in blood	[279]
Romania	Snagov (Ilfov county)	2013–2014	VC	14/96 (14.5)	PCR	Three *H. canis* genotypes*.* 6 dogs co-infected with* Mycoplasma*	[280]
Romania	South	2017	SE	143/300 (47.6)	PCR	*H. canis* DNA in blood from cephalic vein. Survey for piroplasms. 9 dogs co-infected with *Babesia* spp.	[146]
Russia	Sochi	NS		9/100 (9.0)	PCR	*H. canis* alone in 5, concurrent pathogens in 5	[281]
Singapore	NS	NS	NS	3/36 (8.3)	C	Gamonts in blood smears	[282]
Slovakia	39 districts	2007–2015	AS, D, SE	3/293 (1.0)	PCR	Retrospective study	[283]
South Africa	Pretoria	NS	NS	1/1 (100)	C	*H. canis* gamont and *E. canis* in the same neutrophil	[284]
Spain	NS	NS	VC	4/15 (26.6)	PCR	Dogs were suffering from different conditions	[145]
Spain	Murcia	2010–2016	ST	11/82 (13.4)	PCR	*H. canis* DNA in blood or tissues; **no *H. felis***	[285]
Sri Lanka	Colombo	NS	SE	3/32 (9.3)	C	Gamonts in blood smears	[286]
Sri Lanka	Nationwide	NS	Several sources	1/668 (0.1)	PCR	*H. canis* sequences similar to those from India. Survey for blood pathogens	[287]
St. Kitts, West Indies	Ross Univ. clinic	2009–2011	VC	5/165 (3.0)	PCR	*H. canis* alone in 3 and co-infection with *E. canis* in 2	[288]
St. Kitts, West Indies	Ross Univ. clinic	2009–2011	VC	15/266 (5.6)	PCR	*H. canis* DNA in blood. Concurrent infections with other blood pathogens	[289]
Sudan	Eastern	1997–2000	ST	33/78 (42.3)	PCR		[290]
Tanzania (previously Tanganyika)	Ujiji	1910	NS	1/1 (100)	C	Gamont in blood leucocytes. This is a one-line note on the last page of the paper	[291]
Thailand	Bangkok	2002–2003	Semi-domesticated at 3 monasteries	35/308 (11.4)	C, PCR	*H. canis* gamonts in blood in 8, DNA in 35	[115]
Thailand	Bangkok	NS	AS	6/20 (30.0)	PCR	*H. canis* genotypes characterized	[292]
Thailand	Maha Sarakham	2014	ST	8/79 (10.1)	PCR	*H. canis* DNA from blood from cephalic vein. Co-infections with *E. canis* and *B. canis*	[293]
Thailand	Nakhon Phanom	NS	D	21/43 (48.8)	C, PCR	*H. canis* gamonts in blood of 15 and DNA in 21; co-infections with *B. canis* and *E. canis*	[294]
Thailand	Buriram	NS	D	2/49 (4.08)	C, PCR	*H. canis* gamonts in blood smears of 1 and DNA in 2. Co-infection with other pathogens	[295]
Thailand	Bangkok, Chiang Mai	2017–2018	D	8/120 (6.6)	PCR	*H. canis* DNA	[195]
Thailand	Nakhon Pathom Province	2010–2011	VC	185/**8,700** (2.1)	C	Gamonts in buffy coat smears. **Plasma proteins correlated with intensity of gamonts**	[296]
Thailand	Mahidol University	2019–2023	VC	46/2,519 (18.2)	PCR	Dogs were sick. Retrospective study. Concurrent infections with *Ehrlichia* and *Babesia*	[297]
Turkey	NS	1933	D	1/1/ (100)	C	Gamont in blood smear	[298]
Turkey	Aegean region	2004	ST, VC	117/349 (36.8)	C, PCR, S	117 positive for IFA antibodies, 90 positive by PCR, 37 positive for gamonts in blood smears	[100]
Turkey	Diyarbakır	2011	AS, ST	10/63 (15.8)	C, PCR	No gamonts in blood smear. PCR positivity correlated with the presence of ticks	[299]
Turkey	Several areas	2010–2012	163-D 243-ST, 288 SE,	155/694 (22.3)	C, PCR	Gamonts in blood smears from 3 of 285 dogs and *H. canis* DNA in 155 of 694 dogs. Lower prevalence in pets (10.4%) than in shelter (20.7%) or stray (20.9) dogs. 41.2% of dogs were infested with *Rhipicephalus sanguineus*	[300]
Turkey	Konya, Karaman	2010–2013	AS	8/221 (3.6)	PCR	*H. canis* DNA in blood; 2 sequences deposited	[301]
Turkey	Diyarbakır Province	2015	SE	118/219 (54.3)	C, PCR	DNA in blood but no gamonts. Co-infections with *Babesia* sp.	[302]
Turkey	Ankara	NS	SE	51/103 (49.5)	C, PCR	*Hepatozoon* gamonts in blood of 4 and DNA in 51	[303]
Turkey	Sivas	NS	D	67/150 (44.6)	PCR	*H. canis* DNA in blood; sequences deposited from 2 dogs	[304]
Turkey	Aydın	2018–2019	VC	3/100 (3.0)	C, PCR	*Hepatozoon* DNA in 3, no gamonts	[305]
Turkey	Antalya	2022–2024	D, SE, VC	27/120 (22.5)	C, PCR	Gamonts in blood of 3. Co-infection with *Babesia*	[306]
Ukraine	Kiev	2011	VC	1/23 (4.3)	PCR	*H. canis* DNA in blood	[307]
Ukraine	Translocated to Poland after Russian invasion	2022	D-2, ST-51	27/53 (50.9)	PCR	*H. canis* DNA in blood, sequences deposited. Co-infection with *Dirofilaria*	[308]
Uruguay	Several areas	2015–2019	VC	30/803 (3.7)	PCR	Two clades of *H. canis.* Co-infection with *Rangelia vitalii* and *Ehrlichia*	[309]
USA	California	2011	Greyhound kennel	1/55 (1.8)	PCR	***H. felis*-like DNA in blood**	[310]
Venezuela	Falcón State	NS	D	60/134 (44.7)	C, PCR	Gamonts in blood of 18, *H. canis* DNA in blood of 60. Three *H. canis* genotypes	[292]
Vietnam (previously Tonkin)	NS	NS	NS	1 dog	C	Gamont in blood smear	[311]
Vietnam	Hanoi, Ho Chi Minh City	2018–2018	D	2/120 (2.6)	PCR	*H. canis* DNA	[195]
Zambia	National parks	2009–2011	D	2/16 (12.5)	PCR	DNA from blood	[129]

*NS* not stated ,*B.canis =Babesia canis*, *Ehrlichia canis =E. canis*, *Rhipicephalus sanguineus=R. sanguineus*

Information in bold = important

^a^*AS* asymptomatic general population, *D* domestic, *SA* survey for arthropods, *SE* shelter, *SP* survey for other pathogens, *ST* stray, *VC* admitted to veterinary clinic

^b^*C* cytology, blood smears, *IFA* indirect fluorescent antibodies, *PCR* *18S* rRNA gene, *S* serology

**Table 3:** Clinical *Hepatozoon canis* infections in domestic dogs (*Canis familiaris*), arranged in chronological year of publication within each country

Country	Locality	Year	History and clinical data	Findings and remarks	References
Argentina	Buenos Aires	1998	A 3-year-old male	Anorexia, nasal bleeding. Severe anemia. Leukocytosis, hyperglobulinemia. Gamonts in blood smear	[312]
Argentina	Gran Buenos Aires	1999	Two cases. Dog 1: a 5-month-old Siberian husky Dog 2: a 4-month-old male Pekingese	Dog 1: Pain, difficulty walking with rear limbs, anorexia, atrophy of rear limbs. Severe anemia (Hb 5.8 g/dl). Dog died, necropsied. **Periostitis with new bone formation**. *Hepatozoon* meronts in tissues Dog 2: Lethargy, inability to walk Gamonts in blood smears of both dogs	[313]
Argentina	San Andrés de Giles	NS	Kennel outbreak	An outbreak of hepatozoonosis in a shelter dog kennel. One dog died of distemper virus infection and *H. canis*. Subsequent examination revealed *H. canis* gamonts in blood smears of 13 of 91 dogs. Clinical signs in *H. canis*-positive dogs were anemia, weight loss, fever, anorexia, and muscle weakness. Medication with toltrazuril (14 mg/kg, daily for 14 days) cured parasitemia and led to clinical improvement	[314]
Argentina	San Luis city	2009	A 2-year-old male dog	Anorexia, fever, dehydration, cachexia, severe anemia, leukocytosis, hypoproteinemia. *H. canis* gamonts in blood. Successfully treated with toltrazuril	[315]
Argentina	Buenos Aires	2007–2008	50 dogs	Gamonts in blood. 42 of 50 dogs had clinical signs: anemia in 26, weight loss in 17, anorexia in 14, weakness in 13, depression in 9; fever and muscle pain in 5; **periostitis** in 2; lymphadenopathy in 4; neurological symptoms in 2; hepatomegaly in 1; 8 dogs were asymptomatic	[316]
Argentina	Mendoza	2012	A 12-year-old dog	Lethargy, cachexia, hypothermia, weakness. Gamonts in blood smears. Hematological data	[317]
Argentina	Entre Ríos	NS	Two cases. Dog 1, a 3-month-old male mixed breed Dog 2, a 3-year-old golden retriever	Depression, loss of appetite, diarrhea in both dogs. *H. canis* gamonts in blood smears of both dogs	[318]
Argentina	La Pampa	2014	Four dogs: 1 greyhound mix, 3 unknown breeds, adults	Weight loss, lethargy, muscle atrophy, light normocytic/normochromic/regenerative anemia in 1. Alopecia, skin redness, hyperkeratosis, general lymph adenomegaly, and co-infection with *Demodex canis* in 1. 2 dogs asymptomatic. Gamonts in blood smears of all 4 dogs	[319]
Argentina	Mendoza	2019	Two cases. Dog 1, 12-year-old female mixed breed; dog 2, 3-month-old mixed breed	*H. canis* gamonts in blood smears of both dogs and anemia. Hematological and blood chemistry data tabulated. Dog 1 had hypothermia, lethargy, poor health Dog 2 was lethargic, loss of appetite, pain and difficulty standing, hypothermia	[320, 321]
Argentina	Mendoza	2017–2019	Seven dogs; 3 months to 7 years old	Lethargy, pale mucous membranes, anemia. *H. canis*, gamonts and DNA in blood	[322]
Argentina	Buenos Aires	NS	A 10-year-old mixed-breed Collie	Anorexia, lethargy, and chronic cutaneous lesions. Disseminated protothecosis diagnosed. *H. canis* gamonts and *Ehrlichia canis* morulae in blood smears	[323]
Australia	NS	2017	A 3-month-old Maremma sheepdog	Gamont in blood along with *Dirofilaria* larva. Diagnosis confirmed by PCR. Blood chemistry data, elevated enzymes. *H. canis* sequence deposited. First case of canine hepatozoonosis in Australia	[324]
Brazil	Minas Gerais (Uberlândia)	1991	Two cases	Gamonts in blood smears. Hyperthermia, hyperemic ocular mucous membranes, lung rales in both animals. Prostration, apathy, cachexia, anemia, seromucous ocular and nasal discharge. Concurrent infection with *E. canis*. First report of *H. canis* in Brazil	[325]
Brazil	Minas Gerais (Uberlândia)	1991–1994	22 cases, retrospective	Fever in 19, anorexia in 15, diarrhea in 12, naso-ocular discharge in 11. Gamonts in blood; concomitant infections in 18; *Ehrlichia* sp. 8, helminths 11, *Toxoplasma gondii* 1	[326]
Brazil	São Paulo state	1993–1994	Eight cases, 2 months to 11 years old	Anorexia, fever, weight loss, pain. *H. canis* gamonts in blood smears. Data tabulated for all dogs. Concurrent infections with other pathogens	[327]
Brazil	Brasília	1999–2001	Three cases: one 3-month-old Brazilian mastiff, one 18-month-old mixed breed, one 9-year-old German shepherd	Anorexia, weight loss, anemia. Gamonts in blood. Meronts in histological sections of biopsy of unlisted visceral organs. *H. canis* DNA	[328]
Brazil	Brasília	NS	3 (two 9-year-old mixed-breed females, 1 male 10-year-old Collie)	Anorexia, weight loss, anemia. Gamonts in blood smears. **Biopsy of muscle revealed myonecrosis and myositis but no parasites**. Medication with decoquinate for 15 months did not eliminate parasitemia	[329]
Brazil	Minas Gerais	1995–2005	115 cases, retrospective	Anorexia and loss of appetite in 56, hypothermia in 49, gamonts in blood of all; concomitant infections in 26	[330]
Brazil	Rio Grande do Sul	NS	A 4-year-old Collie	Progressive weight loss. Ear tip bleeding associated with *Rangelia vitalii*. Gamonts in blood, confirmed by PCR	[331]
Brazil	Mato Grosso do Sul	2009	10 dogs, 1 month to 7 years old	*H. canis* gamonts in blood smears and confirmed by PCR. Anorexia, lymphadenopathy, vomiting, polydipsia, fever, anemia listed for each dog	[332]
Brazil	Mato Grosso do Sul	2013–2014	48 cases selected in a veterinary clinic based on *H. canis* gamonts in blood. Information on breeds provided	Hematology compared infected versus uninfected. Anemia in 34 and thrombocytopenia in 26 of 48 infected dogs. Co-infections with other blood pathogens in 20.8%	[333]
Brazil	Goiás	2019	Five adult hunting dogs, mixed American foxhound	2 dogs = *Hepatozoon* sp. gamonts and DNA in blood—tachypnea, tachycardia, pale mucous membranes, abdominal pain, anemia, neutropenia, lymphocytosis and thrombocytopenia; 1 dog = *Hepatozoon* sp. gamonts and DNA in blood and *Babesia* sp.—pale mucous membranes, abdominal pain, anemia, thrombocytopenia, and hypoproteinemia; 1 dog = *Hepatozoon* sp. gamonts in blood smears and *Trypanosoma evansi* DNA—tachypnea, abdominal pain, anemia, and thrombocytopenia; 1 dog = *Hepatozoon* sp. gamonts and DNA in blood and *Babesia* sp. and *Ehrlichia* sp.—tachycardia, tachypnea, lymphadenomegaly, congested ocular mucosa, abdominal pain, anemia, leukopenia, neutropenia, and thrombocytopenia,	[334]
Brazil	São Paulo	2023	A 2-and-a-half-month- old golden retriever	Lethargy, anorexia, dysentery, hypothermia. *H. canis* gamonts in blood smear. Clinical improvement after medication with ID and DY	[335]
Brazil	Goiás	2018–2022	**272 dogs with confirmed *H. canis* infection**	272 dogs identified in 2018–2022. Most dogs were adults, with no sex or breed predisposition. Anemia, hyperproteinemia, thrombocytopenia predominated. Four co-infected with *Anaplasma platys*, 3 with *Babesia* spp., 14 with *Ehrlichia* spp. This study showed an exponential increase in cases diagnosed over the years, particularly in autumn and winter (the dry season)	[336]
Bulgaria	Stara Zagora	2001	A 7-year-old male Collie	Cachexia, weight loss, limb weakness and others, fever. Gamonts in blood smears. Hematology	[337]
Bulgaria	Stara Zagora	2007	A 7-year-old Alaskan malamute	Loss of appetite, weakness, muscle pain, ataxia, thrombocytopenia, elevated creatine kinase and alkaline phosphatase, co-infection *E. canis*. Died, necropsy performed. Meronts in liver lesions	[338]
China	Guangzhou	2015	3–4-month-old, male	The visual mucosa was pale, red blood cells decreased, elevated leukocytes, and eosinophils. Gamonts in blood smears. DY administered for 7 days, then died	[339]
China	NS	NS	A 15-year-old Tibetan lion female dog	Cachexia, fever, lymphadenitis, anemia, leukocytosis, serum creatinine, blood urea nitrogen and phosphorus elevated. Gamonts in blood smears, verified by PCR. Euthanized	[340]
Colombia	NS	2004	A 2–3-year-old pit bull male	Dermatitis. Gamonts in blood smears. Mild leukocytosis, elevated eosinophils	[341]
Colombia	NS	NS	Four cases: Case 1, 2-year-old Pomeranian male. Case 2, 5-year-old Rottweiler female. Case 3, 4-month-old female. Case 4, 3-month-old mixed breed	Gamonts in blood of all 4 dogs. Case 1, ulcers on tongue, heart murmur, cachexia. Case 2, depression, anemia, alopecia. Case 3, diarrhea, vomiting, anorexia. Case 4, loss of appetite, depression. Hematological values listed for all 4 dogs. Literature review and treatment discussed	[342]
Colombia	Medellín	NS	A 5-year-old male German shepherd	Vision loss, poor appetite. **Uveitis, corneal ulcer and glaucoma** *H. canis* gamonts in blood and in **aqueous humor.** Treatment with antibiotics, medicated eye drops, ID, and trimethoprim sulfa**.** Dog euthanized	[343]
Colombia	Cúcuta	2016	Two cases: Case 1, 18- month-old Schnauzer. Case 2, 19-month-old Siberian husky female	Case 1, anorexia, weight loss, vomiting. Anemia in both. Gamonts in blood smears of both	[344]
Colombia	Bogotá	NS	A 1-year-old mixed-breed female adopted from shelter	Pruritus, treated for kennel cough. *H. canis* DNA in blood by PCR. Hematological data tabulated. Clinical signs abated after medication with DY	[345]
Congo	NS	NS	One case: Fox terrier/native dog cross	Scabby patches, hair loss. Gamonts in leukocytes	[346]
Cyprus	Paphos	NS	Two adult mixed breed. Case 1: 1.5-year-old male. Case 2: a 7-year-old female	Anemia, weight loss. Both dogs had concurrent infections with *Ehrlichia* and *Babesia* or *Leishmania.* Gamonts in neutrophils in blood smears of both. DY treatment showed clinical improvement. The role of *Hepatozoon* in disease not clear because of other pathogens. First report of *Hepatozoon* infection in dogs in Cyprus	[347]
France	Sète (Hérault)	NS	A 7-year-old Brittany spaniel dog	Extreme weight loss, asthenia, diffuse alopecia. Gamonts in blood. Medication with penicillin–streptomycin–Phenergan and later Nivaquine–Rhodopraequine did not cure parasitemia; *H. canis* gamonts 3 months later in blood	[348]
France	Sommières	NS	28 cases	*H. canis* gamonts in blood smears. Details of ages, breed, clinical signs, hematological findings, and treatment with anti-protozoal therapy (Carbesia) tabulated for each dog	[349]
France	Sommières	NS	One case	A severe case of ehrlichiosis and hepatozoonosis*. H. canis* type 2 meronts in histological sections of liver and uninucleated zoites in impression smears of liver	[350]
France	Southern France	NS	Two cases: Case 1, a 4-month-old male Barzoi greyhound Case 2, a 7-year-old male German shepherd	Dog 1 with poor body condition, anemia, moderate fever, stiff while walking. Dog 2 with anorexia, fever, weakness *H. canis* gamonts in blood smears, with concurrent infections with *E. canis*	[351]
France	Southern France	1996	Two dogs	Gamonts in blood smears of both dogs. Lethargy, weight loss, pain in one dog with 22% parasitemia. Partial success with ID treatment. Severe relapse 8 days later, then successfully treated with toltrazuril (10 mg/kg, 10 days). Dog normal and gamonts disappeared at 6 months and 3 years. In the second dog, gamonts persisted at 14 days post-toltrazuril treatment but had disappeared at 38 days post-therapy. Clinical signs and treatment of canine hepatozoonosis reviewed	[352]
France	Southern France	NS	Three dogs: case 1, 9-year-old Beauceron; case 2, 11-year-old Beauceron bitch; case 3, a 10-year-old poodle	All 3 dogs had dermal lesions and *Hepatozoon* sp. gamonts in cytology smears of lesions. Case 1, enlarged adrenal glands and spleen and oral papillomatous. Therapy with ID and toltrazuril not successful. Case 2, pyoderma of limbs. Case 3, multiple dermal ulcers. Concurrent infection with *Leishmania*. Only abstract of the paper published	[353]
Germany	Munich	NS	A 3-month-old mixed-breed dog brought to Munich from a shelter in Rome	Fever, reluctance to move, emaciated, painful, stiff while walking. Gamonts in blood. Medicated with ID, DY, toltrazuril, sulfadoxine/trimethoprim. Parasitemia negative day 67	[354]
Germany	NS	NS	An 8-year-old male fox terrier and dam	Anorexia, weight loss, stiff gait, weakness ***Hepatozoon* in needle biopsy of lymph node detected microscopically. Diagnosis confirmed by PCR of blood and lymph node. Dam had gamonts in blood 2-years earlier but was still parasitemic**	[355]
Germany	NS	NS	An 11-month-old English bulldog imported from Hungary	Polyuria, polydipsia. Hematology, ultrasound, chronic renal insufficiency. Gamonts in blood. Medicated with ID, DY, sulfadiazine-trimethoprim, decoquinate cleared parasitemia	[356]
Germany	Ganderkesee	NS	A 4-year-old **pregnant** dog imported from Sardina, Italy	Lethargy, tachypnea. Blood PCR-positive for *H. canis* and *Rickettsia* sp. 8 pups born, 1 stillborn. The dam and the 7 pups were PCR-positive for *H. canis*, 62 days after birth. **Gamonts in buffy coats of blood of all pups and the dam**. **The stillborn puppy tissues were PCR-positive. Documentation of transplacental transmission of *H. canis***	[33]
Greece	North	1980–1987	11 (retrospective study)	Fever, depression, anorexia, loss of body weight Gamonts in blood. Data for each dog tabulated. Concurrent blood pathogens in 6	[357]
Greece	Thessaloniki	1998–2001	A 5-month-old fully vaccinated mongrel	Anorexia and depression for 2 days. Fever, lymphadenopathy Co-infection with *E. canis* and *Anaplasma phagocytophilum*. Gamont of *H. canis* and *E. canis* morula demonstrated together in a neutrophil. Dog medicated with ID and DY showed remarkable clinical recovery	[358]
India	Tamil Nadu (Madurai, previously called Madura)	NS	A 9-month-old Airedale terrier	Fever, inappetence. Gamonts in blood. Prolonged treatment with antimosan (a drug used to treat trypanosomiasis) was unsuccessful. Failed attempt to treat hepatozoonosis. First clinical hepatozoonosis from India	[359]
India	Tamil Nadu (Chennai, previously called Madras)	1937	Four mixed-breed dogs, 1–2 years old	Fever, inappetence. Gamonts in blood. Prolonged treatment with antimosan (a drug used to treat trypanosomiasis) unsuccessful	[360]
India	Tamil Nadu (Chennai, previously called Madras)	1942	Two dogs; case 1, a 6-month-old; case 2, cross-bred terrier	Numerous *H. canis* gamonts in blood smear of both dogs. Fever, inappetence, lumbar paralysis in dog 1. Attempts to treat dogs with arsenic compounds were unsuccessful	[361]
India	Haryana (Hissar)	NS	Seven dogs (no additional information)	Gamonts in blood. Chemotherapy trial. Medication with chloramphenicol, diminazene aceturate (Bernil), and lithium antimony thiomalate (Anthiomaline) were unsuccessful in curing parasitemia	[362]
India	Punjab (Ludhiana)	NS	A 2.5-year-old dog of unknown breed	Fever, epileptic fits, anorexia, vomiting, limb weakness. Gamonts in blood	[363]
India	Punjab (Ludhiana)	NS	Two dogs; case 1, a 7-year-old German shepherd; case 2, a 4-year-old male cross-breed	Dog 1: anorexia, fever. *H. canis* gamonts in blood smear. Co-infected with *E. canis*. Anemia. Treated with Terramycin for *E. canis* infection Fever, mild convulsions Dog 2: fever, dull, Gamonts in blood. Hematological values. Co-infection with *E. canis*	[364]
India	Maharashtra (Parbhani)	NS	A 3-month-old dog	Reduced appetite, fever, died after 5 days. Liver enlarged, necrosis in liver, spleen, mesenteric lymph nodes. Gamonts seen in blood smears and organ smears	[365]
India	Tamil Nadu (Chennai)	NS	A 1-year-old male dog	Tetraplegia, anorexia, lethargy. Anemia, gamonts in blood smear. **Gamonts in cerebrospinal fluid centrifuged sediment**. Recovery after treatment with trimethoprim-sulfadiazine	[366]
India	Punjab (Ludhiana)	1992	Two cases; case 1, a 7-year-old Pomeranian female; case 2, a 6-month-old female	Gamonts in blood smears of both dogs Dog 1, fever, vomiting, anorexia, dermatitis; attempted treatment with Terramycin, and anti-trypanosome drug Berenil Dog 2 had fever, fits, and died before treatment	[367]
India	Kerala (Thrissur)	NS	A 4-year-old German shepherd	Fever, edema of all limbs. **Radiography revealed periosteal reaction of cervical vertebrae.** Slightly elevated alkaline phosphatase. Gamonts in blood. Gamonts were not found after medication with sulfa-trimethoprim (15 mg/kg) and clindamycin phosphate (5 mg/kg, intramuscular)	[368]
India	Maharashtra (Mumbai)	NS	14 cases	Gamonts in blood smears of all. Hematological and biochemical changes detailed. Anemia in 13 dogs. All 14 dogs had elevated levels of liver enzymes. Hematological values listed	[369]
India	Maharashtra (Mumbai)	2010	Two cases. Case 1, 1-year-old male; case 2, 1.5 years old	Anorexia, weight loss, fever in both dogs. Gamonts in blood, neutrophilic leukocytosis. Both dogs treated successfully with clindamycin, sulfadoxine, and pyrimethamine combination	[370]
India	Punjab (Ludhiana)	NS	Mongrel, adult	Anorexia, anemia, emaciation. Gamonts in blood. Elevated alkaline phosphatase, blood urea nitrogen, and creatinine. Treatment with sulfa-trimethoprim (15 mg/kg) and clindamycin (10 mg/kg) did not cure parasitemia	[371]
India	Uttar Pradesh (Bareilly)	NS	55 dogs admitted to a veterinary clinic for anemia and blood disorders	*H. canis* gamonts in blood smears of 3. The infected dogs were adults, and 1 was co-infected with *E. canis*. All 3 dogs had fever, pale mucous membranes, depression, anorexia, staggering gait, and enlarged lymph nodes. Elevated serum creatinine kinase and alkaline phosphatase. Recovery after medication with tetracycline, DY, and other medicines	[372]
India	Punjab (Ludhiana)	2008–2009	3Three dogs (no additional information)	Gamonts in blood. Fever, anemia, elevated serum creatinine kinase, alkaline phosphatase	[373]
India	Punjab (Ludhiana)	2009–2011	34 retrospectively diagnosed cases. Dogs were 1–14 years old	*H. canis* gamonts were found in blood smears of all 34; alone in 30 and co-infection in 4. Fever, pale mucosae, lethargy, anorexia, vomiting, weight loss, dehydration, diarrhea, corneal opacity, and lymphadenopathy were recorded, in decreasing order	[374]
India	Uttarakhand (Pantnagar)	NS	An 8-year-old Labrador	Inappetence*,* lethargy, weakness, depression, shivering, fever, dyspnea, gamonts in blood smear. Medication with DY (100 mg, twice daily for 10 days) and other drugs improved clinical signs	[375]
India	Punjab (Ludhiana)	NS	10 *H. canis*-positive cases	Fever, anemia. Morphometrics of *H. canis* gamonts reported	[376]
India	Tamil Nadu (Chennai)	NS	A 14-year-old female spitz	Anemia. *H. canis* gamonts in blood smear, hematology data. Concurrent neoplasia (transmissible venereal tumor)	[377]
India	Karnataka (Bidar)	NS	Two cases: one 2–3-month--old German shepherd, and second no description	Anorexia, lethargy, weakness. Fever, lymphadenopathy, anemia, elevated alanine amino transferase, and creatinine kinase. Gamonts in blood smear**. Both cases recovered after medication with DY (10 mg/kg, oral for 24 days)**	[378]
India	NS	NS	A 4-year-old mongrel	Anorexia, dehydration, unstable gait. *H. canis* gamonts in blood smear. Medication with oxytetracycline improved clinical status. Co-infection with *E. canis*	[379]
India	Uttar Pradesh (Izatnagar)	NS	A 3-year-old Labrador retriever	Anorexia, depression, fever. Hepatomegaly revealed by ultrasonography. *H. canis* gamonts in blood smear. Hematology. Dog became aparasitemic after medication with DY (5 mg/kg, oral, twice daily for 28 days)	[380]
India	Punjab (Badal)	2019	A 2-year-old male Labrador retriever	Fever, diarrhea, depression, emaciation, leukocytosis. Gamonts in blood smear. Elevated creatinine kinase and alkaline phosphatase. Dog recovered after medication with ID (5 mg/kg, intramuscularly, 14 days apart) and DY (10 mg/kg, daily for 21 days)	[381]
India	West Bengal (Kolkata)	2020	10 cases	Fever, anorexia, emesis, hematology, anemia, lymphocytosis, elevated alkaline phosphatase, transaminases, gamonts in blood smears. Clinical improvement after medication with DY (5 mg/kg, twice daily for 28 days)	[382]
India	Uttarakhand (Pantnagar)	NS	A 9-year-old male	Fever, anorexia, weakness. Gamonts in blood. Successful recovery after medication with ID, DY	[383]
India	West Bengal (Kolkata)	NS	A 3-year-old male Labrador	Anemia, depression. Hematological and enzyme evaluation. Medication with DY (10 mg/kg body weight, oral, daily for 21 days) was successful	[384]
India	Nagaland	NS	A 3-year-old male	Weight loss, decreased appetite. Gamonts in blood, hematology. Treatment with ID and DY successful	[385]
India	Maharashtra (Mumbai)	NS	A 10-year-old Labrador female	Fever, anemia. Gamonts in blood. Hematology. Treated for *Ehrlichia*. **Sensitivity** to DY and ID	[386]
Iran	Mashhad	2008	An 11-year-old male dog of unknown breed	Anorexia, anemia, rear limb paralysis, hematology, elective euthanasia. Type 2 meronts in histological section of spleen. Gamonts in blood and bone marrow smears	[387]
Israel	Bet Dagan	1968–1972	Two dogs (see Table 2)	Anemia, weakness, fever, gamonts in blood. 1 dog co-infected with *Babesia canis*. **Medication with Ganaseg (5 mg/kg body weight for 2 days)was effective in removing *B. canis* from blood but had no effect on *H. canis* parasitemia)**	[251]
Israel	Be’er-Sheva	1983–1986	Six dogs	Age, breed, clinical and hematological findings tabulated for 6 dogs with *H. canis* gamonts in blood smears. Dogs examined because of fever, depression, and weakness of rear limbs. Medication with tetracycline was evaluated to clear parasitemia	[388]
Israel	Bet-Dagan	NS	A 1-year-old female dog died suddenly and was necropsied	Clinical toxoplasmosis. *H. canis* type 2 meronts were seen in sections of spleen and lymph nodes. There was no cross-reactivity between *Toxoplasma gondii* and *H. canis* in an immunohistochemical test	[389]
Israel	Rehovot	2008–2010	Three dogs with gamonts in blood	Detailed morphological measurements were made on gamonts using image technology. Gamonts were 11.4 × 5.3 µm with mean area of 45.8 µm^2^	[390]
Israel	Rehovot	NS	Case 1. An 11-year-old mixed-breed dog was hit by an automobile, with multiple fractures. Prior to the accident the dog had anorexia and weight loss Case 2. A 7-year-old Doberman pinscher female was admitted to hospital because of anorexia, lethargy, severe weight loss	Case1. The dog had severe anemia, elevated creatinine kinase and alkaline phosphatase. The dog had antibodies to *Ehrlichia*. *Hepatozoon* gamonts were present in 60% of neutrophils and in a few monocytes. Despite medication with ID, DY, the dog died. Histological examination of tissues revealed generalized hepatozoonosis with parasitization of liver, kidneys, heart, and lungs. Numerous type 2 *H. canis* meronts were present in lesions. **This is one of the most severe cases of hepatozoonosis recorded** Case 2. The dog had fever and severe anemia; 70% of neutrophils had *H. canis* gamonts in peripheral blood. The dog was medicated with ID and DY. One month after hospitalization, the dog was still weak and lethargic. Parasitemia reached a level of 90% parasitization of neutrophils. The dog was treated again, and 3 months later the dog had improved clinically, with no demonstrable gamonts in blood	[89]
Israel	Rehovot	NS	A Dalmatian bitch whelped 7 pups that were asymptomatic when weaned. Raised in different homes. (See Table 2)	Four pups developed hemorrhagic enteritis and 1 died. 3 pups were asymptomatic. Clinical signs were related to parvoviral co-infection. Gamonts were found in blood smears of all 7 pups and their dam. One pup medicated with ID (5 mg/kg, by injection) showed clinical improvement	[252]
Israel	Rehovot	1989–1995	**Clinical hepatozoonosis in 100 dogs with *H. canis* gamonts in blood** compared with 100 normal dogs	**Lethargy, emaciation, hyperglobulinemia, hypoalbuminemia, elevated creatinine kinase and alkaline phosphatase. Level of parasitemia correlated with clinical signs. Paper discusses several clinical aspects of hepatozoonosis (see text)**	[88]
Israel	Rehovot	NS	A 6-week-old dog with multiple parasitic infections	Fever, diarrhea. Anemia. *H. canis* gamonts in blood. Co-infected with *Ehrlichia canis, B. canis*, *Cystoisospora* sp., *Giardia* sp., and *Dipylidium caninum*. Medicated with parasiticidal drugs including for protozoa	[391]
Israel	Rehovot	NS	A 4–5-month-old female, mixed breed, hospitalized	Lethargy, inappetence, pain while walking. Swollen limb joints, anemia, gamonts in neutrophils, and monocytes in blood smear. No evidence for other infections. **Radiography revealed polyostotic involvement of bone cortices, periosteal proliferation of limb bones**. Gamonts in joint fluid smears. Medicated with DY and ID. Four months later, remarkable clinical improvement	[392]
Israel	Be’er-Sheva	NS	Litter of 6 pups	**Concurrent infection with *Neospora caninum*.** *H. canis* gamonts in blood of 4; concurrent with *N. caninum* in 3 and *Hepatozoon* alone in 1 pup**. Possible congenital transmission**	[393]
Israel	Rehovot	NS	Three cases. Dog 1, a 5-year-old female. Dog 2, a 7-month-old mixed breed. Dog 3, a 2-year-old male French bulldog	Dog 1, concurrent *Ehrlichia* infection. ***H. canis* gamonts in both neutrophils and monocytes** Dog 2, anemia, ascites, co-infected with *E. canis* and *A. platys*. *H. canis* gamonts in neutrophils. Dog 3, anemia. ***H. canis* gamonts in both neutrophils and monocytes.** Co-infected with *E*. *canis* All 3 dogs treated with ID and DY	[87]
Italy	Rome	NS	A hunting dog	Weight loss, anemia, hematuria, hematochezia, and fluctuating fever. *H. canis* gamonts in blood smears. The dog also had brain malignancy	[394]
Italy	Messina	NS	Two cases Dog 1, an 8-year-old female, mixed breed Dog 2, a 3-year-old hunting dog, male, English pointer breed	Dog 1, weight loss, anemia, generalized lymph adenomegaly, tick infestation. *H. canis* gamonts in blood smears. The dog was suppressed. Concomitant lymphocytic leukemia Dog 2, weight loss, anorexia, dermatological lesions. Concomitant leishmaniosis, and toxicosis due to antimonial treatment. *H. canis* gamonts in splenic punctate smears. The animal died after the clinical examination	[395]
Italy	Sardinia (Sassari, Cagliari, Nuoro, Oristano)	NS	One clinical case and 1 epidemiological study Clinical case: a 6-year-old hunting dog, mixed breed Epidemiological study: 140 dogs, ranging from 5 months to 9 years old	Clinical case: anorexia, jaundice, ataxia, splenomegaly, and hepatomegaly, high body temperature. *H. canis* gamonts in blood smears. Splenectomy was performed but animal died after 1 week Epidemiological study: 23 dogs positive for *H. canis*. All positive dogs lived in rural areas, had a history of tick infestation, and presented jaundice, anorexia, lymph adenomegaly, splenomegaly, and fever. Dogs died about 15 days after a trial of antimonial treatment. Only a few dogs that underwent splenectomy survived. On autopsy, **meronts were also found in lymph nodes, spleen, liver, and synovial fluid**	[396]
Italy	Messina	1980–1981	Nine cases	Gamonts in peripheral blood or bone marrow. Clinical signs not significant	[397]
Italy	Sardinia	NS	34 hunting dogs	Gamonts in blood smears of all. Weight loss, anemia, jaundice, fluctuating fever, limb edema, arthritis, lymphadenitis, and splenomegaly, death after a varying duration. Some dogs died after acute 7–10-day illness, whereas others were chronic. Lesions in 18 necropsied dogs included splenomegaly, hypertrophic liver, nephrosis, partial pulmonary edema and interstitial pneumonia, bilateral polyarthritis	[398]
Italy	Sardinia	NS	Five-year-old female hound dog at the end of pregnancy	Fever, tick infestation, weight loss, splenomegaly. Dead fetuses were extracted by surgery. ***H. canis* gamonts in blood smears. On histopathological examination of the placenta: presence of *H. canis* meronts and gamonts**	[399]
Italy	Imperia	NS	A 7-month-old German shepherd hospitalized	Weight loss, dehydration, inappetence, weakness of rear limbs, fever, thrombocytopenia, elevated alkaline phosphatase and creatinine kinase, **radiography revealed periosteal bone proliferation**. Gamonts in blood smear	[400]
Italy	Pisa	NS	16 cases; 12 1 year old, one 9 months old, three 3 years or older	Clinical signs and other data tabulated for each dog. Depression, anorexia, weight loss in all 16 dogs, fever in 4. Gamonts in blood, up to 70% parasitemia, clinical biochemistry and enzyme elevations detailed, lymphadenopathy Co-infection with *Leishmania* in 2, and *Ehrlichia* in 2	[401]
Italy	Pisa	NS	A 15-month-old female mongrel dog hospitalized because of weight loss and paresis	Fever, pale mucous membranes, dermal lesions, pain in limbs. *H. canis* gamonts in 90% of neutrophils, thrombocytopenia. ***H. canis* meronts, gamonts in bone marrow, lymph node aspirates and bone lesion aspirate**. Diagnosis confirmed by serology and PCR for *H. canis*. Treatment with ID successful. **Rare observation of neurological and bone lesions in *H. canis* infection**	[402]
Italy	Bari	NS	A 3.5-month-old cross-bred dog	Diarrhea, depression, ascites. Anemia, thrombocytopenia. Co-infection with 3 pathogens, *B. canis*, *E. canis*, and *H. canis.* Gamonts of *H. canis* found on the second admission	[403]
Italy	Bari	NS	Three adult crossbred dogs	Gamonts in blood, PCR-positive.** Treatment with ID (6 mg/kg) and DY (10 mg/kg) failed to clear parasitemia**	[101]
Italy	Bari	2013	32 dogs with confirmed *H. canis* infection	**Gamonts in blood. Dogs grouped into 3 treatment regimens of 10 dogs each: (i) ID (5–6 mg/kg, subcutaneously, once weekly for 6 weeks; (ii) toltrazuril/emodepside (15 mg/kg, daily for 6 days) and clindamycin (15 mg/kg daily for 21 days), (iii) untreated group. Both treatment groups failed to eliminate parasitemia**	[103]
Italy	Teramo	NS	A 2-month-old mixed-breed dog, examined twice more at 5 months and 10 months of age. Dog euthanized 2 months later	Initially diarrhea, vomiting, skin lesions. *H. canis* gamonts in blood smear. Systemic hepatozoonosis suspected including hepatitis and dermatitis. ***H. canis* DNA in skin biopsy and in liver**. No mention of the presence of meronts. No other pathogen. Medication with DY and ID cleared gamonts from blood	[404]
Japan	Miyazaki	1990	A 3-year-old Shetland sheepdog	Anorexia, depression, anemia. Co-infected with *Babesia gibsoni.* Gamonts in blood. Special staining to determine if the parasitized host cells are neutrophils or monocytes, probably both	[405]
Japan	Fukuoka Prefecture	1990	A 15-month-old male beagle	Paralysis in hind limbs, due to intervertebral disc which was surgically treated. Elevated alkaline phosphatase. *H. canis* gamonts in blood smear. **Radiography revealed periosteal bone proliferation at the bilateral radii and ulnae, ilia, and right femur**	[406]
Japan	Fukuoka Prefecture	1990–1992	Six litters from 3 bitches	See text	[32]
Japan	Fukuoka Prefecture	1997–1998	Two cases. Case 1: male beagle, 9 months old; its dam was also infected. Case 2: mongrel, 18 months old	Anorexia, depression, stiffness, paralysis. Gamonts in blood. It’s dam was also infected Gamonts in blood smear. Anemia, blood values including serum enzymatic elevation of creatinine kinase and alkaline phosphatase. **Radiography revealed periosteal new bone formation. Muscle biopsy revealed no meronts. Medication with ID, clindamycin, toltrazuril did not improve clinical signs** No clinical signs in case 2, admitted for filarial control	[407]
Japan	Kagoshima	NS	A stray female beagle	Anemia. Prior *Babesia* infection. Acute onset of clinical signs. Gamonts in blood, elevated enzymes, hematology, thrombocytopenia. Medication with clindamycin (10 mg/kg oral), DY (7 mg/kg, oral twice a day) successful	[408]
Korea	Seoul	2015	A 2-year-old Maltese male	Anorexia, depression, fever, dehydration, pale mucous membranes. Anemia, leukopenia, thrombocytopenia. Improved after treatment with antibiotics	[409]
Malaysia	Formerly Federated Mala States	1906	Two dogs, 3-month-old male Pariah and 14-day-old Irish terrier	Emaciated, fever, gamonts in blood of the 15-month-old dog Gamonts found in blood of the 2-month-old pup. First report outside of India	[410]
Mexico	Tamaulipas	NS	A 3-year-old bloodhound	Anorexia, vomiting, weight loss, ptyalism. Hematology, severe neutrophilia, non-regenerative anemia, hyperproteinemia. Dog died. Gamonts in blood smear. *H. americanum* infection suspected	[411]
Mexico	Colima	NS	A 9-year-old boxer	Depression, weight loss, cachexia, fever, anemia, ascites. Gamonts in blood. Hematology. PCR	[412]
Nigeria	Zaria	1980–1981	Four cases identified in an epidemiological study (see Table 2). Dog 1, 14-month-old male German shepherd; dog 2, 16-month-old Labrador retriever; dog 3, 10-month-old mixed breed; dog 4, 1-year-old male Bingo	Fever, anemia. Gamonts in blood. ID, oxytetracycline treatment	[268]
Nigeria	Zaria	NS	18 dogs, 2 months to 6 years old	Fever, anorexia, weight loss, depression, and others. Gamonts in blood of all. **Neutrophil myeloperoxidase deficiency reported**	[413]
Philippines	Manila	1971	Eight cases	Fever, anemia. Dogs were tested at veterinary clinic for routine examination. *H. canis* gamonts in blood smears. Data for all 8 dogs tabulated. Three of the dogs were considered to have clinical hepatozoonosis	[414]
Poland	Various veterinary practices	2020	Five cases, 3 months to 10 years old	Five dogs with clinical signs, lethargy, poor condition, weight loss. Hematology. Concurrent infections with other parasites in blood in all 5. *H. canis* gamonts and DNA in blood	[275]
Romania	Snagov, Ilfov County	NS	A 1-year-old German shepherd female	Anemia, apathy. *H. canis* DNA in blood. Co--infection with *A. platys*. Medicated with ID (6 mg/kg) once. Clindamycin for 21 days. Full recovery after 4 months	[415]
Russia	St. Petersburg	NS	Young dog	Right pelvic limb lameness. Gamonts in blood smear and DNA. Treatment with numerous medicines including DY, ID successful	[416]
Senegal	NS	1980–1981	Two Dobermans from the same household. Dog 1, an 11-year-old male. Dog 2, a 24-month-old male	Dog 1: anorexia, weight loss, hair loss and itching, enlarged popliteal and prescapular lymph nodes, epileptiform seizures. Hematology: *H. canis* gamonts in neutrophils in blood smears. Despite treatments (Berenil, Levamisole, Bactrim, Flavoquine), clinical signs persisted; euthanized Dog 2: intense itching, all limb edema, enlarged popliteal lymph nodes. Despite treatments (Berenil, Levamisole, Bactrim, Flavoquine), clinical signs did not improve; euthanized	[417]
Serbia	Belgrade	2018	Three cases. Dog 1, 3-month-old Rottweiler. Dog 2, 8-year-old miniature Schnauzer. Dog 3, a 4-year-old mixed-breed dog from shelter	All 3 dogs co-infected with *E. canis. H. canis* infection was confirmed by finding gamonts in blood smear or by PCR. Details of clinical signs, hematological and biochemical data and treatment tabulated. Anemia, weakness common to all 3 dogs	[418]
South Africa	Kruger National Park	NS	20 dogs	Tissues of 20 dogs that died naturally were studied histologically; 12 were uncomplicated, 3 had babesiosis, 1 had adenocarcinoma,1 had distemper viral infection, and 1 had *Spirocerca lupi* infection. **First histological study describing different types of meronts. Meronts in visceral tissues but also in brains of 2, heart of 1, and skeletal muscle of 1. Type 1 meronts 1–4 merozoites in lymph node. Type 2 meronts numerous in spleen**	[419]
Spain	Girona, Catalina	1992	Eight cases	Gamonts in blood. Clinical signs, co-infections with *Ehrlichia* and *Leishmania*, and treatment listed for each dog. Anorexia and lymphadenopathy predominated. Hepatozoonosis reviewed	[420]
Spain	Córdoba	NS	A 4-month-old mixed-breed dog	A fully vaccinated dog died within 1 week of onset of clinical signs. Fever, loss of appetite, hyperesthesia, muscular disorders. Complete necropsy performed. **The dog had generalized hepatozoonosis with lesions in spleen, liver, lungs, kidneys associated with *H. canis.*** TEM of circulating gamonts reported	[421]
Spain	Córdoba	NS	Number of dogs studied not mentioned	Dogs had fever, loss of appetite, weakness, splenomegaly. Tissues from necropsied dogs and those obtained by biopsy were studied ultrastructurally. Meronts were found in spleen. **TEM of merozoites and gamonts reported**	[422]
Sri Lanka	Peradeniya	NS	Five dogs, 5 months to 7 years old	Clinical signs listed for each dog. All cases were compromised with other ailments. Hepatozoonosis appears to be secondary. Gamonts and *H. canis* DNA in blood. **All patients medicated with ID showed clinical improvement**	[423]
Sweden	Uppsala	NS	A 10-year-old, female Swedish flat-coated retriever	Lethargy, poor appetite, polydipsia. Depression, fever, thrombocytopenia. Initially medicated with DY for treating *Anaplasma. ****H. canis* gamonts exclusively found in monocytes and not in neutrophils and diagnosis confirmed by PCR**	[90]
Thailand	Bangkok	2001–2003	342 cases, retrospective study, veterinary clinic	*H. canis* gamonts in blood smears of all. Fever was common. Hematological and biochemical values were evaluated. 8% had hypocytic hypochromic anemia. 19% had elevated alanine phosphate, and alanine transaminase, and 13% had azotemia	[424]
Thailand	Nakhon Pathom	NS	31 cases, retrospective study	18 low (parasitemia, < 80% gamont-infected neutrophils), 13 high parasitemia (> 80% gamont-infected neutrophils). No differences in hematological values. Two *H. canis* sequences Thailand 1 and 2 deposited	[425]
Trinidad, West Indies	NS	NS	Two cases from the same household; both 5-year-old mixed-breed females	Case 1: anorexia, muscle hyperesthesia, anemia Case 2: neutrophilia **Gamonts in neutrophils and monocytes in blood smears of both dogs.** DNA sequences evaluated from both dogs	[426]
Turkey	Aydın	2002	A 5-year-old female, mixed breed	Anorexia, depression, fever. Gamonts in blood. Blood values. Treatment with toltrazuril (10 mg/kg, orally, daily for 5 days) trimethoprim-sulfamethoxazole (15 mg/kg, intravenously) effective in relieving clinical signs	[427]
Turkey	Aydın	NS	14 mixed-breed dogs	Gamonts and *H. canis* DNA in blood of all 14 dogs. Lymphadenopathy and skin lesions in 14, fever in 9, anorexia in 8, lethargy and weight loss in 7. **Compared to 10 healthy controls, levels of nitric oxide, serum glutathione, and malondialdehyde elevated**	[428]
Turkey	Aegean region	NS	10 mongrel 2–8-year-olds	Anorexia, depression, fever, lymphadenopathy in all 10, dermal lesions in 8. All dogs had normochromic anemia, hypoalbuminemia in 9 dogs, elevated creatinine kinase in 8. Concurrent *E. canis* in 8*, A. platys* in 6, and *A. phagocytophilum* in 4 dogs	[429]
Turkey	Aegean region	NS	32 (31 > 1 year old), 17 males, 15 females, different breeds (detailed in the paper)	Mono *H. canis* infection in 16, co-infections with *E. canis*. Anorexia, lymphadenopathy, tachypnea, and fever common. Anemia and thrombocytopenia are the most common. Paper provides details of clinical signs and laboratory findings in groups of both infected and noninfected dogs	[430]
Turkey	Balıkesir	NS	A 4-year-old cross-bred female	Fever, anemia, weight loss. Gamonts in blood. Hematology. Medication with DY (10 mg/kg, orally, 21 days) had no improvement	[431]
UK	**3 imported dogs**	NS	One dog imported from Ireland, and 2 imported from Cyprus	Anorexia, lethargy, pale mucous membranes, indicative of anemia. *H. canis* gamonts in blood smears and diagnosis confirmed by PCR. Medication with ID not effective	[102]
USA	New Jersey	NS	A 7-month-old mixed-breed female	A 2-cm-diameter pruritic, alopecic nodule over the right eye. Needle aspirate cytology revealed degraded neutrophils and eosinophils with bacteria. *H. canis* gamonts in aspirate smear. DNA extracted from whole blood revealed *H. canis*, distinct from *H. americanum*. **This is first histologically confirmed *H. canis* infection in dogs in the USA**	[86]
Venezuela	Aragua State	1996	One-year-old male dog	Anemia. *H. canis* gamonts in blood. Co-infection with *Ehrlichia platys*	[432]

Information in bold = unusual or important, *Babesia canis=B. canis*, *Ehrlichia canis=E. canis*

*DY* doxycycline, *ID* imidocarb dipropionate, *NS* not stated

Compared with the Argentinian study of dogs in the general population [74], the prevalence of *H. canis* was higher in dogs that were admitted to a veterinary clinic in Chennai, India [84]. *Hepatozoon canis* gamonts were found in blood smears of 1986 (13.2%) of 14,992 dogs (Table 2). Additionally, *Babesia gibsoni* was highly prevalent (56.6%) in these dogs [84].

The prevalence of *H. canis* infections in the USA is considered rare. However, in a review of dog samples submitted to the Molecular Diagnostics Laboratory in Auburn, Alabama, *H. canis* DNA was detected in nine (11.7%) of 77 dogs positive by PCR for *Hepatozoon* spp. [85]. As shown in Table 3, there is also a report of clinical *H. canis* infection in a dog from New Jersey, USA [86].

#### Clinical infections, diagnosis, and treatment

Worldwide reports of clinical *H. canis* infections in dogs are summarized in Table 3. Dogs of any age, from fetuses to old dogs, can be affected. There is no known breed or sex predisposition. Because *H. canis* is transmitted by ticks, dogs are often concurrently infected with other tick-transmitted pathogens such as *Ehrlichia*, *Babesia*, and *Anaplasma*. *Ehrlichia canis* morula and *H. canis* gamonts have even been found in the same monocytes [87]. There are only a few confirmed reports of *H. canis* infections in which other disease conditions and infections have been reliably ruled out.

Anorexia, weight loss, fever, and lethargy are the most consistent clinical signs. The most detailed information is derived from a case–control study of 100 naturally infected dogs compared with 100 uninfected dogs in a veterinary teaching hospital in Israel [88]. Anemia, hyperglobulinemia, high parasitemia, hyperproteinemia, and elevated serum biochemical values, especially creatinine kinase, were the most common laboratory findings. Clinical signs were related to parasitization of specific organs, from eyes to periosteum (Table 3). In severe cases, dogs can die of generalized hepatozoonosis [89].

**Fig. 6:**
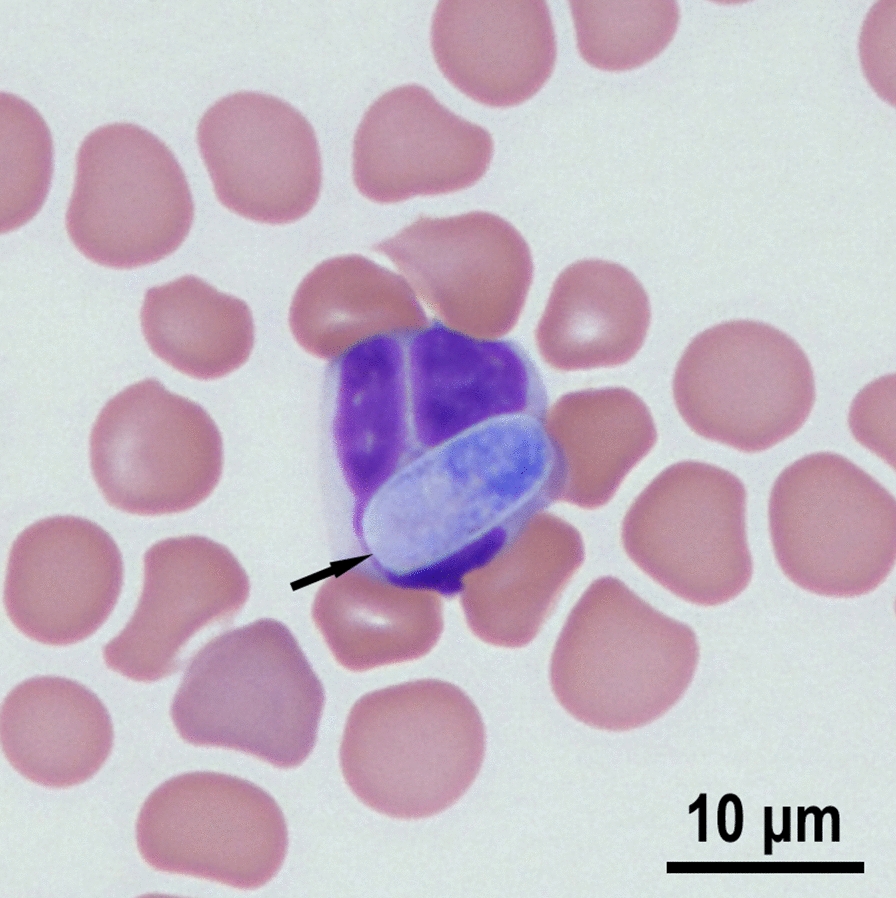
*Hepatozoon canis* gamont in a neutrophil (arrow) in blood smear of a naturally infected dog in Israel. May–Grünwald Giemsa stain

Diagnosis can be made by finding gamonts in blood smears stained with any Romanowsky-type blood stains such as Giemsa or commercially available rapid stains. *Hepatozoon canis* gamonts primarily occur in neutrophils (Fig. 6), but monocytes can also be infected [90]. However, this method is insensitive to detecting low-grade infections. PCR is much more efficient [81] (also see Table 3). The gene commonly used for PCR is the *18S* rRNA gene. Real-time PCR can quantify the parasitic load [91, 92]. An analysis of publicly available sequences of *H. canis* from 46 countries revealed 76 haplotypes [93], and other studies have also reported considerable genetic diversity [24, 94]. Molecular diagnosis is further discussed in the section “Molecular detection of *Hepatozoon* spp. in carnivores.”

Although serological tests (indirect fluorescent antibody [IFA] test, and enzyme-linked immunosorbent assay [ELISA]) have been developed to detect *H. canis* antibodies, these tests are not employed in routine diagnosis because of the difficulties in obtaining and preparing antigens [95–97]. However, they have been used in serological surveys in several countries [98–100].

*Treatment:* Imidocarb dipropionate (ID) is the most frequently used drug. It is a urea derivative compound used in veterinary medicine to treat piroplasm infections (babesiosis, theileriosis). It is licensed for use by veterinarians, administered intramuscularly or subcutaneously. It is well tolerated, but adverse effects include cholinergic signs, such as salivation, nasal drip, and vomiting. The usual dose is 5–6 mg/kg body weight, repeated twice a month until gamonts are undetectable in blood. The drug is successful in decreasing the parasitic load but not effective in eliminating *H. canis* parasitemia [101–103].

Other antiprotozoal drugs used to treat other protozoal infections in dogs such as trimethoprim-sulfonamide, clindamycin, and toltrazuril have been used as well (Table 3).

### *Hepatozoon americanum* infections

*Hepatozoon americanum* infections are discussed separately because of the geographical distribution and different clinical presentations. Infections are mostly confined to the south-central and southeastern USA [21], coinciding with the distribution of the transmitting tick, *Amblyomma maculatum*.

Data on *H. americanum* prevalence are limited. *Hepatozoon* sp. DNA was detected in the blood of three of 200 shelter dogs from Oklahoma, USA [85]. In the same study, one of 16 (6.2%) dogs from Oklahoma was infected with *H. americanum*. Additionally, two dogs with clinical signs were PCR-positive for *H. americanum* DNA [85].

Most reports of *H. americanum* clinical infections are from Alabama, Texas, and Oklahoma (Table 4). It is a severe debilitating illness and often results in death [22, 104, 105]. Clinically, dogs of any age can be affected. Fever and bone and muscle pain are the most common clinical signs. Biochemistry profiles, blood cytology, radiography, muscle biopsy, and PCR can aid diagnosis. Persistent leukocytosis and elevated serum alkaline phosphatase are the most consistent hematology and serum biochemistry findings. Moderate to marked leukocytosis are reported, with neutrophilia and leukocyte counts which can vary from 30,000 to 200,000/μl blood. Radiographically, periosteal lesions are common, with periosteal proliferation, periostitis, and new bone formation. Muscle biopsy can reveal granulomatous lesions that may or may not contain typical onion skin meronts. A PCR test on the DNA isolated from muscle biopsy or blood can confirm the diagnosis. Immunohistochemical staining with *H. americanum* antibodies has been used to confirm tissue stages in histological sections [106].

**Table 4:** Descriptions of clinical *Hepatozoon americanum* hepatozoonosis in dogs in the USA

Year	State	No. of dogs	History and background	Findings	References
1976–1977	Texas	3	Fever, depression, weight loss	Radiographic evaluation revealed bone lesions and bone proliferation in 2 dogs. Gamonts in blood	[433]
1980	Texas	1	Rear limb weakness, depression	Periosteal new bone formation. Gamonts in blood smear	[434]
1983–1984	Texas	50		Value of muscle biopsy in diagnosis of canine hepatozoonosis demonstrated. Onion skin meronts found in muscles	[435]
1985	Texas	15	Four months to 9 years old	Fever, anorexia, weight loss, pain, gait abnormalities, **10 dogs with radiographic abnormalities**, gamonts in blood smears of 9, meronts in muscle biopsy of 9, 5 dogs euthanized. Details tabulated for each dog	[436]
1985	Louisiana	1	An adult miniature poodle	Anorexia, depression, fever, epistaxis, thrombocytopenia. Alkaline phosphatase elevated. *H. canis*-like gamonts in buffy coat. No periosteal bone lesion. Co-infection with *Ehrlichia*	[437]
1988	Oklahoma	1	Ten-month-old cocker spaniel born in Oklahoma and shipped to Pennsylvania when 8 weeks old	**Glossitis, pharyngitis**, weight loss, inappetence, fever. Anemia, fever, radiograph revealed pneumonia. Biopsy of the oral lesion revealed multifocal pyogranulomatous inflammation including myositis. *H. canis* merozoites in biopsy. The dog recovered without treatment	[438]
1889–1994	Alabama	22 cases	14 purebred, 8 months to 7 years old	Fever, pain, stiffness and paresis, leukocytosis, mild anemia, elevated alkaline phosphatase. **Gamonts not detected in blood smears of whole blood or buffy coat, or bone marrow. Periosteal bone proliferation in 18 of 22 cases. Diagnosis by finding onion skin meronts in muscle biopsies. Antiprotozoal treatment not effective**	[439]
1989–1998	Alabama	53 cases	Several breeds, mean age 3 years	**Diagnosis confirmed by muscle biopsy in all 50 dogs. Gamonts in peripheral blood of 12 of 53 dogs. Various drug regimens were tried. Antiprotozoal treatment prolonged life but did not eliminate meronts in tissues**	[440]
1997	Oklahoma	4 cases	3–7 years old	Fever, marked neutrophilic leukocytosis, periosteal bone proliferation, myalgia, weakness. Gamonts in blood smear of 1 and by biopsy in others. Myositis and onion skin meronts. **First report from Oklahoma**	[441]
1998	Oklahoma	19 cases	Biopsy of muscle from 7 and tissues from 12 dogs that died	**Morphogenesis of lesions and meronts described**	[442]
2025	Texas	1 case	1-year-old Rottweiler	**Bilateral muscle atrophy of temporalis muscles. Development of *H. americanum* meronts described**	[27]

Information in bold = important

Treatment of *H. americanum* infection includes a triple oral therapy of trimethoprim-sulfadiazine (15 mg/kg, every 12 h), pyrimethamine (0.25 mg/kg, every 24 h), and clindamycin (10 mg/kg, every 8 h) for 14 days [19]. An alternative to the triple drug treatment is ponazuril (10 mg/kg orally every 12 h) for 14 days. Non-steroidal anti-inflammatory drugs (NSAIDs) are used to alleviate pain, fever, and inflammation in the first 1–2 weeks of treatment [19]. After relief from clinical disease is achieved, remission is prolonged with oral administration of the coccidiostat decoquinate (15 mg/kg mixed in food every 12 h for 2 years) [19]. Relapse of clinical signs is possible during treatment or after discontinuation of medications.

**Fig. 7:**
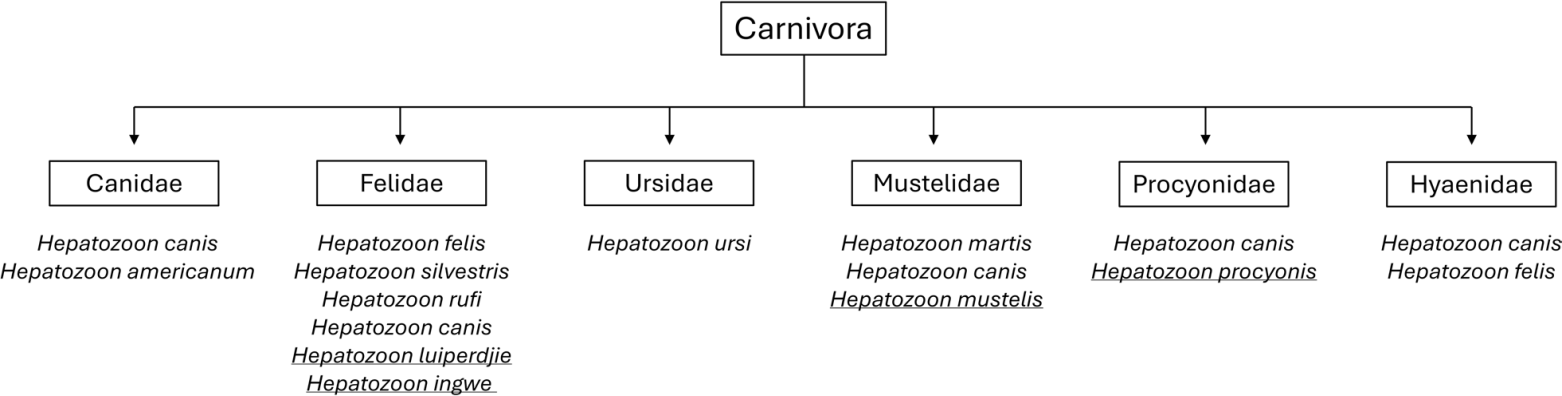
Distribution of *Hepatozoon* species across Carnivora

## Hepatozoonosis in domestic cats

### Prevalence

Compared to dogs, *Hepatozoon* infections are less common in cats (Table 5). Prevalence varied with the population of cats surveyed and methods used to detect infections. After the discovery of *Hepatozoon* gamonts in blood smears of nine of 374 cats in India more than a century ago [107], nothing was published for the next six decades, until Klopfer et al. [108] found meronts of the parasite in histological sections of the hearts of 15 of 50 dead cats brought to necropsy and 21 of 50 normal cats in Israel; no gamonts were detected. These findings were confirmed by a retrospective study of 1229 cats from Israel; *Hepatozoon* sp. gamonts were detected in the blood of only seven of these cats [109]. However, 36% of cats in a later study from Israel were positive for *H. felis* infection by PCR [110]. Subsequent studies revealed that blood smear examination is insensitive compared to the detection by PCR [111–115]. A multicenter study of 100 cats from each of six Mediterranean countries revealed that the prevalence was significantly higher in cats from Greece (30%) and Portugal (29%) followed by Spain (15%), Israel (15%), France (4%), and Italy (0%) [116].

**Table 5:** Prevalence of *Hepatozoon* sp. infections in domestic cats

Country	Locality/region	Year sampled	Source^a^	No. of cats positive/no. examined (%)	Diagnostic methods^b^	Comments	References
Angola	Luanda	2014–2016	D	3/102 (2.9)	PCR	*H. felis* DNA. Concurrent infection with *E. canis* in 1 cat	[443]
Brazil	São Paulo	NS	NS	3/3 (100)	PCR	**Sequence aligned with *H. canis***	[444]
Brazil	Maranhão	2008–2009	ST, D	1/200 (0.5)	PCR	*H. felis* DNA in blood, sequence deposited	[445]
Brazil	Mato Grosso	2009–2011	SE, VC	0/178	PCR		[446]
Brazil	Mato Grosso do Sul	2013	D, ST	1/151 (0.6)	PCR	**Sequence aligned with *H. americanum***; co-infections with other pathogens	[447]
Brazil	Mato Grosso do Sul	2011–2013	D, SE, VC	3/180 (1.6)	PCR	3 of 65 cats from Várzea Grande were PCR-positive. *Hepatozoon* sp. Cuiabá sequence deposited	[448]
Brazil	Rio de Janeiro	NS	Sterilization program	21/28 (75.0)	PCR	*H. felis* DNA	[449]
Cape Verde	Maio Island	2012	D	12/80 (15.0)	C, PCR	No gamonts in buffy coat smears. *H. felis* sequences deposited	[111]
Cyprus	6 districts	2014	VC	66/174 (37.9)	PCR	Of 66 cats infected with *Hepatozoon*, mono-infection in 49; co-infections with other pathogens in 17	[450]
France	Several areas	2006–2007	VC	2/116 (1.7)	PCR	*H. canis-*like DNA	[212]
France	NS	NS	Multicenter survey, SP	4/100 (4.0)	PCR		[116]
Germany	Retrospective data search	2007–2020	D	64/931 (6.9)	PCR	47 infected cats had been imported. Sequencing revealed *H. felis* in 16 cats	[451]
Greece	NS	NS	Multicenter survey, SP	30/100 (30.0)	PCR		[116]
Greece	Skopelos	2023	D	45/54 (83.3)	C, PCR	Gamonts in blood smear of 6 (2 cats were PCR-negative) and *Hepatozoon* sp. DNA in 43	[112]
Hungary	Several areas	2021–2022	S, ST	1/127 (0.7)	C, PCR	*H. felis* DNA, no gamonts in blood smear	[452]
India	Tamil Nadu (Chennai)	1905–1908	ST	9/374 (2.4)	C	Gamonts in blood	[107]
India	Telangana (Hyderabad)	2007–2010	D	2/4 (50.0)	PCR	*H. felis* DNA	[228]
India	Kerala	2018–2019	D	10/111 (9.1)	C, PCR	No gamonts in blood smears. *H. felis* DNA in blood	[113]
India	Kerala	NS	D	7/122 (5.7)	C, PCR	No gamonts in blood smears. *H. felis* DNA in blood	[114]
Iran	7 provinces	2018–2023	D	29/774 (3.7)	PCR	*Hepatozoon* PCR-positive; **sequencing revealed *H. felis* in 25 and *H. canis* in 3**	[453]
Israel	Bet-Dagan	NS	Retrospective study 50 dead cats and 50 for various studies	36/100 (36.0)	C	**First description of *Hepatozoon* meronts in the heart**	[108]
Israel	Rehovot	1989–1995	Retrospective study, VC	7/1229 (0.5)	C	Gamonts in blood of 7 cats; 2 co-infected with *Hemobartonella felis*, and 4 co-infected with retroviral infections (FIV, FELV). Elevated enzymes	[109]
Israel	Several locations	2010–2011	Several locations and sources	55/152 (36.2)	PCR	**Data used for redescription of ** ***H. felis***	[110]
Israel	Central Israel	2019–2022	Multicenter survey, SP	15/100 (15.0)	PCR	Cats with outdoor access admitted to the veterinary teaching hospital	[116]
Italy	Bari, Lecce, Matera	NS	D, ST	10/196 (5.1)	PCR	**Three *Hepatozoon* species found:* H. felis* in 8, *H. silvestris* in 1, and *H. canis* in 1**	[454]
Italy	Northeast, from 3 sites: Veneto, Friuli-Venezia Giulia, Trentino-Alto Adige	NS	D	26/158 (16.5)	C, PCR	*Hepatozoon* DNA in blood; *H. felis* in10, *H. silvestris* in 6. Gamonts not found in blood smears. 25 co-infected with FIV or FELV	[455]
Italy	Northeast, from 3 sites: Veneto, Friuli-Venezia Giulia, Trentino-Alto Adige	NS	D	31/206 (15.0)	PCR	*Hepatozoon* DNA in blood; *H. felis* in 12, *H. silvestris* in 19	[153]
Italy	NS	NS	Multicenter survey, SP	0/100 (0)	PCR		[116]
Japan	Tsushima	2012–2015	D	7/284 (2.4)	PCR	*H. felis* DNA in blood	[456]
Philippines	Cabanatuan, San Jose, Munoz	2017–2018	D	1/115 (0.8)	PCR	*H. canis* DNA	[195]
Portugal	North and Central	NS	VC	3/320 (0.9)	PCR	Three cats were co-infected with *B. vogeli* and *H. felis*, 1 with *H. felis*, and *Leishmania infantum,* and 2 with *H. felis* and *Rickettsia*	[457]
Portugal	NS	NS	Multicenter survey, SP	23/100 (23.0)	PCR	14 *H. felis*, 1 *H. silvestris*	[116]
Romania	NS	2017–2019	VC	2/371 (0.05)	PCR	Survey for piroplasms	[458]
South Africa	Limpopo	NS	One roadkill, 15 from tissue banks/collections	11/16 (68.7)	PCR	**Multiple distinct haplotypes of *H. felis* were identified**. **One genotype was related to *H. apri* from wild boar. The 3 haplotypes from cats from South Africa were not closely related**	[72]
Spain	NS	NS	VC	2/330 (0.6)	PCR	All cats sampled were clinically ill. *H. canis*-like DNA in both cats. First report from Iberian Peninsula in Spain	[145]
Spain	Barcelona	NS	ST	4/25 (16.0)	PCR	Stray cats surveyed for hemoprotozoa	[459]
Spain	Barcelona	Retrospective study	VC	4/100 (4.0)	PCR	*H. felis* DNA in all 4 cats, concurrent infections	[460]
Spain	Madrid	2005–2008	D	10/644 (1.6)	PCR	*H. felis* in 10, ***H. canis*** in 1	[461]
Spain	NS	NS	Multicenter survey, SP	15/100 (15.0)	PCR		[116]
Spain	Zaragoza	2020–2022	ST	85/332 (25.6)	PCR	Co-infections with other blood pathogens, including FIV, FeLV,	[462]
Spain	Murcia	NS	D	31/123 (25.2)	PCR	***H. felis* and no *H. canis* DNA.** 28.8% cats with clinical signs and 22.2% without clinical signs	[285]
Thailand	Bangkok	2002–2003	Semi-domesticated at 3 monasteries	97/300 (32.3)	C, PCR	*Hepatozoon* sp. gamonts in 2 and DNA in blood of 97	[115]
Thailand	Bangkok	NS	Semi-domestic	12/372 (3.2)	PCR	Survey for vector-borne infections. Anemia in 2 *Hepatozoon* infected cats	[463]
Turkey	Tekirdag	2017–2021	VC	18/167 (10.7)	PCR	*H. felis* DNA from buffy coat. Concurrent infections with other blood pathogens	[464]
Turkey	NS	2013–2014	VC (sterilization)	24/1012 (2.3)	PCR	*H. felis* DNA in blood;18 sequenced, 9 haplotypes	[465]
Turkey	Samsun	2021	D	58/311 (18.6)	C, PCR	*Hepatozoon* sp. gamonts in blood of 5, and DNA in 58. **Sequence analysis revealed* H. felis* in 25 cats and *H. silvestris* in 33**	[466]
Ukraine	Translocated to Poland after Russian invasion	2022	D	1/1	PCR	*Hepatozoon* sp. DNA	[308]
USA	Oklahoma	NS	D	1	PCR	*Hepatozoon* sp. DNA	[52]

*FIV* feline immunodeficiency virus, *FELV* feline leukemia virus, *NS* not stated

^a^*D* domestic, *SA* survey for arthropods, *SP* survey for other pathogens, *ST* stray, *VC* admitted to veterinary clinic

^b^*C* cytology, blood smears, *PCR* *18S* rRNA gene, *S* serology

Information in bold = important

Invertebrate hosts for *Hepatozoon* species in domestic cats are unknown, but ticks are suspected [9, 117, 118].

### Clinical infections

There are only a few reports of clinical hepatozoonosis in cats, and these are summarized in Table 6. Although most reports were associated with concurrent immunosuppressive conditions (feline immunodeficiency virus [FIV], feline leukemia virus [FELV]), severe disease due to *H. silvestris* infection in the absence of other pathogens has been reported [119, 120]. Anemia, fever, azotemia, halitosis, polydipsia, and anorexia were reported (Table 6). There is a recent report of polyarteritis and new formation in a cat [121] and another case of *H. felis* bone infection from Austria described leukopenia, thrombocytopenia, and increased serum symmetric dimethylarginine (SDMA) and bilirubin in a cat which recovered following treatment [122]. Treatment efficacy of different drugs used is summarized in Table 6.

**Table 6:** Clinical *Hepatozoon* sp. infections in domestic cats

Country	Year Sampled	History	Comments	References
Austria	NS	A 6-year-old from Burgenland state that never left Austria	Lethargy, fever, painful abdomen, icterus. Hematological and biochemical evaluation revealed leukopenia, elevated bilirubin, *Hepatozoon* sp. gamonts in blood, and *H. felis* diagnosis confirmed by PCR on blood. Medicated with ID (6 mg/kg, twice daily, repeated after 14 days) and doxycycline monohydrate (5 mg/kg, daily, orally, for 4 weeks). Clinical signs disappeared and the cat recovered. No evidence for other pathogens or immunosuppression	[122]
Brazil	NS	Three cats in the same household	Cat 1, a 10-year-old male, was hospitalized because of polyuria, polydipsia, halitosis, and vomiting for a week. Anemia, lymphadenopathy, leukopenia, elevated serum creatine kinase, and alkaline phosphatase Cat 2, a 4-year-old, and cat 3, a 10-year-old, appeared clinically normal but had elevated creatinine kinase and alkaline phosphatase Gamonts in blood smears of all 3 cats	[467]
Cyprus	2014	A 6-year-old domestic shorthair hospitalized	Multiple ulcerated dermal nodules considered due to *Leishmania*. Co-infected with *Mycoplasma* and *H. felis* based on PCR	[468]
France	NS	Two 1-year old males from southern France	Lethargy, anorexia, anemia, weight loss, and gingivitis. Gamonts in blood smears of both. Both cats seropositive for FELV and negative for FIV, and both euthanized. Cat 1 necropsied. Type 2 meronts in histological sections of myocardium and skeletal muscle, but not in liver, lung, or spleen. Cat 2, not necropsied	[469]
France	NS	A 13-year-old, neutered male	Lymphoma on left maxillary, FELV-negative but FIV-positive. *Hepatozoon* gamonts in blood smear. Chemotherapy for lymphoma unsuccessful. Euthanized after 8 months. *Hepatozoon* gamonts in blood smear and type 2 *Hepatozoon* meronts in skeletal muscle	[470]
Iran	2022	A 6-year-old male shorthair hospitalized because of weakness and anorexia	Fever, painful abdomen, mild jaundice and tick infestation. FIV- and FIPV-negative. *H. felis* gamonts and DNA in blood smear. Hematology revealed anemia, elevated liver enzymes, azotemia, elevated creatinine and urea. Medication with ID at 14-day interval and DY twice a day for 14 days cleared parasitemia and relieved symptoms	[471]
Israel	NS	A 1-year-old male shorthair	Weakness, hypersalivation, halitosis. Gamonts in blood smear. Elevated lactate dehydrogenase and creatine kinase. FELV- and FIV-negative. Medicated with DY (5 mg/kg, once daily for 10 days). Clinical signs and parasitemia resolved	[472]
Italy	NS	An 11-year-old neutered shorthair	Clinical-intestinal intussusception, gamonts in blood, *H. silvestris* DNA in blood. Negative for FIV, FELV	[120]
South Africa	NS	An 18-month-old male house cat from Warmbad, Republic of South Africa	Anorexia, stomatitis, gingivitis, fever, weakness, dehydration. Gamonts in blood smear. Oxytetracycline (50 mg/kg twice daily for 7 days), day 6 after admission; primaquine (2 mg orally, once) administered on day 8. From day 9, the cat’s appetite began to improve—temperature was still 40.8 °C. From day 10 the temperature began to fall and was normal from day 12. Blood smears were repeated on day 14, with no *Hepatozoon* gamonts observed/reported	[473]
Switzerland	2017	A 5-year-old neutered male, shorthair cat in a clinic in Meiringen	*H. silvestris*-associated clinical hepatozoonosis. Lethargy, weakness, anorexia. No evidence for immunosuppression and had been immunized against FIV and FELV. It suffered severe myocarditis in both auricles and ventricles; its numerous type 2 *H. silvestris* meronts were confined to the heart; the pancreas, kidneys, stomach, intestines, brain, and bone marrow were not affected	[119]
Turkey	NS	A 5-year-old female	Fever, anorexia lymphadenopathy, icterus, elevated bilirubin, concurrent infection with FIV. Gamonts in blood	[474]
UK	NS	A 10-year-old female shorthair	Lethargy, anorexia, fever, neutrophilia, persistent fever. *H. felis* DNA in blood. **Radiography revealed polyarthritis, new bone formation.** No other pathogen**. Medication with DY (10 mg/kg, twice daily) for 4 weeks relieved clinical signs and cleared parasitemia**	[121]
USA	2024	A 7-year-old female Siamese-mixed breed	Cardiac murmur, hypoalbuminemia, transient cardiopathy. One gamont in blood smear. PCR amplification revealed *H. silvestris*. The cat was FELV- and FIV-negative. **First demonstration of *H. silvestris* gamont stage in peripheral blood smear for this species. First confirmed case of clinical hepatozoonosis in domestic cat in USA**	[154]

*FIV* feline immunodeficiency virus, *FELV* feline leukemia virus, *FIPV* feline infectious peritonitis virus, *DY* doxycycline, *ID* imidocarb dipropionate, *NS* not stated

Information in bold = important

## *Hepatozoon* infections in wild Carnivora

The prevalence of *Hepatozoon* infection in different species of wild carnivores is summarized in Tables 7, 8, and 9 and Fig.7.

**Table 7:** *Hepatozoon* infections in wild canids

Country	Region, locality	Year sampled	Source^a^	Diagnostic method^b^	No. positive/no. tested (% positive)	Findings	References
FOXES							
Red fox (*Vulpes vulpes*)							
Austria	Tyrol, Vorarlberg	2013–2015	S	PCR	151/506 (29.8)	*H. canis* DNA in 151 of 506 (29.8%) spleens, and 65 (18.5%) of 351 blood samples. ***H. canis* DNA found in fetal tissues of 2 of 6 fetuses from an infected dam**	[31]
Austria	Gänserndorf	2014	S	PCR	21/36 (58.3)	*H. canis* DNA in spleens. Co-infection with *Babesia* in 4	[475]
Bosnia and Herzegovina	Six regions	2013–2014	S	PCR	46/119 (38.6)	*H. canis* DNA in spleen	[476]
Bosnia and Herzegovina	Sarajevo	2019	Natural death (see text)	HE, PCR	1/1 (100)	**Clinical. Many type 2 meronts in bone marrow, spleen, lymph nodes, and lungs**. **Co-infection with *Leptospira* (see text)**	[140]
Croatia	Four regions	2007–2008	S	PCR	46/191 (24.0)	*H. canis* DNA of 44 and *Hepatozoon* sp. in 2 spleens.	[477]
Czech Republic	Military Region and Training Area Hradiště	2014–2015	S	PCR	20/21 (95.0)	*H. canis* DNA from blood	[208]
France	Sommières	2003	H	C	1/1 (100)	Asymptomatic	[352]
Germany	Thuringia	2009	Dead	PCR	118/261 (45.2)	Spleens from tick-infested foxes were positive for *H. canis* DNA	[478]
Hungary	Seven regions	NS	S	PCR	26/334 (7.7)	*H. canis* DNA in blood. Sequences from 14 foxes were deposited	[479]
Israel	NS	1999–2001	T	Antibodies, ELISA	20/84 (23.8)	Wild-rabies vaccination study. *H. canis* antibodies detected by ELISA	[480]
Israel	Multiple regions	NS	T	PCR	9/21 (43.0)	*H. canis* DNA from blood and spleen	[147]
Italy	Central Apennines	2005–2006	S	HE, PCR	16/119 (13.4)	*H. canis* DNA in 16 spleen homogenate and type 2 meronts in histological sections of 4 spleens	[481]
Italy	Pisa	2014–2016	S	PCR	75/153 (49.0)	*H. canis* DNA detected in spleens. Co-infections with *E. canis* in 47	[482]
Italy	Piedmont	2009–2017	R	PCR	8/156 (5.1)	*Hepatozoon* sp. DNA from tissues	[483]
Morocco	NS	2003–2011	NS	PCR	4/10 (40.0)	*Hepatozoon* sp. DNA	[12]
Netherlands	NS	NS	Dead	PCR	76/174 (43.7)	*H. canis*	[66]
Poland	Mazovian, Masurian Lakeland	NS	S	PCR	16/138 (11.6)	Two *H. canis* sequences were different from each other	[484]
Portugal	Several areas (northern, central, and southern)	2008–2010	S, R	HE, PCR	68/90 (75.6)	***H. canis* DNA in 68. Type 2 meronts were found in histological sections of 11 (23.4%) of 47, bone marrow of 11, spleens of 2 but not in muscles and other visceral tissues. *H. canis* DNA was also detected in muscular tissues of 12**	[141]
Serbia	14 localities	2010–2016	T	PCR	79/129 (61.2)	*H. canis* DNA detected in spleens; co-infections with *Babesia* in 27	[485]
Slovakia	Michalovce	NS	S	PCR	4/9 (44.4)	*H. canis* DNA from heart tissue in 3, and skeletal muscle in 1	[486]
Slovakia	Žilina, Prešov, Košice	NS	S	PCR	51/297 (17.1)	*H. canis* DNA from spleens. Sequences deposited	[283]
Spain	Burgos	2006–2007	T, Eu	PCR	2/5 (40.0)	*H. canis* DNA in blood	[487]
Spain	Madrid	2014–2016	T	PCR	3/3 (100)	*H. canis* DNA	[488]
Spain	Guadalajara	NS	S	PCR	18/20 (90.0)	*Hepatozoon* DNA from spleens. Genotypes of *H. canis* characterized molecularly	[145, 292]
Spain	Mediterranean	2011–2018	Dead	PCR	81/89 (91.0)	*H. canis* DNA	[489]
Tunisia	NS	2003–2011	NS	PCR	4/4 (100.0)	*Hepatozoon* sp. DNA	[12]
Turkey	Ankara	2013–2016	S	PCR	3/4 (75.0)	*H. canis* DNA in blood also recovered from pools of *Haemaphysalis parva*	[490]
USA	West Virginia	2011	Eu	HE, PCR	1/1 (100)	*Hepatozoon* DNA in liver and spleen. Meronts and gamonts in sections of mesenteric lymph nodes and spleen. Concomitant *Angiostrongylus vasorum* and *Eucoleus aerophilus* infections in lungs	[491]
USA	Georgia (GA), Louisiana (LA), Maryland (MD), North Carolina (NC), Pennsylvania (PA), Tennessee (TN), Virginia (VA)	2019–2023	S	PCR	21/98 (21.4)	*H. canis* DNA*.* LA-1/2, PA-17/82, TN-1/5, VA-2/2	[492]
Iberian fox (*Vulpes vulpes silacea*)							
Portugal	Lisbon, Alcácer do Sal	1979–1985	S	C, HE	143/301 (47.5)	***H. canis-*like gamonts in blood and tissues of 131 of 138 spleens, 33 of 56 bone marrow, 7 of 17 lymph nodes, and 44 of 125 blood samples**	[123]
Ezo red fox (*Vulpes vulpes schrencki*)							
Japan	Hokkaido	1980	Live	C	1/1 (100)	*Hepatozoon* gamonts in blood*.* Fox comatose, neutrophilia in blood. Epileptic convulsions. Recovered. First report of *Hepatozoon* infection in Japan	[142]
Gray fox (*Urocyon cinereoargenteus*)							
USA	Georgia	NS	NS	PCR	1/1 (100)	*Hepatozoon* DNA from whole blood	[52]
South American gray fox (*Lycalopex griseus*)							
Chile	Six areas	2015–2019	T	PCR	26/80 (32.5)	***H. americanum* in 2 and *H. canis* in 1**	[83]
Hoary fox *(Pseudalopex vetulus)*							
Brazil	São Paulo, Mato Grosso, Federal District		T	PCR	1/8 (12.5)	*H. canis* DNA in fox from Ribeirão Preto	[493]
Brazil	Brasília	2020–2022	T	C, PCR	1/2 (50.0)	Gamont and DNA in blood	[94]
Brazil	Minas Gerais, Goiás	NS	T	PCR	1/5 (20.0)	*Hepatozoon* DNA in blood	[494]
Fennec fox (*Vulpes zerda*)							
Mauritania	NS	2003–2011	NS	PCR	3/4 (75.0)	*Hepatozoon* sp. DNA	[12]
Morocco	NS	2003–2011	NS	PCR	1/3 (33.3)	*Hepatozoon* sp. DNA	[12]
Western Sahara	NS	2003–2011	NS	PCR	3/4 (75.0)	*Hepatozoon* sp. DNA	[12]
Pale fox (*Vulpes pallida*)							
Mauritania	NS	2003–2011	NS	PCR	10/22 (45.4)	*Hepatozoon* sp. DNA	[12]
Niger	NS	2003–2011	NS	PCR	3/5 (60.0)	*Hepatozoon* sp. DNA	[12]
Senegal	NS	2003–2011	NS	PCR	1/1 (100)	*Hepatozoon* sp. DNA	[12]
Rüppel’s fox (*Vulpes rueppellii*)							
Mauritania	NS	2003–2011	NS	PCR	5/6 (83.3)	*Hepatozoon* sp. DNA in 5 of 6	[12]
Morocco	NS	2003–2011	NS	PCR	1/4 (25.0)	*Hepatozoon* sp. DNA	[12]
Cape fox (*Vulpes chama*)							
South Africa	Free State	NS	R	PCR	1/1 (100)	*H. canis-*like in ethanol preserved tissues	[495]
Pampas fox (*Lycalopex* or *Pseudalopex gymnocercus*)							
Argentina	Patagonia	2008	Eu	HE, PCR	1/1 (100)	Clinical, concurrent infection with distemper virus. **Sequences like *H. felis* but lesions like* H. americanum*** (see text)	[139]
Brazil	Santa Catarina	2014	Eu	PCR	1/1 (100)	*H. canis* DNA in blood; concurrent infection with *Rangelia vitalii*	[496]
Brazil	Rio Grande do Sul	NS	S	PCR	5/5 (100)	*Hepatozoon* DNA from spleen	[145]
Uruguay	14 departments	2015–2020	R	PCR	2/32 (6.2)	***H. americanum* identified**	[497]
Andean fox (*Lycalopex culpaeus*)							
Chile	Six areas		T	PCR	70/145 (48.3)	*H. felis* sequences	[83]
Crab-eating fox (*Cerdocyon thous*)							
Brazil	São Paulo, Mato Grosso, and Federal District	NS	T	PCR	1/40 (2.5)	*Hepatozoon* sp. DNA from fox from Piracicaba	[493]
Brazil	Mato Grasso do Sul	2013–2015	T	C, PCR	71/78 (91.0)	*Hepatozoon* sp. DNA found in whole blood; gamonts not found	[185]
Brazil	Mato Grasso do Sul	2018–2023	Dead, R	PCR	3/5 (60.0)	DNA in spleen	[188]
Brazil	São Paulo	1995	Eu	C	1/1 (100)	Anemia. *H. canis* gamonts in neutrophils	[498]
Brazil	Rio Grande do Sul	NS	S	PCR	6/8 (75.0)	*H. canis* DNA in spleen of 5. *H. americanum*-like DNA in spleen of 1	[145]
Brazil	ES-060 highway	2004–2009	R	C, PCR	29/58 (50.0)	*Hepatozoon* DNA in blood and tissues; DNA from **1 fox close *to H. americanum*,** and other **close to a reptile-associated *Hepatozoon***	[11]
Brazil	Mato Grosso do Sul	2018	T	PCR	11/12 (91.6)	*Hepatozoon* DNA from blood. **Sequences closely matched *H. americanum***	[499]
Brazil	Pantanal	NS	NS	PCR	10/78 (12.8)	*Hepatozoon* sp. DNA in blood	[500]
Uruguay	14 departments	2015–2020	R	PCR	6/45 (13.3)	***H. americanum* identified**	[497]
JACKALS							
Silver-backed jackal (*Canis mesomelas*)							
East Africa	NS	NS	NS	C	NS	Gamont in blood	[501]
South Africa	Kruger National Park	NS	S	HE	3/3 (100)	Severe myositis, type 2 meronts in muscles and visceral tissues of 3	[419]
South Africa	Northwest Province, Gauteng	NS	T	PCR	20/91 (21.9)	14 were confirmed *H. canis based on* sequencing. 2 samples had different *Hepatozoon* genotypes	
Side striped jackal (*Canis adustus*)							
East Africa	NS	NS	NS	C	NS	Gamont in blood smears	[501]
Ethiopia	NS	2003–2011	R	PCR	1/1 (100)	*Hepatozoon* sp. DNA from tissue	[12]
Tunisia	Tunis	NS		C	1/1 (100)	Gamonts in blood leukocytes. **Meronts in bone marrow, spleen, and liver**	[502]
Golden jackal (*Canis aureus*)							
Algeria	NS	2003–2011	R	PCR	2/5 (40.0)	*Hepatozoon* sp. DNA from tissue	[12]
Austria	Vienna	2012	R	PCR	1/1 (100)	*H. canis* DNA in blood, liver, spleen*.* **Cloning and sequencing revealed 6 haplotypes***.* No lesions or meronts	[503]
Austria	Wiener Neudorf	2013–2015	R	PCR	1/1 (100)	*H. canis* DNA in 1 of 1 jackal	[504]
Croatia	NS	NS	NS	PCR	14/46 (30.4)	*Hepatozoon* PCR of liver or skeletal muscle	[124]
Croatia	NS	NS	Dead	PCR	21/26 (80.8)	*H. canis* DNA	[66]
Czech Republic	Moravia-Silesia	2013–2015	S	PCR	1/1 (100)	*H. canis* DNA	[504]
Hungary	NS	NS	NS	PCR	33/57 (57.8)	*Hepatozoon* in PCR of liver or skeletal muscle	[124]
Hungary	Southern Transdanubia	NS	S	PCR	9/15 (60.0)	*H. canis* DNA in blood, sequences deposited	[479]
Israel	Multiple regions	NS	Live	C	1/46 (1.5)	*H. canis* gamonts in blood smear	[505]
Israel	Throughout	NS	T	PCR	50/109 (45.8)	*H. canis* DNA from blood and spleen	
Mauritania	NS	2003–2011	R	PCR	4/16 (25.0)	*Hepatozoon* sp. DNA from tissue	[12]
Montenegro	NS		NS	PCR	2/2 (100)	*Hepatozoon* PCR of liver or skeletal muscle	[124]
Romania	11 counties	2013–2015	S	PCR	39/54 (72.2)	*H. canis* DNA. 7 co-infected with *Babesia* sp.	[504]
Serbia	NS	NS	NS	PCR	139/206 (67.4)	*Hepatozoon* in PCR of liver or skeletal muscle	[124]
Serbia	23 localities	2010–2020	S	PCR	**90/114 (78.9)**	*H. canis* DNA in spleens. Data for each region sequenced	[506]
Indian jackal (*Canis aureus indicus*)							
India	Maharashtra	NS	Live, automobile accident	C, PCR	1/1 (100)	**Fever, anemia, depression. Gamonts in neutrophils in blood. *H. canis* PCR-positive. Treated successfully with doxycycline**	[507]
WOLVES							
Gray wolf (*Canis lupus*)							
Bosnia	NS	NS	S	PCR	1/1 (100)	*H. canis* DNA	[66]
Croatia	NS	NS	S	PCR	65/120 (54.2)	*H. canis* DNA	[66]
Czech Republic	Several areas	2014–2021	Different sources	PCR	10/10 (100.0)	*H. canis* DNA	[508]
Germany	11 Federal states	2006–2019	Different sources	PCR	127/276 (46.0)	*H. canis* DNA from spleens	[509]
Italy	Piedmont	2009–2017	R	PCR	25/33 (75.7)	*Hepatozoon* sp. DNA from tissues	[483]
Netherlands	NS	NS	R	PCR	8/8 (100)	*H. canis*	[66]
Poland	13 regions	2016–2022	Different sources	PCR	21/60 (35.0)	*H. canis* DNA from blood	[275]
Serbia	30 localities	2010–2019	S	PCR	62/107 (57.9)	*H. canis* DNA in spleens. Data for each region tabulated. 36 PCR-positives sequenced	[510]
Iberian wolf (*Canis lupus signatus*)							
Spain	Cantabria	2022	S, R	HE, PCR	**78/81 (96.3)**	Co-infections with *Leishmania infantum* in 20, *B. canis* in 3. Type 2 meronts in spleen of a wolf co-infected with *B. canis*	[511]
Maned wolf (*Cerdocyon brachyurus*)							
Brazil	São Paulo, Mato Grosso, and Federal District	NS	Z	PCR	1/22 (4.5)	*H. canis* DNA from blood from wolf from Sorocaba	[493]
Brazil	Minas Gerais	NS	T	PCR	21/37 (56.7)	*Hepatozoon* DNA in blood**. 2 haplotypes, 1 aligned with *H. canis* and 1 aligned with *H. americanum***	[512]
Brazil	Minas Gerais, Goiás	NS	T	PCR	1/6 (16.6)	*Hepatozoon* sp. DNA in blood	[494]
WILD DOGS							
African wild dog (*Lycaon pictus*)							
South Africa	Kruger National Park	1990–1991	T	C	26/29 (89.6)	Gamonts in ear tip blood smears. Co-infection with *Babesia* sp*.* in 2 dogs	[513]
South Africa	National Parks, Game Reserves	NS	T, P	PCR	2/301 (0.7)	*Hepatozoon* DNA in blood in dogs from De Wildt Cheetah and Wildlife Centre	[514]
Tanzania	Serengeti	2009–2011	Live, T	C	13/16 (81.5)	Gamonts in blood smears. Co-infection with *Babesia* in 1 and microfilariae in another	[515]
Zambia	National Parks	NS	P	PCR	12/22 (54.5)	*H. canis*-like sequences	[129]
Indian/Asiatic wild dog (*Cuon alpinus*)							
India	Hyderabad	2007–2010	Z	PCR	2/2 (100.0)	*H. canis* DNA in blood	[228]
Thailand	Northeastern	2018	Dead	PCR	1/1 (100)	*H. canis* DNA from heart and spleen. Sequence deposited. Co-infected with *Babesia*	[516]
Bush dog (*Speothos venaticus*)							
Brazil	São Paulo, Mato Grosso, Federal District	NS	Z	PCR	2/27 (7.4)	*H. canis* DNA; 1 from São Paulo, 1 from Cuiabá	[493]
Brazil	Mato Grosso do Sul	2018–2023	Wild, R	PCR	1/1 (100)	*Hepatozoon* DNA in spleen	[188]
COYOTES (*Canis latrans*)							
USA	Oklahoma	NS	S	HE	9/16 (56.2)	Myositis with type 2 skin onion meronts of *H. americanum*	[125]
USA	Oklahoma	1998	S	HE	8/20 (40.0)	Myositis with onion skin type 2 meronts of *H. americanum.* **No bone lesions**	[126]
USA	Oklahoma (2 coyotes), Texas (1 coyote)	NS	NS	PCR	3/3 (100)	*Hepatozoon* DNA from whole blood of 3	[52]
USA	Oklahoma, Texas	NS	S	C, HE, PCR	35/44 (79.5)	*H. americanum*, *H. canis*, and unidentified *Hepatozoon* species by PCR	[150]
USA	Texas	1977	S	HE	1/1 (100)	Type 2 meronts in heart	[517]
USA	Texas	NS	T	HE	12/59 (20.3)	Meronts in histological sections	[518]
USA	Texas	NS	NS	PCR	2/2 (100)	*Hepatozoon* DNA from whole blood	[52]
USA	Louisiana, Maryland, North Carolina, Pennsylvania, South Carolina, Tennessee, Virginia	NS	Different sources	PCR	38/180 (21.1)	*H. canis* 13 (7.2%), ***H. americanum* 26 (14.4%)**, **1 coyote co-infected with both species. Data provided for each state and both *Hepatozoon* species separately**	[492]

*TEM* transmission electron microscopy,

*NS* not stated

^a^*Eu* euthanized, *P* park reserve, *R* road death, *S* shot or hunted, *T* trapped or caught, *Z* zoo

^b^*C* cytology, stained blood smears, *ELISA* enzyme-linked immunosorbent assay, *HE* hematoxylin and eosin-stained histological sections, *PCR* *18S* rRNA

Information in bold = important

**Table 8:** *Hepatozoon* infections in wild felids

Country	Region, locality	Year sampled	Source^a^	Diagnostic method^b^	No. positive/no. tested (% positive)	Findings, including *Hepatozoon* spp. identified	References
LIONS							
Lion (*Panthera leo*)							
East Africa	NS	NS	NS	C	1/1 (100)	Gamonts in blood	[501]
Kenya	Muguga		S	C, HE	1/1 (100)	Meronts in heart. Gamonts in blood smear	[519]
Kenya	Nairobi National Park	1989 or earlier	1 Eu	HE	2/2 (100)	Type 2 meronts in histological sections of myocardium	[520]
South Africa	Kruger National Park		Dead	HE	4/4 (100)	Type 2 *Hepatozoon* meronts in muscles, heart, blood vessels	[419]
Tanzania	Serengeti National Park		NS	C	27/56 (48.2)	Gamonts (with a hook) in blood	[521]
Tanzania	Serengeti National Park and Ngorongoro Crater	1985	T	C	**123/123 (100)**	Gamonts in blood smears. **A single type 2 meront in sections of heart of 1**	[127]
Zambia	National Parks	2009–2011	P	PCR	23/48 (47.9)	***H. canis* and *H. felis*-like sequences**	[129]
Zimbabwe	Gweru, Masvingo, Dollar Block	NS	T	PCR	11/86 (12.7)	***H. canis* DNA in 2**, and ***H. felis* DNA in 9** from blood	[522]
Asiatic lion (*Panthera leo persica*)							
India	Hyderabad	2007–2010	Z	PCR	5/9 (55.5)	*H. felis* DNA in whole blood	[228]
India	Uttar Pradesh (Etawah)	NS	Lion safari	PCR	1/5 (20.0)	*H. felis* DNA in blood	[523]
India	Gir National Park		P	C, PCR	**77/77(100)**	*H. felis* PCR (*18S* rRNA) in blood, and gamonts in blood of 4. **Two genotypes (HfG1 and HfG2) identified**	[128]
TIGERS							
Tiger (*Panthera tigris)*							
Italy	Veneto, Lazio, Piedmont	2019	Z	PCR	2/4 (50.0)	One positive for *H. felis* DNA and 1 positive for *H. silvestris* DNA	[153]
Italy	Brindisi		P	PCR	3/20 (15.0)	***H. canis* DNA in blood**	[524]
Bengal tiger (*Panthera tigris tigris)*							
India	Hyderabad	2007–2010	Z	PCR	2/5 (40.0)	*H. felis* DNA in blood	[228]
India	Nagpur	2018	Z	C, PCR	1/1 (100)	Gamonts in neutrophils in blood. *H. felis* DNA in blood, sequenced	[525]
Thailand	Western	2019	T	PCR	2/17 (11.7)	**One *H. canis*, 1 *H. felis.*** Co-infection with *E. canis* in 1	[526]
LEOPARDS							
Leopard (*Panthera pardus*)							
South Africa	Kruger National Park		Dead	HE	1/1 (100)	*Hepatozoon* meronts in heart and skeletal muscle	[419]
South Africa	Various localities	NS	Z (9 captive, 8 wild)	C, PCR	8/17 (47.0)	Two new species of *Hepatozoon*, *H. luiperdjie* and *H.ingwe* described (see text)	[48]
Kenya	Naivasha	NS	NS	C	NS	Gamonts in blood	[519]
East Africa	NS	NS	NS	C	NS	Gamont in blood	[501]
Indian leopard (*Panthera pardus fusca*)							
India	Hyderabad	2007–2010	T	PCR	2/4 (50.0)	*H. felis* DNA in whole blood	[228]
Persian leopard (*Panthera pardus ciscaucasica*)							
Iran	North Khorasan	2011	T	C	1/1 (100)	Cachectic, dehydrated leopard. Gamonts in blood smear. Improved clinically after treatment with antibiotics	[527]
CHEETAH (*Acinonyx jubatus*)							
South Africa	Hluhluwe Game Reserve	NS	Dead	HE	1/1 (100)	Meronts in heart, skeletal muscle, adipose tissue	[65]
South Africa	Kruger National Park	1985	Dead	HE	2/2 (100)	Type 2 meronts in adipose tissue, heart, blood vessels of 2	[419]
Tanzania	Serengeti National Park and Ngorongoro Crater		T	C	8/8 (100)	Gamonts in blood smears	[127]
Tanzania	Serengeti		T	C	13/24 (54.2)	Gamonts in blood smears. Resampling indicated persistence of parasitemia up to 14 months	[515]
JAGUAR (*Panthera onca*)							
Brazil	Mato Grosso do Sul	2018–2023	Dead, R	PCR	2/2 (100)	*Hepatozoon* spp. DNA in spleen	[188]
Venezuela	Los Llanos	NS	T	PCR	1/2 (50.0)	*H. canis* DNA in blood	[528]
LYNXES							
Eurasian lynx *(Lynx lynx)*							
Czech Republic	Several areas		Several sources	PCR	2/17 (11.8)	***H. silvestris* DNA**	[508]
Bobcat (*Lynx rufus*)							
USA	California	1980	T	C	2/2 (100)	*Hepatozoon* sp. gamonts in blood	[529]
USA	Georgia		NS	PCR	1/1 (100)	*Hepatozoon* DNA from spleen	[52]
USA	Mississippi	2017	S	HE	11/25 (44.0)	Meronts of *H. rufi* in muscle*s*	[157]
USA	Oklahoma	2018–2019	S	HE	2/56 (3.5)	Meronts of *H. rufi* in tongue	[530]
USA	Texas	NS	T	C	3/20 (15.0)	*Hepatozoon* sp. gamonts in blood	[518]
PUMA (*Puma concolor*)							
Brazil	São Paulo, Mato Grosso, Federal District		Z	PCR	1/18 (5.5)	*H. felis*-like DNA in blood. From São Paulo	[493]
Brazil	Minas Gerais, Goiás	NS	T	PCR	1/2 (50.0)	*Hepatozoon* sp. DNA in blood	[494]
SOUTH AMERICAN FELIDS							
Ocelot (*Leopardus pardalis*)							
Brazil	Minas Gerais, Goiás	NS	T	PCR	2/3 (75.0)	*Hepatozoon* sp. DNA in blood	[494]
Brazil	Mato Grosso do Sul	2003–2011	T	C, PCR	5/7 (71.4)	*Hepatozoon* sp. DNA found in whole blood. No gamonts in blood	[185]
Brazil	Pará	2009–2011	R	PCR	1/1 (100)	*H. felis* DNA in blood	[531]
Brazil	Mato Grosso do Sul	2018–2023	Dead, R	PCR	1/2 (50.0)	DNA in blood	[188]
Brazil	Maranhão, Piauí, Ceará	NS	T, Z	C, PCR	4/10 (40.0)	*Hepatozoon* sp. gamont in blood of one ocelot from Maranhão. *Hepatozoon* sp DNA in blood of 4	[532]
USA	Texas	NS	T	C	6/13 (46.1)	*Hepatozoon* sp. gamonts in blood smear	[518]
Little spotted cat (*Leopardus tigrinus*)							
Brazil	São Paulo, Mato Grosso, Federal District	NS	Z	PCR	4/39 (10.2)	*H. felis*-like DNA in blood. 3 from Jundiaí, 1 from Brasília	[493]
Brazil	Maranhão, Piauí, Ceará	NS	Z	C, PCR	1/10 (10.0)	*Hepatozoon* sp. DNA in blood	[532]
Jaguarundi (*Puma yagouaroundi*)							
Brazil	São Paulo, Mato Grosso, Federal District		Z	PCR	1/25 (4.0)	*H. felis*-like DNA in blood. From Ilha Solteira Zoo	[493]
Brazil	Mato Grosso do Sul	2018–2023	Dead, R	PCR	1/3 (33.3)	DNA in blood	[188]
EUROPEAN WILDCAT (*Felis silvestris silvestris*)							
Bosnia and Herzegovina	Five locations	2011–2016	S	C, HE, PCR	6/9 (66.6)	*Hepatozoon silvestris* proposed (see Table 1). Meronts in tissues of 4; *H. silvestris* DNA in 5; *H. felis* DNA in 1	[46]
Bosnia and Herzegovina	Eight locations	2011–2017	Dead, S	HE, PCR	7/9 (77.8)	Two cats *H. silvestris*, 3 cats *H. felis;* 2 cats had undetermined *Hepatozoon* sp.	[533]
Germany	Six districts	1998–2020	Dead, R	PCR	40/96 (35.4)	*H. felis* DNA in 6 and *H. silvestris* DNA in 34. Co-infection with other pathogens	[534]
Greece	Xanthi	NS	T	C	1/1 (100)	*H. felis* gamont in neutrophils in a sick cat; concurrent infections with other pathogens. Released to wilderness	[535]
Hungary	Aggtelek National Park	NS	Dead	PCR	3/4 (75.0)	*H. felis* in 2 and co-infection with *Cytauxzoon europaeus* in the third cat	[536]
Hungary	Aggtelek National Park	NS	R	PCR	3/4 (75.0)	*H. felis* DNA	[452]
Italy	Friuli-Venezia Giulia		T	PCR	8/19 (42.1)	Six *H. felis*, 2 *H. silvestris*	[153]
Spain	Northern	NS	R	PCR	29/63 (46.0)	*H. felis* DNA in spleens of all; **no *H. silvestris***	[537]
Spain	South	2011–2018	Dead	PCR	4/5 (80.0)	*H. felis* in 3, **H. martis in 1**	[489]
LEOPARD CAT (*Prionailurus bengalensis*)							
Korea	Seoul	NS	Dead, R	HE, PCR	4/5 (80.0)	*Hepatozoon* identified in 4 of 5 hearts; microscopically in 3, and by PCR in 1. Type 2 meronts. DNA similar to *H. felis*	[538]
Thailand	Chonburi	NS	Z	C, PCR	1/13 (7.6)	The infected cat had been caught wild and had persistent low-level parasitemia for 1 year. No other blood abnormality. *Hepatozoon* DNA characterized molecularly; 12 other zoo cats were negative	[539]
IRIOMOTE CAT (*Felis iriomotensis*)							
Japan	Nagasaki	1993–2005	R	C, HE, TEM	17/30 (56.7)	Heart was infected in all 17 cats. Type 2 meronts in muscles of tongue of 4, masseter of 2, diaphragm of 1, thigh of 1, but not in other organs including blood. TEM of meronts and merozoites. Gamonts not detected	[540]
Japan	NS	2011	T	C, PCR	1/1 (100)	The infected cat was caught 3 times in 2 years and evaluated hematologically. *Hepatozoon* gamonts were found in neutrophils in peripheral blood smears. PCR revealed *H. felis* DNA. No other blood abnormality. Cat was FIV- and FELV-negative	[541]
Japan	NS	2002–2012	Dead, T	C, PCR	31/43 (72.0)	Gamonts in blood smears of 24. ***H. felis* DNA in 31**	[542]
TSUSHIMA LEOPARD CAT (*Felis bengalensis euptilura*)							
Japan	Nagasaki	1993–2005	R	C, HE, TEM	6/42 (14.3)	Only heart was parasitized*.* Development of *Hepatozoon* meronts in sections of hearts described. Meronts and merozoites were PAS-positive. TEM confirmed the presence of merozoites in meronts. Gamonts not detected	[540]
Japan	NS	2002–2012	Dead, T	C, PCR	14/14 (100.0)	Gamonts in blood smears of none. ***H. felis* DNA**	[542]
FLAT-HEADED CAT (*Prionailurus planiceps*)							
Thailand	Mahidol University	NS	T	C, PCR, TEM	2/2 (100.0)	Gamonts in blood. TEM of infected blood neutrophils, *18S* rRNA—650-base-pair sequences suggested similarity to *Hepatozoon* of frog	[543]
PALLAS’S CAT (*Felis manul*)							
USA	Chicago Zoo, imported from Moscow Zoo, Russia		Z	C	1/4 (25.0)	Gamonts in leukocytes	[544]

*NS* not stated

^a^*Eu* euthanized, *P* park reserve, *R* road death, *S* shot or hunted, *T* trapped or caught, *Z* zoo

^b^*C* cytology, stained blood smears, *HE* hematoxylin and eosin-stained histological sections, *PCR* *18S* rRNA, *TEM* transmission electron microscopy

Information in bold = important

**Table 9:** *Hepatozoon* infections in miscellaneous wild Carnivora

Country	Region, locality	Year sampled	Source^a^	Diagnostic method^b^	No. positive/no. tested (%)	Findings	References
SPOTTED HYENA (*Crocuta crocuta*)							
East Africa	NS	NS	NS	C	NS	Gamonts in blood	[487]
Mali	NS	NS	NS	C	1/6 (16.6)	Gamonts in blood	[545]
South Africa	Zululand and Hluhluwe Game Reserve	NS	S	HE	2/2 (100)	Both hyenas in good condition. Meronts in sections of **tongue and heart** of the hyena from Zululand and in **lungs and external genitalia** of the female hyena from Hluhluwe	[65]
South Africa	Kruger National Park	NS	S	HE	8/8 (100)	Vascular “trophozoites,” “tissue cyst-like (24 × 4.8 µm with single zoite)” in lymph node, type 2 meronts in muscle and visceral tissues, more prominent in muscles (see text)	[419]
Tanzania	Serengeti National Park	1997–2004	NS	C	4/9 (44.4)	Gamonts in blood	[521]
Tanzania	Serengeti National Park	NS	Died, natural causes	HE, PCR	11/11 (100)	*Hepatozoon* infections in 11 young hyenas (see text). **DNA characterization from 5 hyenas suggested *H. felis*, instead of *H. canis***	[131]
Zambia	National Parks	2009–2011	P	PCR	18/38 (47.3)	***H. canis* and *H. felis*-like sequences**	[129]
PROCYONIDS							
Raccoon (*Procyon lotor*)							
Czech Republic	Several areas	2014–2021	Several sources	PCR	**1/35 (2.9)** **3/35 (8.6)**	***H. canis*** ***Hepatozoon sp.***	[508]
Spain	Madrid	2014–2016	T	PCR	5/194 (2.5)	*H. canis* DNA	[488]
USA	Georgia	NS	T	C, HE	6/8 75.0)	*H. procyonis* proposed (see text)	[50]
USA	Oklahoma		NS	PCR	4/4 (100)	*Hepatozoon* DNA from heart	[52]
USA	Texas	NS	Dead	C, HE	57/65 (87.6)	Gamonts in blood, granulomas in heart. Type 2 meronts in tissues (see text)	[546]
USA	Texas		T	PCR	5/15 (33.3)	*Hepatozoon* sp. DNA in heart and spleen tissues of 5 out of 15	[521]
USA	Pennsylvania (6 raccoons), Tennessee (117 raccoons)	NS	S	HE, PCR	3/123 (2.4)	*Hepatozoon* DNA in all 3. Type 2 meronts associated with myocarditis in 1 raccoon. All 6 raccoons from Pennsylvania were negative	[547]
Crab-eating raccoon (*Procyon cancrivorus*)							
Brazil	Rio de Janeiro	2002–2004	T	C	2/20 (10.0)	*H. procyonis* gamonts	[54]
Brazil	Mato Grasso do Sul	2018–2023	Dead, R	PCR	1/2 (50.0)	DNA in spleen	[188]
Venezuela	Los Llanos	NS	Live	PCR	1/4 (25.0)	*H. canis* DNA in blood	[528]
Panamanian crab-eating raccoon (*Procyon cancrivorus panamensis*)							
Panama	Pacora	1965	T, S	HE	1/1 (100)	*Hepatozoon* meronts in heart (see text)	[55]
Ring-tailed coati (*Nasua nasua*)							
Brazil	Mato Grosso do Sul	NS	T	C, PCR	112/165 (67.8)	*H. procyonis* gamonts in blood of 67 (40.6%) and DNA in 112 (68%)	[130]
Brazil	Rio de Janeiro	2002–2004	T	C	2/20 (10.0)	*Hepatozoon* gamonts in blood	[54]
Brazil	São Paulo	2013–2017	T	C, PCR	21/83 (25.3)	Gamonts in 9 and DNA in 21; redescription of *H. procyonis* (see text)	[53]
Brazil	Mato Grosso do Sul	2003–2011	T	C, PCR	13/31 (41.9)	No gamonts in blood. *Hepatozoon* DNA in blood of 13	[185]
Brazil	Mato Grosso do Sul	2018–2023	Dead, R	PCR	3/4 (75.0)	*Hepatozoon* DNA in blood	[188]
MUSTELIDS							
European pine marten (*Martes martes*)							
Netherlands	NS	NS	Dead	PCR	37/50 (74.0)	*H. canis* DNA	[66]
Spain	Burgos		S	HE, PCR	3/3 (100)	*Hepatozoon* sp. DNA in liver or spleen. Parasites not seen in histological sections	[528]
Bosnia and Herzegovina	Several locations	2014–2017	T	C, HE, PCR	10/10 (100)	*H. martis* described (see Table 1)	[45]
Germany	Bavaria	NS	R	HE	6 tested	See text	[135]
Hungary	Aggtelek National Park, South		Dead	PCR	4/4 (100.0)	*H. martis* in 3 and *Hepatozoon* sp. in 1	[536]
Scotland	Several locations	NS	R	HE	3/4 (75.0)	Severe hepatozoonosis in 3 of 4 dead martens (see text)	[133]
Stone marten (*Martes foina*)							
Croatia	Continental, North and Western	2014–2017	T	C, HE, PCR	42/66 (63.6)	*H. martis* described (see Table 1)	[45]
Croatia	NS	NS	Dead	PCR	42/66 (63.6)	*H. canis* DNA	[66]
Czech Republic	Several areas		Several sources	PCR	1/2 (50.0)	*Hepatozoon* sp.	[508]
Germany	Bavaria	NS	T	HE	489 tested. 107 positive (56.6%) of hearts, 25 (27.0%) of skeletal muscles, 17 (25%) of tongues, and in 3 (1.3%) of 236 lungs. Total positive is not stated	See text	[135]
Hungary	Aggtelek National Park		Dead	PCR	2/3 (66.6)	*H. martis* in 1 and *Hepatozoon* sp. in the second	[536]
Netherlands, Belgium	NS	NS	Dead	PCR	32/67 (47.8)	*H. canis* DNA	[66
Spain	South	2011–2018	Dead	PCR	15/16 (93.7)	*H. canis* DNA in 2 and *H. martis* in 13	[489]
Martens (*Martes* spp.)							
Switzerland	Several areas		Dead	HE	3/275 (0.1)	Includes stone and pine martens	[548]
Japanese marten (*Martes melampus*)							
Japan	Gifu	1991–1992	S	HE	67/70 (96)	*Hepatozoon* meronts in tissues (see text)	[132]
Japan	Akita, Gifu, Shiga	NS	S	HE, PCR	12/14 (85.7)	Detailed description of *Hepatozoon* granulomas in the myocardium. Granulomas contained intact meronts as well as individual zoites. Type 2 meronts were relatively rare. Several uninucleate meronts (authors called them trophozoites) were present. DNA sequences suggested that the parasite was similar to that in pine martens	[138]
Yellow-throated marten (*Martes flavigula koreana*)							
Korea	Iksan	NS	Dead	C, HE	1/1 (100)	Co-infection with distemper virus. Granulomatous lesions with *Hepatozoon* meronts (see text)	[134]
Mink (*Mustela vison*)							
Canada	Ontario	NS	T	HE	10/18 (55.6)	*Hepatozoon* meronts in lungs (see text)	[136]
USA	Pennsylvania	2015–2016	Dead	PCR	7/10 (70.0)	*Hepatozoon* sp*.* DNA	[547]
USA	Pennsylvania	2023	Dead	HE, PCR	3/3 (100)	Type 2 meronts associated with pneumonitis in case 1, incidental in heart and lungs of case 2, and lungs of case 3 concurrent with distemper virus infection (see text)	[137]
European badger (*Meles meles*)							
Croatia	NS	NS	Dead	PCR	6/64 (9.4)	*H. canis*	[66]
Hungary	Somogy, Baranya	2020–2021	R	PCR	1/38 (2.6)	*Hepatozoon* sp. DNA in spleen	[549]
Spain	Asturias, Basque Country	NS	S	PCR	1/122 (0.8)	*Hepatozoon* sp. DNA in spleen. Co-infection with *Cystoisospora* sp.	[550]
Spain	South	2011–2018	S	PCR	3/24 (12.5)	*H. martis* DNA	[489]
Switzerland	Several areas		Dead	HE	1/249 (0.04)		[548]
European polecat (*Martes putorius*)							
Netherlands, Belgium	NS	NS	Dead	PCR	10/100 (10.0)	*H. canis* DNA	[66]
Siberian polecat (*Mustela eversmanii satunini*)							
Russia (imported into USA)	NS		Live	HE		Disseminated hepatozoonosis outbreak (see text)	[56]
Stoat (*Mustela erminea*)							
Germany	Bavaria	NS	R	HE	16 examined	See text	[135]
Least weasel (*Mustela nivalis*)							
Hungary	Aggtelek National Park		Dead	PCR	1/2 (50.0)	*Hepatozoon* sp. DNA in tissues	[536]
Tayra (*Eira barbara*)							
Brazil	Minas Gerais, Goiás		T	PCR	1/1 (100)	*Hepatozoon* sp. DNA in blood	[494]
Neotropical otter (*Lontra longicaudis*)							
Brazil	Mato Grosso do Sul		Dead, R	PCR	3/4 (75.0)	*Hepatozoon* DNA in blood	[188]
BEARS							
Japanese black bears *(Ursus thibetanus japonicus)*							
Japan	Iwate Prefecture	NS	NS	PCR	119/156 (76.3)	*H. ursi* DNA in liver or blood	[551]
Japan	Fukui, Shiga, Gifu	1991–1992	S	HE, TEM	18/18 (100)	*Hepatozoon* found in lungs of all 18 bears. Structures of meronts, merozoites, and gamonts described	[158]
Japan	Gifu	NS	S	C, PCR, TEM	35/35 (100)	*H. ursi* described (see Table 1)	[47]
Japan	Hokkaido, Tochigi, Nagano		S	PCR	82/133 (61.6))	0/49 Hokkaido, 18/18 Tochigi, 64/66 Nagano	[552]
Brown bear (*Ursus arctos)*							
Turkey	Erzurum		Dead	C, PCR	2/2 (100.0)	*H. ursi* gamonts in both bears. DNA sequences were deposited	[553]
Indian sloth bear (*Melursus ursinus*)							
India	Agra, Bengaluru, Bhopal	2007–2010	T	PCR	38/54 (70.0)	*H. ursi* DNA	[554]
Giant panda (*Ailuropoda melanoleuca*)							
USA, China, UK	Various zoos	2005	Z	C, PCR	14/14 (100)	Gamonts, novel genotype based on *18S* rRNA	[555]
VIVERRIDS							
Palm civet (*Paradoxurus hermaphroditus*)							
Malaysia	Jeram		NS	C	1/34 (2.9)	Gamonts in blood	[282]
Cape genet* (Genetta tigrina)*							
East Africa	NS	NS	NS	C	NS	Gamont in blood	[501]
Kenya	Nairobi		R	C	NS	Gamont in blood	[519]
Small spotted genet (*Genetta genett*a)							
South Africa	Limpopo		R	PCR	1/1 (100)	*H. felis-*like from ethanol preserved tissues	[495]
WHITE-TAILED MONGOOSE (*Ichneumia albicauda*)							
South Africa	Limpopo province	NS	R	PCR	1/3 (33.3)	*H. felis* DNA from tissues	[556]

^a^*Eu* euthanized, *NS* not stated, *P* park reserve, *R* road death, *S* shot or hunted, *T* trapped or caught, *Z* zoo

^b^*C* cytology, stained blood smears, *HE* hematoxylin and eosin-stained histological sections, *PCR* (*18S* rRNA), *TEM* transmission electron microscopy

Information in bold = important

### Prevalence

#### Prevalence in wild canids

The prevalence of *Hepatozoon* spp. infections in some of the wild canids, particularly red foxes and golden jackals in Europe, is high (Table 7). For example, 47.5% of 301 red foxes in Portugal were infected, as detected by insensitive microscopic examination of blood smears for gamonts [123]. In another survey from Austria, 29.8% of 506 foxes were infected, including a finding of *H. canis* DNA in fetal tissues of foxes [31]. In a survey of 206 golden jackals from Serbia, *H. canis* was detected in 56.9% [124]. These data indicate that jackals and red foxes could be reservoir hosts for *H. canis*. The same may apply to coyotes in the USA as reservoir hosts for *H. americanum* [125, 126].

#### Prevalence in wild felids

High prevalence of *Hepatozoon* spp. has been reported in some of the wild felids (Table 8). Remarkably, 100% prevalence of *Hepatozoon* sp. gamonts was found in blood smears in lions from Tanzania [127] and of *H. felis* DNA in blood of lions in India [128]. Although one would expect *H. felis* to be the most prevalent species, some felids had *H. canis* infection and some were dually infected with both *H. canis* and *H. felis* [129]. While ingestion of the infected ticks is the likely source of infection, carnivorism of tissues infected with tissue cysts in paratenic hosts and transplacental transmission are additional possible routes of transmission.

#### Prevalence in wild miscellaneous Carnivora

Prevalence data are summarized in Table 9.

High prevalence was reported in ring-tailed coatis from Brazil [130] and martens of several species (Table 9).

### Clinical hepatozoonosis in wildlife

Clinical infections in selected species of wild canids are summarized for a better understanding of the pathogenesis and epidemiology of hepatozoonosis in wild canids.

#### Spotted hyenas

An investigation into the causes of mortality in spotted hyena *(Crocuta crotuta)* from the Serengeti National Park, Tanzania, suggested *Hepatozoon* infection as an etiology in the deaths of hyenas [131]. Two cubs (1.3 and 2.2 months old) died and had signs of ataxia and labored breathing. Of 11 hyenas that died, two 1–2-month-old hyenas had ataxia, ocular discharge, and terminal dyspnea. Tissues of eight hyenas were fixed for histopathology. *Hepatozoon* sp. meronts were found in the lungs of all eight hyenas, in some with interstitial pneumonia. Meronts were found in the hearts of five, sometimes with focal mild necrotizing myocarditis. In three hyenas, meronts were found in several organs, including skeletal muscle, stomach, lymph nodes, kidneys, liver, and spleen. Molecular data indicated a close relationship between the *Hepatozoon* sp. found in the hyena and *H. felis* [131].

#### Hepatozoonosis in mustelids

High prevalence of *H. martis* was reported in European pine martens (*Martes martes*) from Bosnia and Herzegovina and stone martens (*Martes foina*) from Croatia found dead and necropsied [45]. There was no description of clinical signs. No macroscopic lesions were found, and the martens appeared to be in good nutritional state based on necropsy data. However, at recent necropsies of several stone martens (*M. foina*), multifocal small white granulomas were observed in the myocardium (unpublished data AA), and mild to severe microscopic lesions were found in the tissues of martens [45]. Multifocal pyogranulomas were found in hearts and skeletal muscle of martens, and some of the pyogranulomas were histologically severe. In a pine marten, granulomas associated with *H. martis* infection were also present in adipose tissue [45].

A very high rate of *Hepatozoon* infections was also documented in Japanese martens (*Martes melampus*) hunted in Japan [132]. The clinical status was unknown, but severe lesions were found. Grayish white areas were visible grossly in the hearts of 6 of 70 martens. *Hepatozoon* stages (type 2 meronts) were found in hearts of 67, perirenal adipose tissue of 25, diaphragms of nine, mesentery of 10, tongue of one, and omenta of eight. Many meronts were enclosed in nodules that ranged up to 400 µm in diameter, especially in the heart. Cardiomyocytes were described as the affected host cells, and developing stages of meronts were visible. The nuclei in immature meronts were located at the periphery or arranged in blastomeres. Granulomas were seen around ruptured meronts and contained merozoites or gamonts, confirmed ultrastructurally [132].

Similar granulomatous myositis was reported in mustelids by others. Granulomatous lesions were seen in the heart of three of four pine martens (*M. martes*) found dead in Scotland [133]. Meronts and merozoites reacted positively to *H. americanum* antibodies in an immunohistochemical reaction [133]. *Hepatozoon*-associated granulomatous lesions were reported in a yellow-throated marten (*Martes flavigula koreana*) from Korea; the animal had concurrent distemper virus infection [134]. Macroscopic white nodules were seen on the surface and parenchyma of the heart and omentum and contained type 2 *Hepatozoon* meronts and individual merozoites/gametes.

An extensive study on hepatozoonosis in mustelids was conducted in Germany by Geisel et al. [135]. A total of 489 stone martens (*M. foina*), six pine martens (*Martes martes*), and 16 stoats (*M. erminea*) were examined. *Hepatozoon*-associated granulomas were found in 107 (56.6%) hearts, 25 (27.0%) skeletal muscles, 17 (25%) tongues, and three (1.3%) of 236 lungs of stone martens. Granulomas were also found in the uteri of one of 26, thyroid gland in one of six, and salivary glands in one of nine stone martens. Of samples from pine martens and stoats, three of 13 hearts and one of nine skeletal muscles were positive for *Hepatozoon*. Type 2 meronts were found and individual merozoites were found in granulomas, and findings were confirmed by TEM. None of the 11 polecats (*Mustela putorius*), four weasels (*Mustela nivalis*), or two otters (*Lutra lutra*) was infected with *Hepatozoon* [135].

It is evident that *Hepatozoon* infection results in granulomatous lesions in mustelids. *Hepatozoon* meronts were found in the lungs of five of 11 mink (*Mustela vison*) from Canada [136]. Granulomas contained type 2 meronts and individual zoites (merozoites/gamonts). Recently, hepatozoonosis was diagnosed in three minks from the USA [137]. In one of these minks, pneumonitis was associated with type 2 meronts and considered as contributing to the mink’s death. In the second mink, hepatozoonosis was incidental in the lungs and heart. In the third mink, hepatozoonosis was associated with distemper virus infection, which is immunosuppressive. The occurrence of lesions and *Hepatozoon* in lungs is unusual and has been documented previously in Japanese bears infected with *H. ursi* [138] and in minks in Canada [136].

#### Hepatozoonosis in a Pampas gray fox

An intriguing case of hepatozoonosis was described in a Pampas gray fox (*Lycalopex*/*Pseudalopex gymnocercus*) from Argentina [139]. The fox was easily hand-caught because of incoordination and apparent blindness and was euthanized. A complete necropsy was performed, and virtually all organs were examined histologically. The fox had distemper virus infection associated with pneumonia. *Hepatozoon* sp. meronts were found in histological sections of the myocardium and skeletal muscle. Morphologically, the lesions and meronts were similar to onion skin type 2 meronts, so far restricted to *H. americanum* in dogs and coyotes in the USA [27]. The meronts were up to 122 µm long and contained concentric layers of mucopolysaccharide structures, typical of *H. americanum*. Macrophages within heart granolamas and vascular adjacent sinusoidal blood vessels contained merozoites or gamonts. The morphologic diagnosis was confirmed by PCR. However, the parasite was more closely related phylogenetically to *H. felis*; 337-base-pair (bp) sequences were 99% identical to *H. felis* and distinct from *H. canis* and *H. americanum* [139].

#### Hepatozoonosis in red fox

A 2-month-old female red fox was examined at a veterinary clinic in Sarajevo, because of suspected viral infections. It had a fractured leg, was in poor body condition, was dehydrated, and had pale mucous membranes [140]. Evidence for viral infection was not found. The fox died and was necropsied. The lungs were edematous, hemorrhagic, and mildly inflamed. The lymph nodes and spleen were also edematous. Numerous type 2 *Hepatozoon* meronts were found in histological sections of the bone marrow, spleen, lymph nodes, and lungs. Concomitant *Leptospira* infection was present in the kidneys. No other pathogens were found. The extent of parasitism and lesions indicate that *Hepatozoon* sp*.* contribute to the fox’s death [140].

Histological examination of the heart, rear limbs, jejunum, kidneys, liver, lungs, popliteal or axillary lymph nodes, spleen, and tongue from 47 red foxes hunted in Portugal revealed type 2 meronts in the bone marrow of 11 and spleens of two, but not in other tissues [141]. These results suggest that *H. canis* is not an important cause of illness in red foxes.

Fever, emaciation, anemia, splenomegaly, lumbar paralysis, and stiffness were reported in 3 month-old male fox (*Vulpes vulpes schrencki*) in Hokkaido, Japan [142]. The hematological values were within normal limits but large numbers of *Hepatozoon* gamonts were found in blood smears.

#### Hepatozoonosis in coyotes

*Hepatozoon* infections have been reported in coyotes (*Canis latrans*) in the USA (Table 7). Naturally infected coyotes appeared to be in good health [125, 126]. Histologically, they had muscular lesions but not bone lesions. Two coyote pups experimentally fed 100 oocysts of *H. americanum* developed parasitemia and lesions in bones and muscle, associated with typical onion skin-like meronts in muscles [126]. The results of this study were confirmed by Garrett et al. [143]. Four juvenile and five adult coyotes fed 50 oocysts of *H. americanum* developed clinical hepatozoonosis similar to that observed in dogs, irrespective of the age. However, muscular lesions were more severe in juveniles than in adult coyotes, regardless of the *H. americanum* isolate used [143].

#### Hepatozoonosis in raccoons

As stated earlier, inflammatory lesions were seen in the heart, skeletal muscle, and spleen of raccoons from Texas, USA [51]. Recently, hepatozoonosis was diagnosed in three of 117 raccoons from Tennessee, USA (Table 9). Only two of the three PCR-positive raccoons had histopathology available, and both had myocarditis and one had meronts in the heart. Additionally, one raccoon had *Hepatozoon*-like meronts in the heart and severe myocarditis, but PCR was not attempted (personal communication to JPD).

## Molecular detection of *Hepatozoon* spp. in carnivores

PCR is a sensitive and useful technique for the detection of *Hepatozoon* infection and for the identification the infecting species. PCR for *Hepatozoon* spp. of carnivores has relied almost entirely on amplification of segments of the *18S* rRNA gene. Different primer sets have been developed for conventional and real-time PCR [144]. Most primer sets amplify segments of 390–740 bp of the *18S* rRNA gene, although some primers can amplify up to 1760 bp [145]. Phylogenetic analyses and species determination benefit from the amplification of long *18S* rRNA fragments and preferably from the amplification of the whole gene.

PCR protocols are often not specific for *Hepatozoon* spp. and may amplify piroplasmids and other apicomplexan organisms; therefore, it is important to sequence the DNA products and determine the identity of the amplicons [144]. In some cases, it is also necessary to perform a second and more specific PCR to determine whether there is co-infection in the sample, which can often be of *Hepatozoon* sp. with *Babesia* or other apicomplexan spp. [146, 147]. In such cases, the initial non-specific PCR may amplify the more prevalent organism in the sample and not detect the presence of another organism.

There is considerable variability in sequences even within *Hepatozoon* spp. of carnivores, such as variability in *H. americanum* with multiple different *18S* rRNA sequences published for this species, as well as variability within *H. canis* and *H. felis* [94, 111, 128, 148–151]. This variability has led to the definition of several different genotypes within some of the species [111, 128]. These genotypes correspond to sequence polymorphism but usually not to morphologic, pathogenic, host, or vector-associated characteristics.

Quantitative real-time PCR enables quantification of the pathogen in the sample, is highly sensitive, and can be used for detection of infection as well as for follow-up of animals during or after treatment. Several real-time PCR assays have been developed for *Hepatozoon* spp. of carnivores [92, 152, 153].

## Conclusions

We have critically reviewed the taxonomy and biology of *Hepatozoon* infections in the Carnivora. The morphology and life cycles of seven valid species with known merogonic stages (*H. americanum*, *H. canis*, *H. felis*, *H. martis*, *H. rufi*, *H. silvestris*, *H. ursi*) were re-evaluated and are summarized in Table 1 using standard terminology. Information lacking for *H. procyonis * is discussed. The validity of *H. mustelis*, *H. banethi*, and *H. ewingi* is discussed, and we consider them invalid species. We also provide redescriptions of merogonic stages of *H. apri*, *H. martis*, and *H. silvestris* based on reexamination of naturally infected tissues.

## Data Availability

Data supporting the main conclusions of this study are included in the manuscript.

## Abbreviations

AS: Asymptomatic general population
*B.** canis*: *Babesia canis*
C: Cytology, blood smears
D: Domestic
DY: Doxycycline
*E.** canis*: *Ehrlichia canis*
ELISA: Enzyme-linked immunosorbent assay
Eu: Euthanized
FELV: Feline leukemia virus
*18S* rRNA: 18S ribosomal RNA
FIV: Feline immunodeficiency virus
HE: Hematoxylin and eosin
ID: Imidocarb dipropionate
IFA: Indirect fluorescent antibodies
NS: Not stated
P: Park reserve
PAS: Periodic acid–Schiff stain
PCR: Polymerase chain reaction (*18S* rRNA gene)
R: Road death
*R. sanguineus*: *Rhipicephalus sanguineus*
S: Serology
SH: Shot or hunted
SA: Survey of vector-borne pathogens
SE: Shelter
SP: Survey for other pathogens
ST: Stray
T: Trapped or caught
VC: Veterinary clinic
TEM: Transmission electron microscopy
USDA: United States Department of Agriculture
Z: Zoo
